# Solvent and Substituent Size Influence on the Cyclochiral Rigidity of Aminomethylene Derivatives of Resorcin[4]arene

**DOI:** 10.3390/molecules28217426

**Published:** 2023-11-04

**Authors:** Waldemar Iwanek

**Affiliations:** Faculty of Chemical Technology and Engineering, Bydgoszcz University of Technology, Seminaryjna 3, 85-326 Bydgoszcz, Poland; waldemar.iwanek@pbs.edu.pl

**Keywords:** resorcin[4]arene, cyclochiral, reaction path, DFT calculations

## Abstract

Resorcin[4]arenes (R[4]A) are a group of macrocyclic compounds whose peculiar feature is the presence of eight hydroxyl groups in their structure. The directional formation of intramolecular hydrogen bonds with their participation leads to the formation of a cyclochiral racemic mixture of these compounds. Their stability strongly depends on the substituent and especially the environment in which they are located. The paper discusses the cyclochiral nature of aminomethylene derivatives of R[4]A (AMD-R[4]A). Their cyclochiral rigidity in non-polar solvents has been shown. The influence of the size of the alkyl groups in the amino substituents of AMD-R[4]A on their cyclochiral nature was noted. To calculate the reaction paths for their racemization, the nudged elastic band (NEB) method was employed using the semi-empirical DFT (GFN1-xTB) approach. The calculated activation barrier energies for their racemization in chloroform, obtained through various semi-empirical quantum chemical methods (SE), Hartree–Fock (HF), and density functionals theory (DFT), show good correlation with experimental observations. Among the tested methods, the B38LYP-D4 method is highly recommended due to its fast computational speed and accuracy, which is comparable to the time-consuming double-hybrid DH-revDSD-PBEP86 approach.

## 1. Introduction

R[4]A is a type of macrocyclic compound that exhibits stability in its upper rim and crown conformation, which is achieved through a system of intramolecular hydrogen bonds [1]. The strength of these hydrogen bonds and their interactions depend greatly on the polarity and nature of the surrounding environment (solvent). For instance, in chloroform, R[4]A molecules undergo self-assembly into hexamers and octamers [2]. Similar self-assembly behavior has also been observed in toluene [3]. The unique characteristics of these supramolecules, such as their internal closed cavity and distinct electron solvation properties, have sparked interest in studying chemical reactions that can occur within them [4]. The “*ortho*” positions in R[4]A are particularly reactive and can be utilized for modifying the upper rim through processes like the Mannich reaction [5]. This enables the synthesis of aminomethylene derivatives of R[4]A with high yields, utilizing formaldehyde and primary or secondary amines [6,7], amino alcohols, and amino acids [8,9]. These compounds possess intriguing receptor properties towards various organic and biological compounds, making them of interest in different fields [10,11].

On the topic of the derivatives mentioned, the existing literature lacks studies demonstrating a significant relationship between their structure and the polarity/nature of the solvent, as well as the size of the amino groups. This research aims to address this gap by highlighting the cyclochiral nature of these derivatives in non-polar solvents, along with their pronounced stabilization when using amines with branched alkyl groups. Theoretical calculations were conducted to determine the energy barriers associated with the racemization of enantiomers of these derivatives, and the mechanisms involved were thoroughly discussed.

The discussed AMD-R[4]A molecules contain 150–200 atoms in their structure, and as a result of this, DFT calculations are very time-consuming, especially geometry optimization and accurate determination of energy. Moreover, dispersion effects, as the particle size increases, play an increasingly important role in both geometry optimization and accurate energy calculations. In the literature, there is no assessment of SE and DFT methods in relation to R[4]A and its derivatives in terms of the assessment of reaction energy, the height of reaction barriers, or non-covalent interactions. For this reason, the author decided to test several SE methods most frequently used in the literature (PM6-D3H4 [12], GFN2-xTB [13], and AIQM1 [14]), the HF-D3-ACP method [15] due to its parameterization in the field of thermochemistry and reaction barriers, and fast DFT-3c composite methods (B97-3c [16], r2scan-3c [17], PBEh-3c [18]), as well as several DFT methods for calculating reaction barriers, i.e., B38LYP-D4 [19], M062X [20], wB97M-V [21], and DH-revDSD-PBEP86-D4 [22].

## 2. Results and Discussion

**Figure 1:**
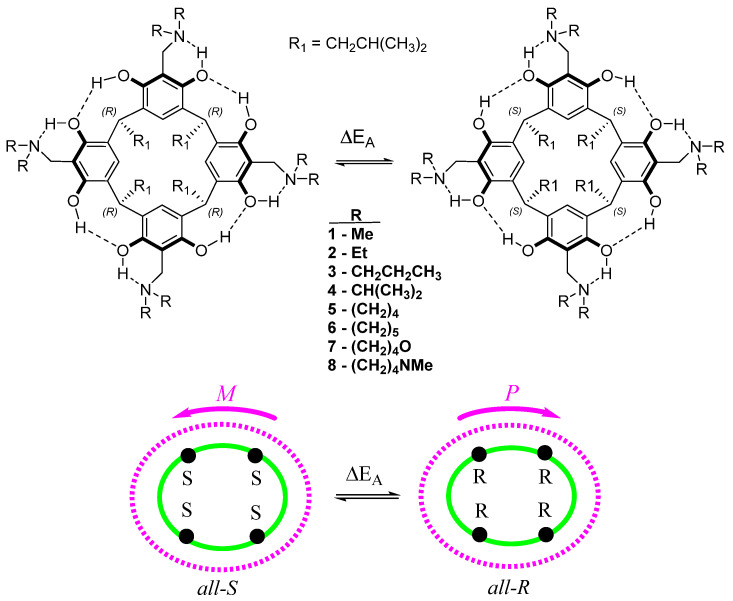
The structure of the cyclochiral AMD-R[4]A with a schematic picture of the cyclochiral nature of these derivatives. The color green represents the lower rim with stereogenic centers, while purple represents the hydrogen bonds in the upper rim of AMD-R[4]A. The symbols *M* and *P* indicate the direction of the hydrogen bonds, which ultimately determine the enantiomers of these compounds.

AMD-R[4]A derivatives were synthesized through the Mannich reaction of R[4]A with formaldehyde and various secondary amines in ethanol. The secondary amines used include N,N-dimethylamine (**1**), N,N-diethylamine (**2**), N,N-dipropylamine (**3**), N,N-diisopropylamine (**4**), pyrrolidine (**5**), piperidine (**6**), morpholine (**7**), and N-methylpiperazine (**8**). These derivatives are stabilized by a system of eight intramolecular hydrogen bonds in non-polar environments. Their directionality (*M*, *P*) affects the generation of stereogenic centers (*S* or *R*) on the methine carbons connecting the resorcinol rings in R[4]A. Figure 1 illustrates the structures of the described AMD-R[4]A molecules, with the lower part of the figure symbolically representing their cyclochirality. The color green represents the lower rim with stereogenic centers, while purple represents the hydrogen bonds in the upper rim of AMD-R[4]A. The symbols *M* and *P* indicate the direction of the hydrogen bonds, which ultimately determine the enantiomers of these compounds.

**Figure 2:**
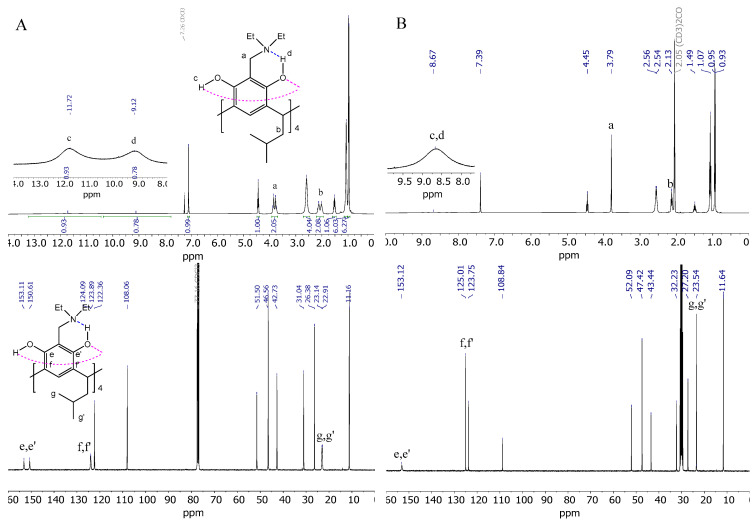

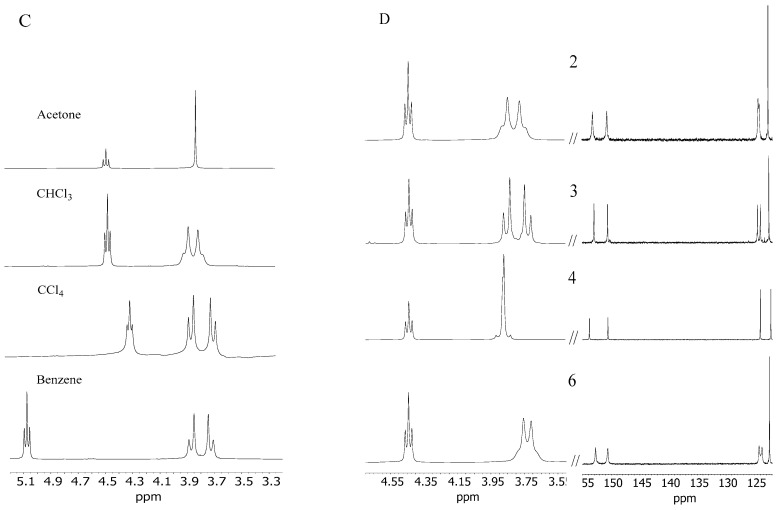
(**A**) 1H and 13C NMR spectrum of derivative **2** in CHCl_3_; (**B**) 1H and 13C NMR spectrum of derivative **2** in acetone; (**C**) spin–spin coupling comparison of the methylene group (a) derivative **2** in acetone, CHCl_3_, CCl_4,_ and benzene; (**D**) comparison of fragments of 1H and 13C NMR spectra in CHCl_3_ of AMD-R[4]A derivatives: **2**, **3**, **4**, **6**, respectively.

The NMR spectra of AMD-R[4]A exhibit notable variations when recorded in solvents of varying proton donor–acceptor properties and polarity. Figure 2 illustrates an example of the 1H and 13C NMR spectra of derivative **2**, obtained in CDCl_3_ and acetone-d_6_ solvents.

In chloroform (Figure 2A), we observe diastereotopic methylene protons -CH_2_- (a) in the form of slightly fuzzy doublets and diastereotopic methylene protons (b) in the lower rim of the derivative **2**. This phenomenon arises from the directional rigidity of the upper rim, which is caused by a strong hydrogen bond forming between the amino group and the adjacent hydroxyl group proton (d) in the resorcinol ring (Et_2_N^…^HO-Ph). This feature of these compounds determines their cyclochirality. The NMR spectrum also reveals two types of hydrogen bonding within the chemical shift range of d = 8–14 ppm. One of them was mentioned above, the second type of hydrogen bonding (c) between the hydroxyl groups of the resorcinol rings (Ph-HO^…^HO-Ph) that stabilizes the crown conformation of derivative **2**. The cyclochiral rigidity and directional arrangement of the amino groups can also be observed in the 13C NMR spectrum. This is reflected in the different chemical shifts of carbons e and e′ (153.1 and 150.6 ppm, respectively) and f, f′ (124.1 and 123.9 ppm) of resorcinol rings as well as carbons g, g′ (23.1, 22.9 ppm) of methyl groups (CH_3_) in the lower rim of the derivative **2**.

In the presence of acetone (Figure 2B), it can be observed that all the protons discussed in the 1H NMR spectrum, as well as the carbon atoms in the 13C NMR spectrum, appear as singlets. Additionally, the hydroxyl group protons are observed as an extended singlet, exhibiting a chemical shift value of d = 8.67 ppm. This can be attributed to the strong solvation of the derivative **2** in acetone, which probably results in the breaking of intramolecular hydrogen bonds of the amino group with the hydroxyl group in favor of the formation of intermolecular hydrogen bonds of the hydroxyl groups with the oxygen atom of the acetone molecule.

Molecule **2** becomes even more rigid in non-polar solvents such as CCl_4_ and benzene (Figure 2C). In these solvents, for methylene protons (a), we observe a doublet of doublets, characteristic of cyclic rigid structures such as oxazine or boron derivatives of R[4]A [23,24].

**Scheme 1:**
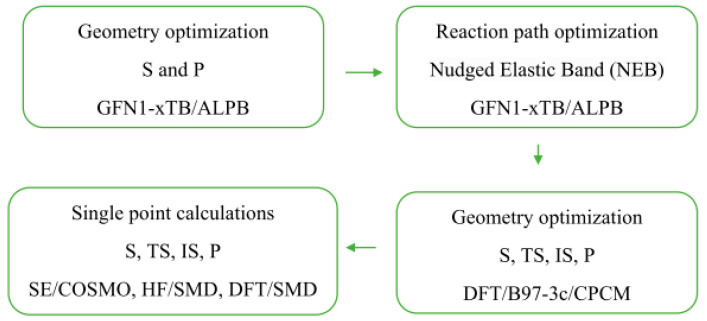
Procedure used for geometry optimization and one-point energy calculations of substrates (S), transition states (TS), intermediate states (IS), and products (P) of AMD-R[4]A racemization reaction.

It was also observed that AMD-R[4]A derivatives exhibit cyclochiral stiffness, which becomes more pronounced with the increasing size of the alkyl groups on the amino substituents. In the chloroform NMR spectrum, derivative **3** with a dipropylamino group displays a classically resolved doublet of proton doublets for the methylene group (a) (Figure 2D). This proves the special rigidity of this structure in this solvent, which is also confirmed by the 13C NMR spectrum and separated signals of carbons e, e′ and f, f′. Additional fragments of the 1H and 13C NMR spectra for derivatives **2**, **4**, and **6** are presented, depicting the methylene group -CH_2_- (a) and the chemical shifts of carbon atoms (e, e′ and f, f′). Conversely, the remaining AMD-R[4]A derivatives exhibit an extended singlet of protons for the methylene group and strongly broadened signals for the e, e′ and f, f′ carbons (see Supplementary Materials). Given the rigid cyclochiral structure of derivatives **2**, **3**, **4**, and **6** in non-polar solvents, several intriguing questions arise: How stable are these structures in chloroform? What is the racemization barrier for these compounds, and what is the accompanying racemization mechanism? To answer these questions, the NEB method [25] was used to obtain saddle points (SP) and intermediate state point (IS) as well as minimum energy paths between known initial (*M*-AMD-R[4]A) and final states (*P*-AMD-R[4]A). Optimization of the geometry of the generated structures along the reaction path in CHCl_3_ was carried out using the fast semi-empirical DFT method—GFN1-xTB [26] in CHCl_3_ with the analytical linearized Poisson–Boltzmann (ALPB) solvent model [27]. The SP and IS structures obtained by this method were further subjected to geometrical optimization using the DFT B97-3c method to determine the transition states (TS), intermediate state (IS), substrate (S), and product (P) structures, utilizing the conductor-like polarizable continuum model (CPCM) solvent model [28] for chloroform. Frequency calculations were then performed to confirm one imaginary and appropriate frequency for each TS, while no imaginary frequency for all local minima. The optimized geometrical structures were then employed for single-point energy (SPE) calculations in chloroform, employing semi-empirical (SE) methods, the Hartree–Fock method (HF-D3), and DFT methods with the universal solvation model (SMD) [29] for HF and DFT methods. For the SE methods, SPE calculations were performed using suitable solvent models: PM6-D3H4/COSMO, GFN2-xTB/ALPB, and AIQM1 in the gas phase. Scheme 1 illustrates the procedure for optimizing the geometry and conducting single-point energy calculations for individual structures.

**Figure 3:**
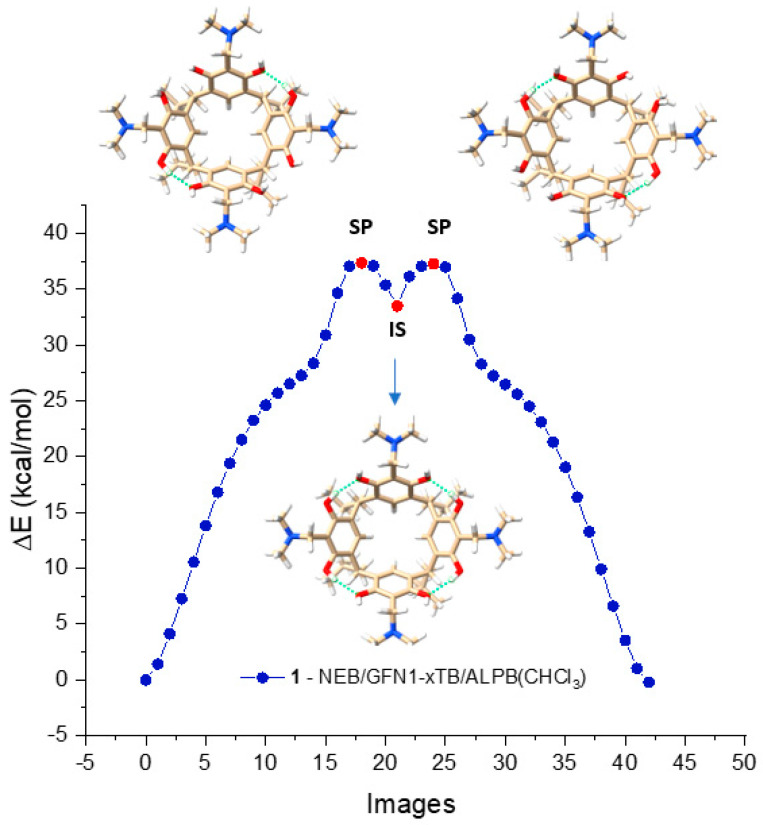
Racemization reaction path of derivative **1** in CHCl_3_ calculated by NEB/xTB1 presented in the form of 43 geometrically optimized images. The images marked in red represent the structures of the saddle points (SP) and the intermediate state (IS) point.

The NEB/GFN1-xTB method generated a reaction path for derivative **1** in CHCl_3,_ depicted in Figure 3. The path consists of 43 images, with the highest points representing the geometrical structures SP, and the lowest point representing the geometrical structure IS. The optimized structures of these points, indicated in red, are displayed above and below the energy change curve, as seen in the figure. As the curve shows, it is evident that the racemization reaction of *M*-AMD-R[4]A to *P*-AMD-R[4]A occurs in two steps and involves a non-chiral intermediate state (IS).

**Figure 4:**
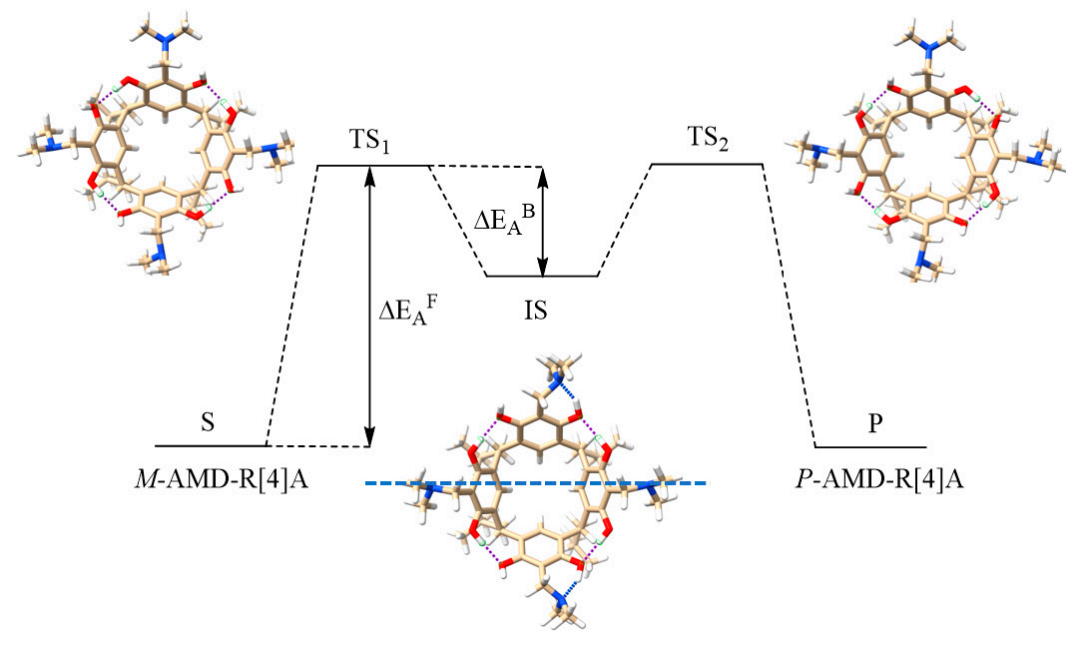
Activation energies of ΔE_A_^F^ racemization and back reaction ΔE_A_^B^ along with geometrically optimized by B97-3c method in CHCl_3_ structures of TS_1_, TS_2,_ and IS for derivative **1**.

The SP and IS structures found by this method served as input structures for geometric optimization by the DFT B97-3c method of the TS and IS structures and subsequent calculations of the activation energy of the racemization reaction (ΔE_A_^F^) and the activation energy of the back reaction (ΔE_A_^B^). Figure 4 visually represents these energy values and also showcases the CHCl_3_-optimized structures of TS and IS for derivative **1** obtained through the B97-3c calculations.

Theoretical calculations have confirmed that the structures of the TS_1_ and TS_2_ states exhibit mirror symmetry, and the orientation of the hydrogen bond in the transition state aligns with that of the initial states. In transition states, only intramolecular hydrogen bonds between hydroxyl groups are preserved, while intramolecular hydrogen bonds between amino groups and hydroxyl groups are not observed. In the intermediate state (IS), hydrogen bonds are reorganized, and hydrogen bonds are formed between the hydroxyl groups of two opposite resorcinol units with adjacent resorcinol units. Additionally, intramolecular hydrogen bonds form between the amino groups and hydroxyl groups in the opposing resorcinol units, resulting in a structure with a plane of symmetry, as indicated by the dotted blue line in Figure 4.

Table 1 presents the activation energies (ΔE_A_^F^ and ΔE_A_^B^) in kcal/mol for AMD-R[4]A derivatives. The calculations were performed using selected HF methods and DFT on geometries obtained from the B97-3c method. The activation energies determined with the B38LYP-D4 method were found to be closest to the reference values obtained from the DH-revDSD-PBEP86-D4 double-hybrid functional. The largest absolute error of this method for the racemization activation energy ΔE_A_^F^ = 0.82 kcal/mol was found for derivative **4**, while for the activation energy of the back reaction ΔE_A_^B^ = 0.44 kcal/mol, this was found for derivative **3**. It is worth noting that the B38LYP calculations were significantly faster, approximately twenty times faster, compared to the double-hybrid method when performed with the same number of processors. Similarly, the DFT method using the r2scan-3c functional was approximately sixty times faster than the double-hybrid method. For this method, the largest absolute deviations from the reference values were as follows: ΔE_A_^F^ = 3.28 kcal/mol for derivative **6** and ΔE_A_^B^ = 0.82 kcal/mol for derivative **7**. In turn, calculations using the M062X functional gave the largest deviations ΔE_A_^B^ = 3.78 kcal/mol and ΔE_A_^B^ = 1.27 kcal/mol for derivative **4**.

The HF-D3/6-31G(d)-ACP method, which employs a specially trained 6-31G(d)-ACP basis for thermochemical calculations and energy barrier height determination, has proven to be competitive. Notably, calculations using this method are significantly faster than those using a double-hybrid approach. The largest discrepancies in activation energy calculations were observed for derivative **2**, with values of ΔE_A_^F^ = 3.36 kcal/mol and ΔE_A_^B^ = 1.98 kcal/mol. In contrast, the discrepancies for the other derivatives were much smaller compared to the reference values.

Among the SE methods listed in Table 2, the AIQM1 method exhibits results that closely resemble the double-hybrid method, even though the calculations were performed in the gas phase. The maximum error obtained using AIQM1 was ΔE_A_^F^ = 3.10 kcal/mol for derivative **7** and ΔE_A_^B^ = 4.82 kcal/mol for derivative **4**. In the case of the PM6-D3H4 and GFN2-xTB methods, significant discrepancies were observed for both activation energies in comparison to the reference values.

**Figure 5:**
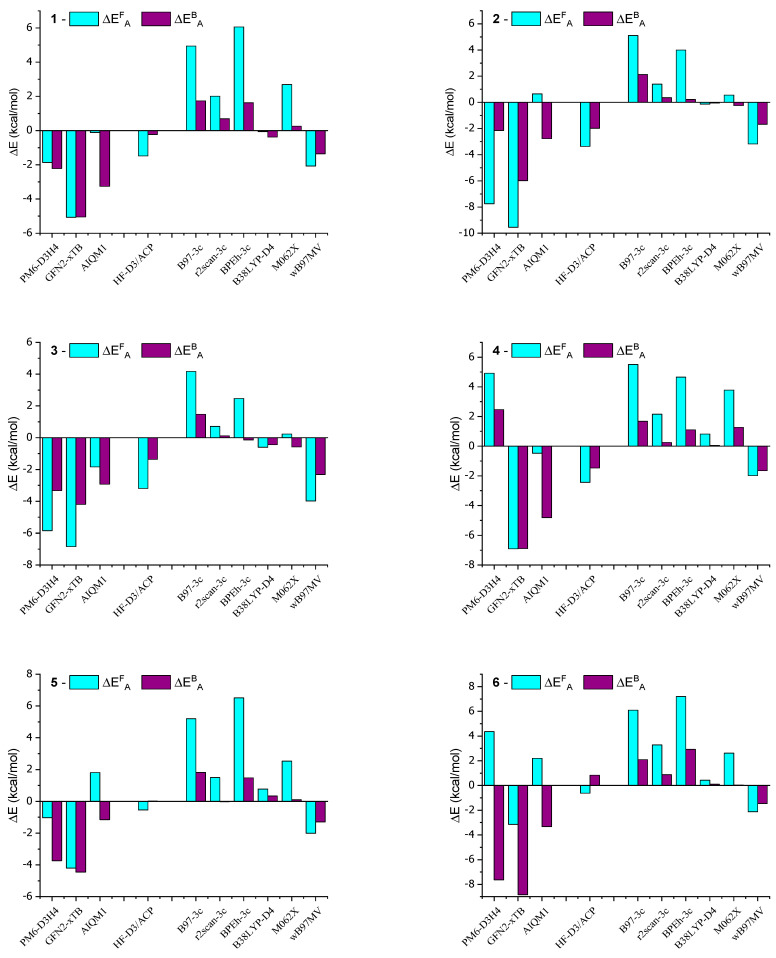

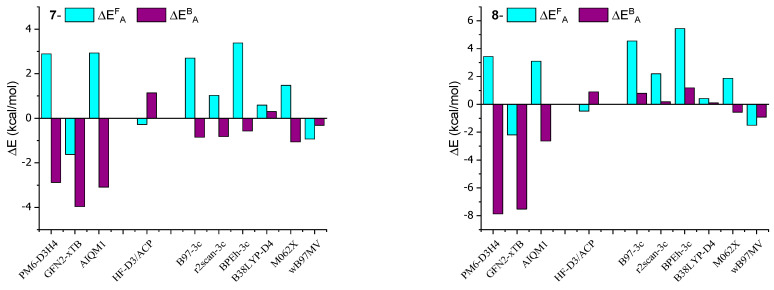
Bar chart of relative deviations (kcal/mol) from reference values obtained using the DH− revDSD−PBEP86−D4 functional for the data in Table 1 and Table 2.

In order to better visualize the obtained results, deviations from the reference values are presented in Figure 5 in the form of a bar graph of the data contained in Table 1 and Table 2.

The calculations presented in this study align with the experimentally observed trend of increased stiffness in AMD-R[4]A molecules as the alkyl groups of the amino substituents become more branched. The activation energy barriers for racemization increase from derivative **1** through derivative **2** and **3** to derivative **4**, with values of ΔE_A_^F^ (kcal/mol) as follows: 33.59, 37.63, 38.87, and 41.26. This trend is also reflected in the 1H and 13C NMR spectra, where extended signals of diastereotopic -CH_2_- protons and separated signals of carbon atoms bound to hydroxyl groups (e, e′) are observed. Derivatives **5** and **6** contain cyclic amines in their structure, pyrrolidine and piperidine, respectively. Their two closest carbon atoms to the nitrogen atom are at a similar distance as in derivative **1** (NMe_2_ group). The remaining carbon atoms are significantly distant from the aromatic part of R[4]A, which results in very small dispersion interactions. This is probably the reason for the comparable values of their activation energies ΔE_A_^F^ (kcal/mol) for the derivatives: **1** = 33.59; **5** = 33.35; **6** = 34.33. Surprisingly low barriers to racemization activation and the back reaction were found for the morpholine derivative **7**, with values of ΔE_A_^F^ = 15.94 kcal/mol and ΔE_A_^F^ = 5.11 kcal/mol. Similarly, for derivative **8**, which also contains nitrogen in the cyclic amino group, the calculated activation barriers are lower compared to amino substituents without heteroatoms. This suggests a preliminary conclusion that the presence of a heteroatom in a cyclic secondary amine reduces the activation energy of cyclochiral racemization for such derivatives, as well as the barrier for the back reaction.

Experimental findings (Figure 2C) reveal that molecule **2** exhibits increased rigidity in non-polar solvents such as benzene and CCl_4_ compared to CHCl_3_. This observation was further supported by calculations using the B38LYP-D4 method, which determined the height of the racemization barriers (ΔE_A_^F^) in benzene and CCl_4_ using the SMD solvent model. The calculated racemization activation energies are: in benzene ΔE_A_^F^ = 42.69 kcal/mol, in CCl_4_ ΔE_A_^F^ = 42.64 kcal/mol, and are more than 5.1 kcal/mol higher than the activation barrier in CHCl_3_ (ΔE_A_^F^= 37.50 kcal/mol). However, we do not observe practically any change in the activation energy of the back reaction, which for benzene and CCl_4_ are ΔE_A_^B^= 15.36 kcal/mol and ΔE_A_^B^ = 15.26 kcal/mol, respectively, while for CHCl_3_ it is ΔE_A_^B^ = 15.31 kcal/mol.

The observed significant decrease in rigidness in acetone can likely be attributed to the increased involvement of the hydroxyl groups of molecule **2** in the formation of hydrogen bonds with polar acetone molecules, as opposed to hydrogen bonding with the nitrogen atom of amino groups. Thus, the change in the hydrogen bond system from intramolecular (non-polar solvents) to intermolecular (polar solvents containing heteroatoms) is responsible for the substantial reduction in the activation energy barrier for cyclochiral racemization of the studied derivatives.

## 3. Experimental Section

The NMR spectra were achieved using an Avance 400 ultra-shield spectrometer (Bruker, Karlsruhe, Germany). Reagents and solvents were obtained from Fluka (Buchs, Switzerland), and Merck (Darmstad, Germany) and were used without purification.

*Calculation procedure*: All NEB and DFT calculations were performed with the ORCA 5.0.3 and 5.04 software package [30] with default setting. For the B97-3c, r2scan-3c, and PBEh-3c methods, built-in base sets were used for calculations. For the M062X functionals, the 6-311G(d,p) basis was used, for the wB97M-V, the def2-TZVP basis. In the case of the B38LYP-D4 functional, the def2-mTZVP base and the D4 dispersion parameters from work [19] were used. Calculations with the DH-revDSDPBEP86-D4 functional were carried out with the def2-TZVPP basis recommended in [22] and the D4 dispersion parameters. The HF-D3-ACP calculations were performed with Gaussian 16 software packages [31] with 6-31G(d)-ACP basis and D3 dispersion parameters as in work [15]. GFN1-xTB calculations were performed with the development version xtb v. 6.5.0 [32]. The PM6-D3H4 were conducted with the MOPAC program version 21.237W [33]. The AIQM1 calculations were performed online on the MLatom@XACS cloud service [34].

*AMD-R[4]A synthesis procedure:* A total of 0.2 g (0.225 mmol) of R[4]A was weighed into a round-bottomed flask and dissolved in 10 mL of ethanol. Then 5 equivalents of formaldehyde and 5 equivalents of sec-amine were added and left at room temperature for 24 h. The precipitate was then filtered, washed with cold ethanol, and dried. The yields of AMD-R[4]A derivatives formation ranged from 66% to 85%.

*15,35,55,75-tetrakis((dimethylamino)methyl)-2,4,6,8-tetraisobutyl-1,3,5,7(1,3)-tetrabenzena-cyclooctaphan-14,16,34,36,54,56,74,76-octaol* (**1**), (66% yield), white solid. ^1^H NMR (400 MHz, CDCl_3_, T = 298 K) δ [ppm]: 8.68 (br. s., 8H, OH), 7.11 (s, 4H, PhH), 4.43 (t, *J* = 7.70 Hz, 4H, CH), 3.71 (s, 8H, CH_2_), 2.29 (s, 24H, CH_3_), 2.07 (m, 8H, CH_2_), 1.51 (m, 4H, CH), 0.97 (d, *J* = 6.60 Hz, 24H CH_3_); ^13^C NMR (100 MHz, CHCl_3_, T = 298 K) δ [ppm]: 152.4, 150.8, 124.0, 122.5, 108.2, 56.9, 44.5, 42.9, 31.1, 26.4, 23.0.

*15,35,55,75-tetrakis((diethylamino)methyl)-2,4,6,8-tetraisobutyl-1,3,5,7(1,3)-tetrabenzena-cyclooctaphan-14,16,34,36,54,56,74,76-octaol* (**2**), (72% yield), white solid. ^1^H NMR (400 MHz, CDCl_3_, T = 298 K) δ [ppm]: 11.85 (br.s., 4H, OH), 9.15 (br.s., 4H OH), 7.10 (s, 4H, PhH), 4.44 (t, *J* = 7.70 Hz, 4H, CH), 3.81(dd, *J* = 14.30 Hz, 8H, CH_2_), 2.59 (br. s., 16H, CH_2_), 2.12 (m, 4H, CH_2_), 2.02(m, 4H, CH_2_), 1.52 (m, 4H, CH), 1.07 (br. s., 24H, CH_3_), 0.97 (d, *J* = 6.60 Hz, 24H, CH_3_); ^13^C NMR (100 MHz, CHCl_3_, T = 298 K) δ [ppm]: 152.9, 150.4, 123.9, 123.7, 122.2, 107.9, 51.3, 46.4, 42.6, 30.9, 26.2, 23.0, 22.7, 11.0.

*15,35,55,75-tetrakis((diprophylamino)methyl)-2,4,6,8-tetraisobutyl-1,3,5,7(1,3)-tetrabenzena-cyclooctaphan-14,16,34,36,54,56,74,76-octaol* (**3**), (68% yield), white solid. ^1^H NMR (400 MHz, CDCl_3_, T = 298 K) δ [ppm]: 11.45 (br. s., 4H, OH), 8.86 (br.s., 4H, OH), 7.12 (s, 4H, PhH), 4.44 (t, *J* = 7.70 Hz, 4H, CH), 3.79 (dd, *J* = 14.30 Hz, 8H, CH_2_), 2.45 (br.s., 16H, CH_2_), 2.15 (m, 4H, CH_2_), 2.00 (m, 4H, CH_2_), 1.52 (m, 20H, CH, CH_2_), 0.97 (t, *J* = 5.87 Hz, 24H, CH_3_), 0.87 (t, *J* = 6.97 Hz, 24H, CH_3_); ^13^C NMR (100 MHz, CHCl_3_, T = 298 K) δ [ppm]: 152.9, 150.5, 124.3, 123.8, 122.3, 108.2, 55.7, 52.9, 42.8, 31.0, 26.3, 23.2, 22.8, 19.6, 11.9.

*15,35,55,75-tetrakis((diisoprophylamino)methyl)-2,4,6,8-tetraisobutyl-1,3,5,7(1,3)-tetra-benzenacyclooctaphan-14,16,34,36,54,56,74,76-octaol* (**4**), (68% yield), white solid. ^1^H NMR (400 MHz, CDCl_3_, T = 298 K) δ [ppm]: 9.24 (s, 4H, OH), 7.05 (s, 4H, PhH), 4.44 (t, *J* = 7.70 Hz, 4H, CH), 3.87 (dd, *J* = 16.40 Hz, 8H, CH_2_), 3.18, br. s., 4H, CH), 3.05 (br. s., 4H, CH), 2.10 (m, 4H, CH_2_), 2.00 (m, 4H, CH), 1.52 (m, 4H, CH), 1.18 (br. s., 12H, CH_3_), 1.11 (br. s., 12H, CH_3_), 1.02 (br. s., 24H, CH_3_), 0.96 (m, 24H, CH_3_); ^13^C NMR (100 MHz, CHCl_3_, T = 298 K) δ [ppm]: 153.8, 150.6, 123.8, 122.0, 116.5, 108.2, 48.7, 43.3, 42.6, 30.9, 26.3, 23.2, 22.8, 20.7, 19.2.

*15,35,55,75-tetrakis((pirrolidin)methyl)-2,4,6,8-tetraisobutyl-1,3,5,7(1,3)-tetrabenzenacyclo-octaphan-14,16,34,36,54,56,74,76-octaol* (**5**), (74% yield), white solid. ^1^H NMR (400 MHz, CDCl_3_, T = 298 K) δ [ppm]: 9.73 (s, 8H, OH), 7.12 (s, 4H, PhH), 4.46 (t, *J* = 7.70 Hz, 4H, CH), 3.90 (s, 8H, CH_2_), 2.66 (br. s., 16H, CH_2_), 2.10 (br. s., 8H, CH_2_), 1.84 (s, 16H, CH_2_), 1.54 (m, 4H, CH), 1.00 (d, *J* = 6.60 Hz, 24H, CH_3_); ^13^C NMR (100 MHz, CHCl_3_, T = 298 K) δ [ppm]: 152.8, 150.1, 124.0, 122.3, 108.7, 53.7, 53.1, 42.7, 31.1, 26.4, 23.8, 23.0.

*15,35,55,75-tetrakis((piperidin)methyl)-2,4,6,8-tetraisobutyl-1,3,5,7(1,3)-tetrabenzenacyclo-octaphan-14,16,34,36,54,56,74,76-octaol* (**6**), (74% yield), white solid. ^1^H NMR (400 MHz, CDCl_3_, T = 298 K) δ [ppm]: 11.98 (br.s., 4H, OH), 9.00 (br.s., 4H, OH), 7.10 (s, 4H, PhH), 4.44 (t, *J* = 7.70 Hz, 4H, CH), 3.73 (br. q., 8H, CH_2_), 2.91 (br. s., 8H, CH_2_), 2.12 (br.s., 8H, CH_2_), 2.02 (br. s., 8H, CH_2_), 1.60 (br. s., 24H, CH_2_), 1.52 (m, 4H, CH), 0.97 (d, *J* = 6.60 Hz, 24H, CH_3_); ^13^C NMR (100 MHz, CHCl_3_, T = 298 K) δ [ppm]: 152.6, 150.4, 124.1, 123.6, 122.3, 107.6, 55.9, 53.7, 42.6, 30.9, 26.2, 25.6, 23.9, 23.0, 22.7.

*15,35,55,75-tetrakis((morpholin)methyl)-2,4,6,8-tetraisobutyl-1,3,5,7(1,3)-tetrabenzenacyclo-octaphan-14,16,34,36,54,56,74,76-octaol* (**7**), (85% yield), white solid. ^1^H NMR (400 MHz, CDCl_3_, T = 298 K) δ [ppm]: 8.87 (br. s., 8H, OH), 7.11 (s, 4H, PhH), 4.42 (t, *J* = 7.70 Hz, 4H, CH), 3.73 (m, 24H, CH_2_), 2.52 (br. s., 16H, CH_2_), 2.06 (s, 8H, CH_2_), 1.49 (m, 4H, CH), 0.97 (d, *J* = 6.60 Hz, 24H, CH_3_); ^13^C NMR (100 MHz, CHCl_3_, T = 298 K) δ [ppm]: 151.4, 124.0, 122.9, 107.4, 66.8, 55.5, 52.9, 42.7, 31.1, 26.4, 23.0.

*15,35,55,75-tetrakis((4-methylpiperazin)methyl)-2,4,6,8-tetraisobutyl-1,3,5,7(1,3)-tetra-benzenacyclooctaphan-14,16,34,36,54,56,74,76-octaol* (**6**), (71% yield), white solid. ^1^H NMR (400 MHz, CDCl_3_, T = 298 K) δ [ppm]: 8.31 (br. s., 8H, OH), 7.10 (s, 4H, PhH), 4.42 (t, *J* = 7.70 Hz, 4H, CH), 3.77 (s, 8H, CH_2_), 2.70 (br. s., 32H, CH_2_), 2.30 (s, 12H, CH_3_), 2.06 (br. s., 8H, CH_2_), 1.49 (m, 4H, CH), 0.97 (d, *J* = 6.60 Hz, 24H, CH_3_); ^13^C NMR (100 MHz, CHCl_3_, T = 298 K) δ [ppm]: 152.1, 150.7, 124.3, 123.7, 122.6, 107.6, 55.1, 54.8, 52.5, 46.0, 42.7, 31.0, 26.3, 23.0.

## 4. Conclusions

The article investigates the impact of solvent polarity and the size of the amino groups on the cyclochiral behavior of AMD-R[4]A. They display increased rigidity of R[4]A cyclic systems. Furthermore, it was noted that amino substituents with branched amino groups, such as derivatives **3** and **4**, already exhibit rigidity similar to cyclic systems, even in CHCl_3_. On the other hand, in polar solvents such as acetone, AMD-R[4]A derivatives do not show rigidity at room temperature.

The *M–P* racemization reaction paths of AMD-R[4]A derivatives were calculated using the NEB/GFN1-xTB/ALPB(CHCl_3_) method, and the activation energies accompanying this reaction were calculated using selected SE, HF, and DFT methods. The structures of the *P* and *M* enantiomers and the TS and IS states were optimized by DFT B97-3c in CHCl_3_. The activation energies calculated by the double-hybrid method DH-revDSD-PBEP86-D4 in CHCl_3_ using the SMD solvent model were used as reference values. The calculated activation energies for amino substituents without heteroatoms in the cyclic correlate well with the experimentally observed rigidity of those derivatives for which the activation energy of the racemization reaction increases in the series from derivative **1** to **4**. Theoretical confirmation is also supported by the observation that cyclochiral rigidity increases with decreasing solvent polarity.

The obtained results allow for a better understanding of the cyclochiral nature of DMA-R[4]A derivatives and draw attention to this process in relation to other R[4]A derivatives. At the same time, the obtained results confirm that changing the nature of the solvent and its polarity has a significant impact on the behavior of R[4]A derivative molecules. The development in recent years of rapid potential energy screening methods allows for a better understanding of reaction paths (for example racemization) and reaction mechanisms involving these compounds.

## Figures and Tables

**Table 1:** Activation energies ΔE_A_^F^ and ΔE_A_^B^ (kcal/mol) for AMD-R[4]A derivatives, respectively, calculated using selected HF and DFT methods on geometries calculated using the B97-3c method. The reference values obtained with the DH-revDSD-PBEP86-D4 method are marked in blue, the B38LYP-D4 method recommended for calculations in violet.

AMD-R[4]A/ΔE		HF-D3-ACP	B97-3c	r2scan-3c	PBEh-3c	B38LYP-D4	M062X	wB97M-V	DH-revDSD-PBEP86-D4
**1**	ΔE_A_^F^	32.11	38.53	35.60	39.65	33.53	36.28	31.52	33.59
	ΔE_A_^B^	14.83	16.80	15.75	16.69	14.68	15.32	13.70	15.06
**2**	ΔE_A_^F^	34.27	42.74	39.02	41.63	37.50	38.19	34.45	37.63
	ΔE_A_^B^	13.39	17.49	15.73	15.59	15.31	15.13	13.69	15.37
**3**	ΔE_A_^F^	35.68	43.04	39.58	41.34	38.27	39.10	34.89	38.87
	ΔE_A_^B^	18.36	21.19	19.84	19.58	19.28	19.14	17.40	19.72
**4**	ΔE_A_^F^	38.82	46.77	43.42	45.92	42.08	45.04	39.28	41.26
	ΔE_A_^B^	14.63	17.78	16.35	17.20	16.15	17.37	14.45	16.10
**5**	ΔE_A_^F^	32.81	38.55	34.85	39.86	34.14	35.88	31.34	33.35
	ΔE_A_^B^	15.00	16.80	14.96	16.46	15.32	15.09	13.69	14.98
**6**	ΔE_A_^F^	33.70	40.42	37.61	41.53	34.75	36.94	32.20	34.33
	ΔE_A_^B^	22.96	24.21	23.00	25.05	22.23	22.26	20.67	22.13
**7**	ΔE_A_^F^	15.66	18.64	16.96	19.32	16.53	17.43	15.01	15.94
	ΔE_A_^B^	6.25	4.26	4.29	4.54	5.41	4.05	4.79	5.11
**8**	ΔE_A_^F^	23.44	28.48	26.12	29.37	24.34	25.80	22.43	23.93
	ΔE_A_^B^	13.31	13.21	12.60	13.60	12.52	11.85	11.50	12.42

**Table 2:** Activation energies ΔE_A_^F^ and ΔE_A_^B^ (kcal/mol) for AMD-R[4]A derivatives, respectively, calculated with selected SE methods on geometries calculated with the B97-3c method.

AMD-R[4]A/ΔE		PM6-D3H4	GFN2-xTB	AIQM1
**1**	ΔE_A_^F^	31.73	28.52	33.48
	ΔE_A_^B^	12.84	10.01	11.80
**2**	ΔE_A_^F^	29.88	28.08	38.28
	ΔE_A_^B^	13.22	9.39	12.60
**3**	ΔE_A_^F^	33.02	32.03	37.03
	ΔE_A_^B^	16.39	15.52	16.79
**4**	ΔE_A_^F^	46.18	34.36	40.78
	ΔE_A_^B^	18.56	9.21	11.28
**5**	ΔE_A_^F^	32.21	29.15	35.16
	ΔE_A_^B^	11.25	10.53	13.83
**6**	ΔE_A_^F^	38.69	31.18	36.52
	ΔE_A_^B^	14.49	13.30	18.80
**7**	ΔE_A_^F^	18,83	14.32	18.87
	ΔE_A_^B^	2.23	1.15	2.02
**8**	ΔE_A_^F^	27.36	21.73	27.03
	ΔE_A_^B^	4.56	4.90	9.78

## Data Availability

All experimental data and calculations are provided in the Supplementary Materials.

## Supplementary Materials

The following supporting information can be downloaded at: https://www.mdpi.com/article/10.3390/molecules28217426/s1, Supplementary Materials File for full experimental data: NMR spectra of AMD-R[4]A and coordinates of the optimized structures in CHCl_3_.
