# Supplementary material for: Solvent and Substituent Size Influence on the Cyclochiral Rigidity of Aminomethylene Derivatives of Resorcin[4]arene
Source: Molecules. 2023 Nov 4;28(21):7426. doi: 10.3390/molecules28217426 (PMC10649110; doi:10.3390/molecules28217426)
Supplement: Supplementary file 1 [file molecules-28-07426-s001.zip › molecules-2655121-supplementary.pdf]

Solvent and substituent size influence on the cyclochiral rigidity of aminomethylene derivatives of resorcin[4]arene

Waldemar Iwanek

Bydgoszcz University of Technology, Faculty of Chemical Technology and Engineering, Seminaryjna 3, 85-326 Bydgoszcz, Poland.

E-mail: Waldemar.Iwanek@pbs.edu.pl

Supporting information

1. NMR spectra of AMD-R[4]A.
2. The xyz coordinates of the optimized structures in $CHCl_3$.

1. NMR spectra of AMD-R[4]A.

NMR Spectra of **1**

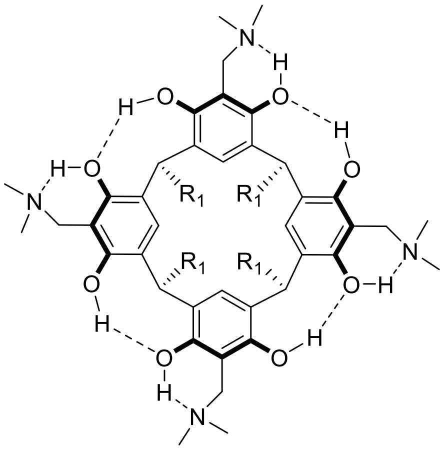

R_1 = $CH_2CH(CH_3)_2$

1H NMR – $CDCl_3$

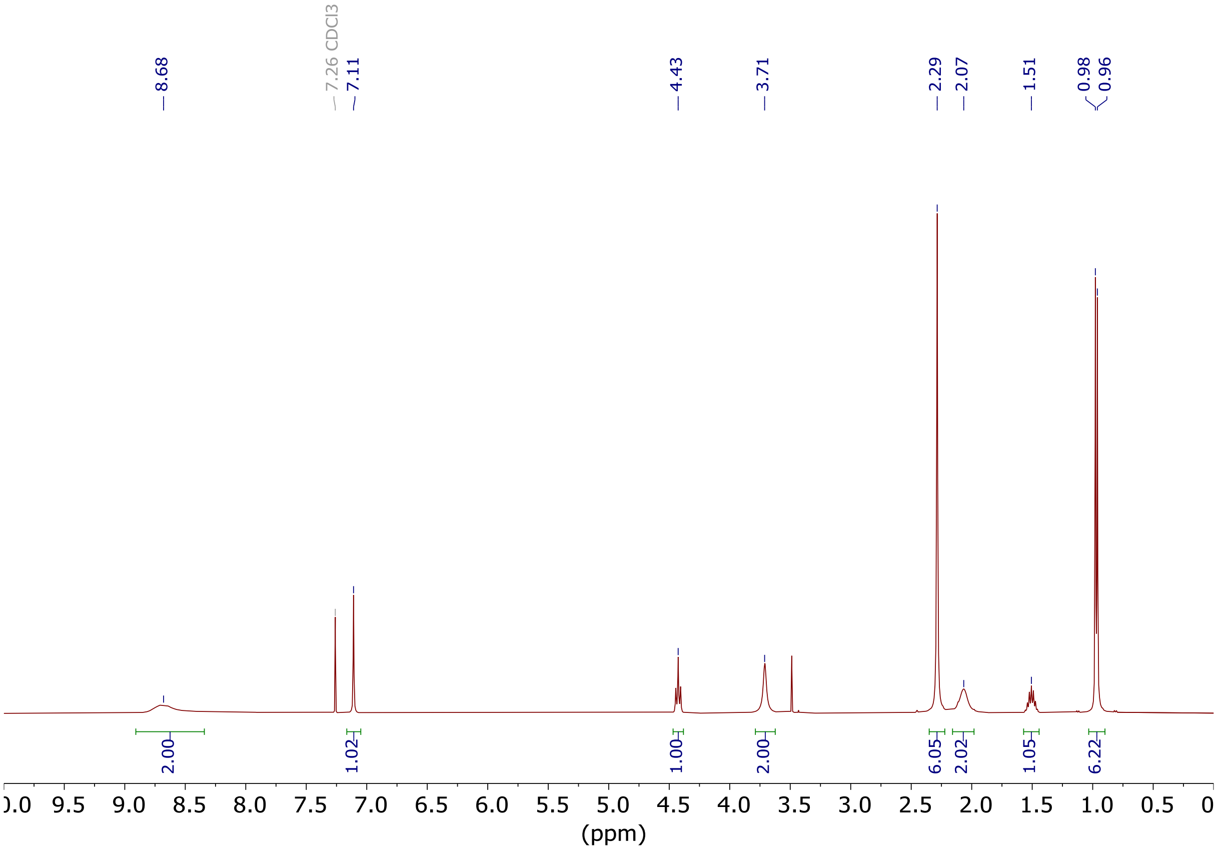

13C NMR – $CDCl_3$

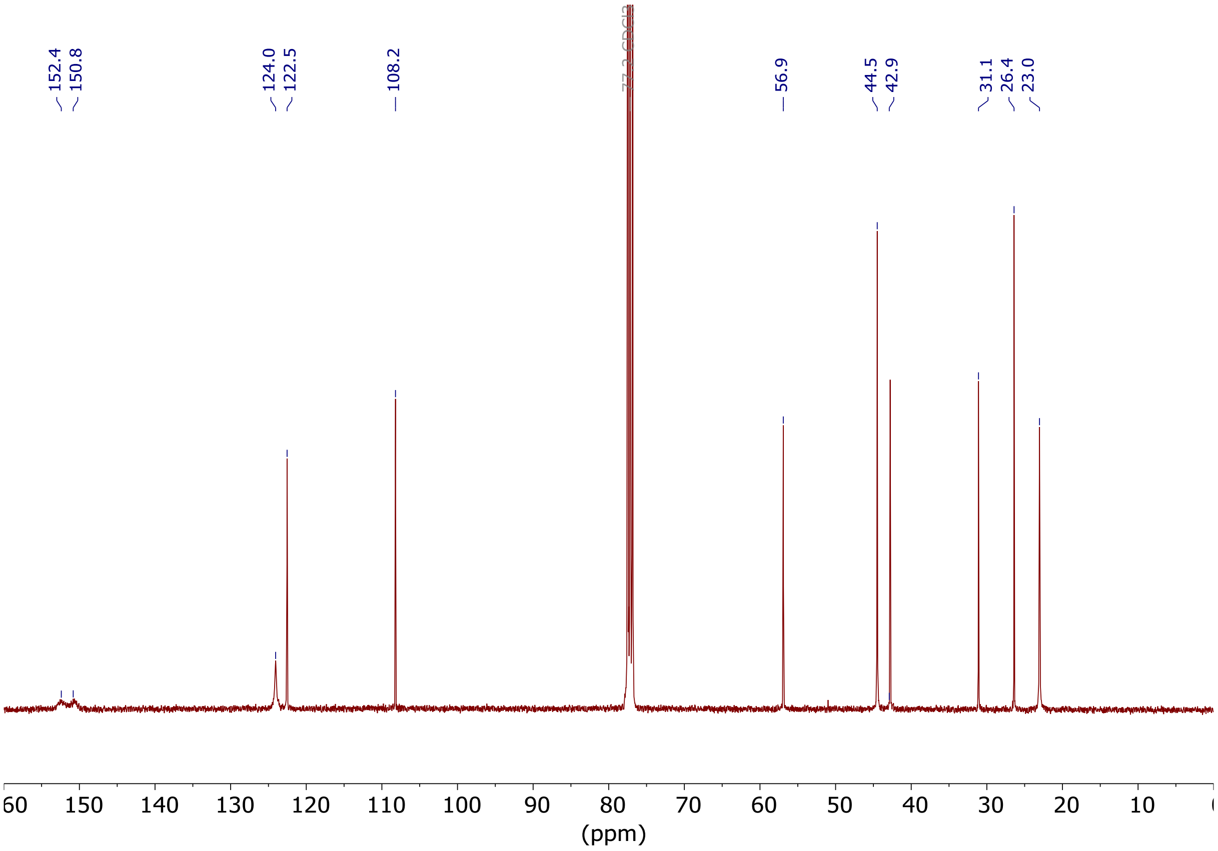

1H NMR – CCl_4

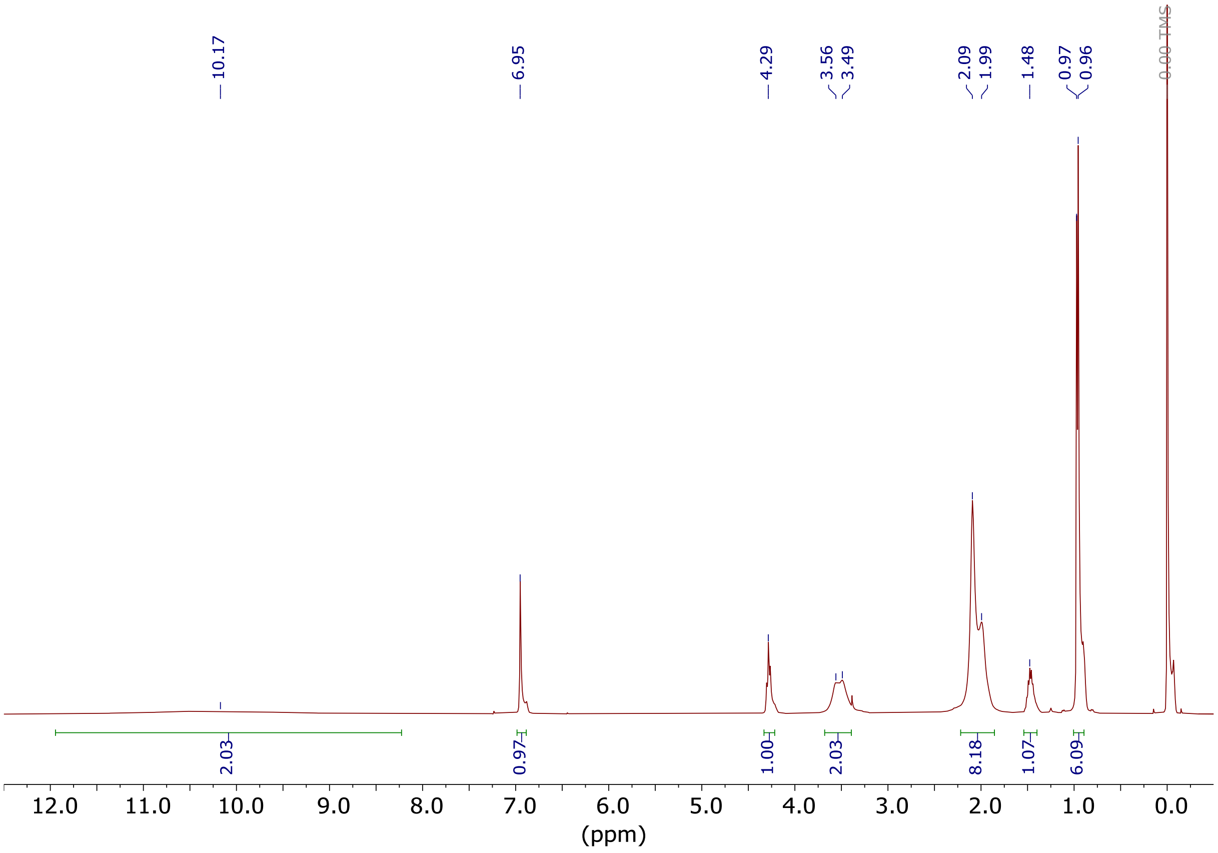

NMR Spectra of **2**

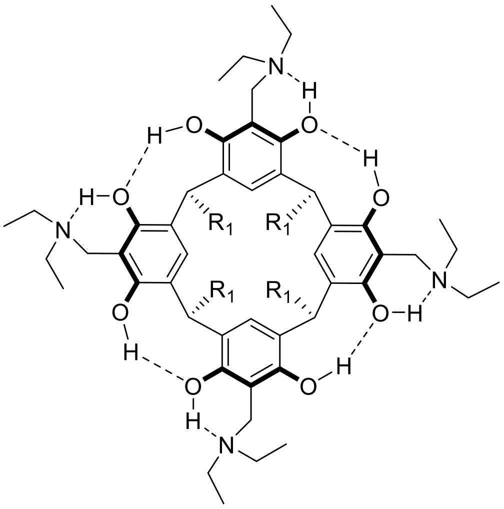

$R_1 = CH_2CH(CH_3)_2$

1H NMR – $CDCl_3$

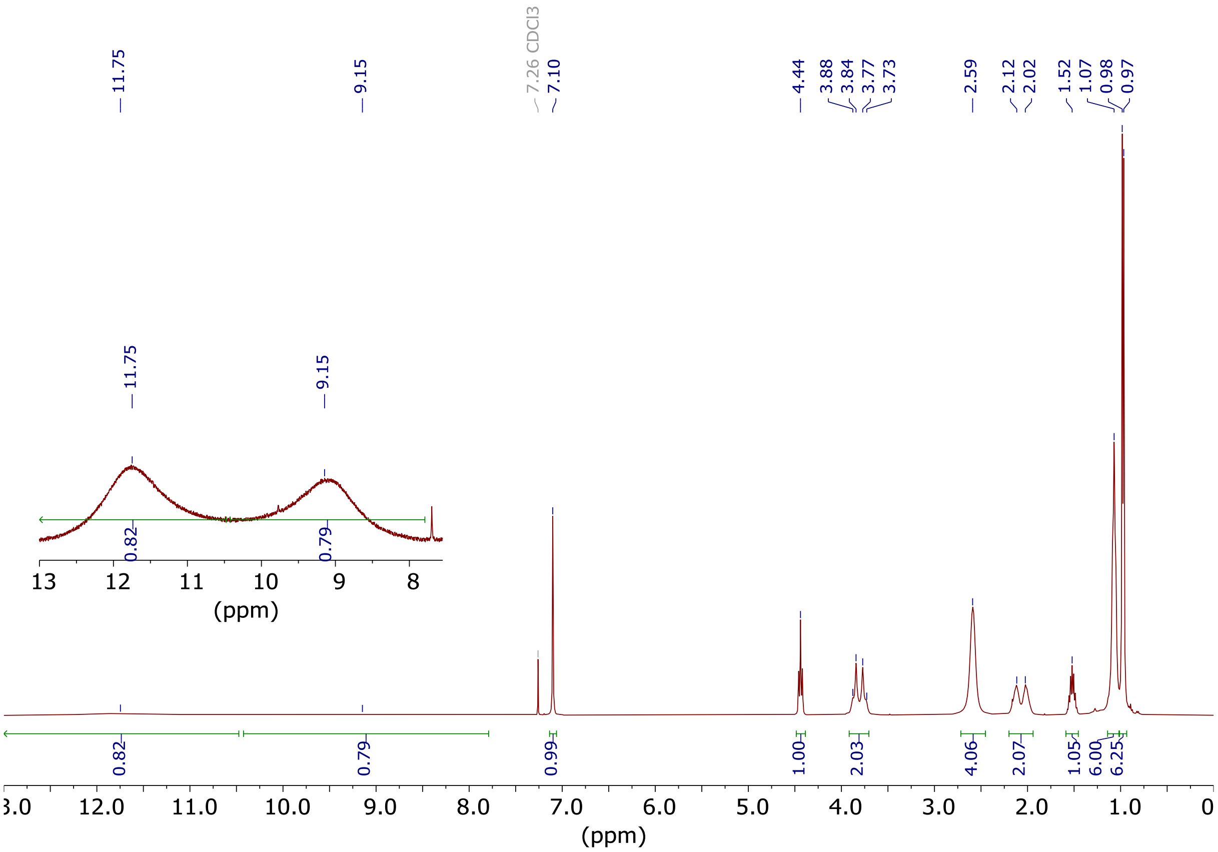

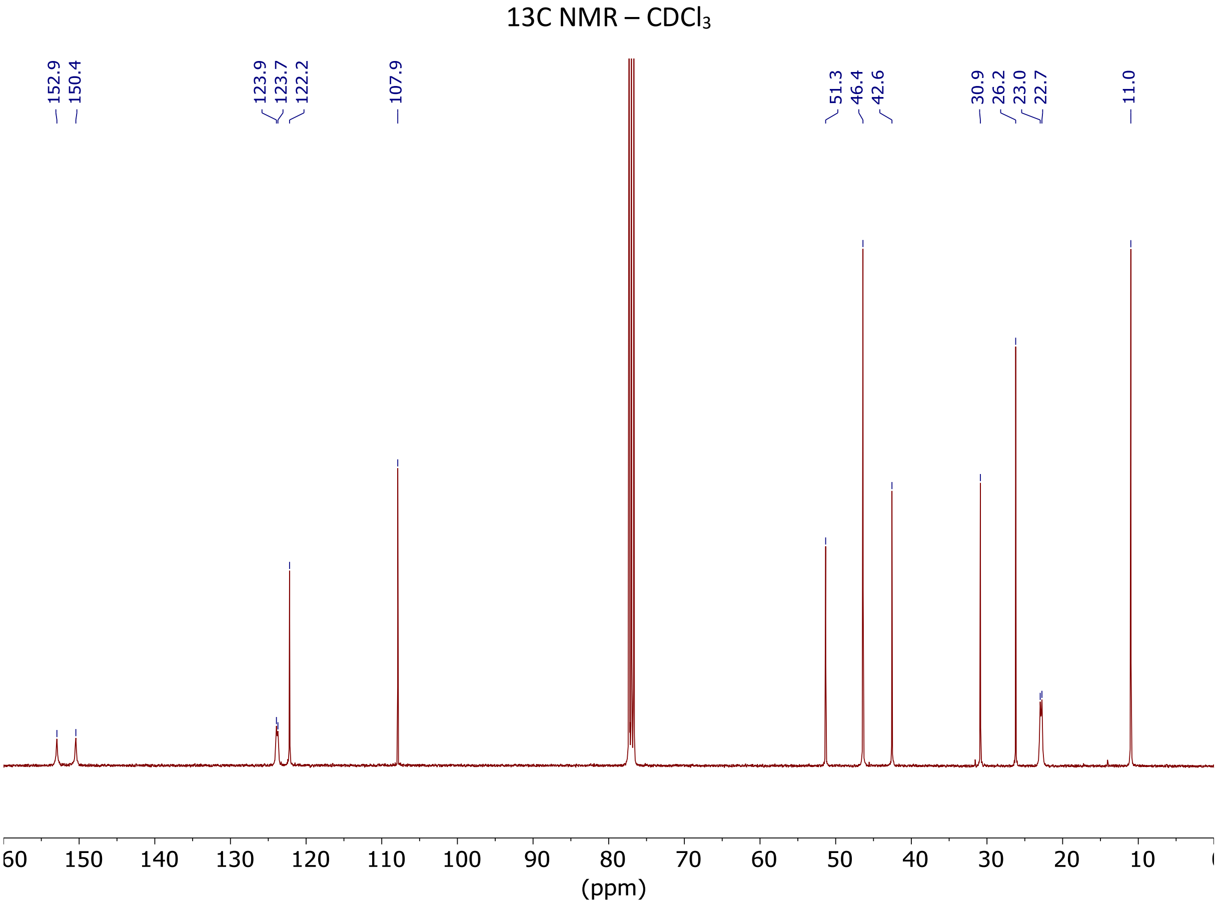
13C NMR – CDCl3
152.9
150.4
123.9
123.7
122.2
107.9
51.3
46.4
42.6
30.9
26.2
23.0
22.7
11.0
60 150 140 130 120 110 100 90 80 70 60 50 40 30 20 10
(ppm)

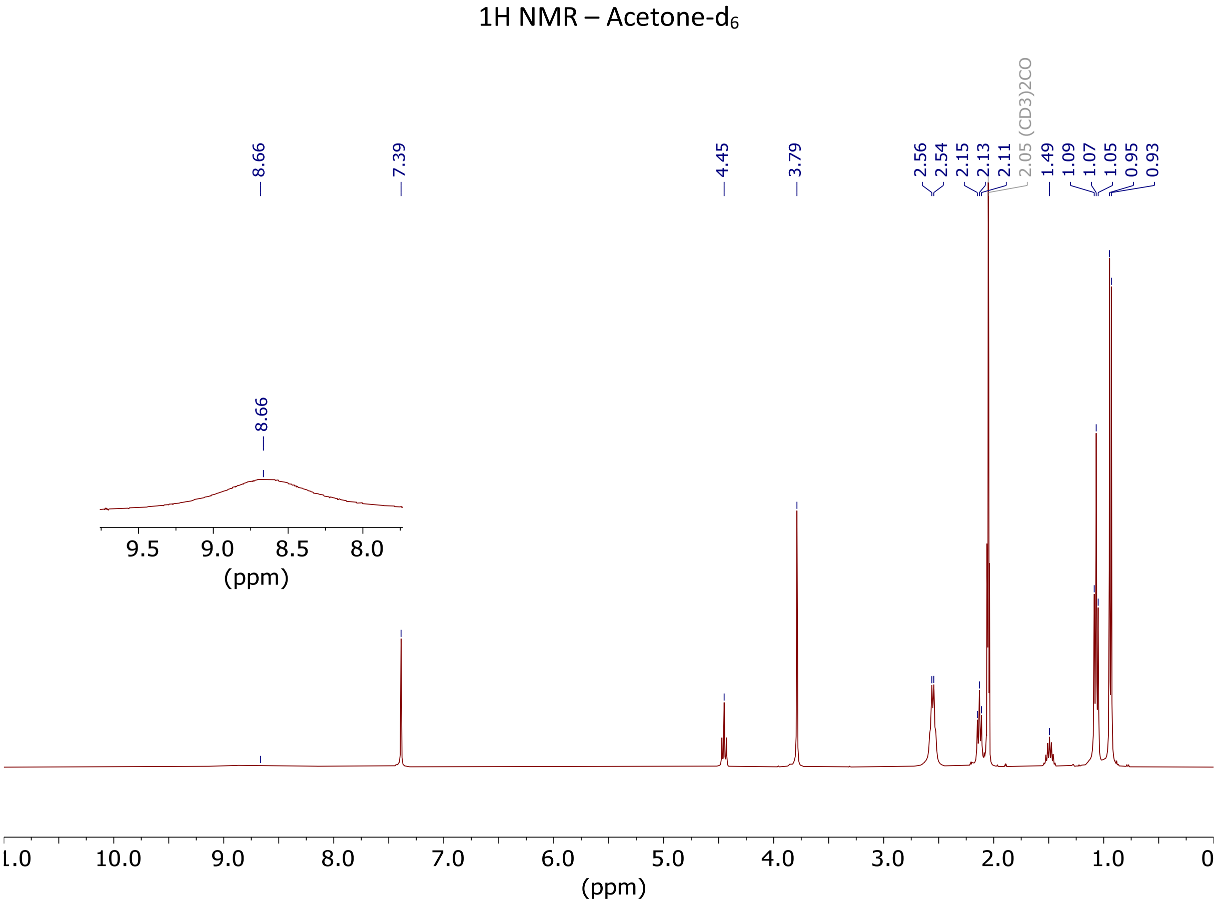
1H NMR – Acetone-d6
8.66
7.39
4.45
3.79
2.56
2.54
2.15
2.13
2.11
2.05 (CD3)2CO
1.49
1.09
1.07
1.05
0.95
0.93
8.66
9.5 9.0 8.5 8.0
(ppm)
10.0 9.0 8.0 7.0 6.0 5.0 4.0 3.0 2.0 1.0 0
(ppm)

13C NMR – Acetone-d_6

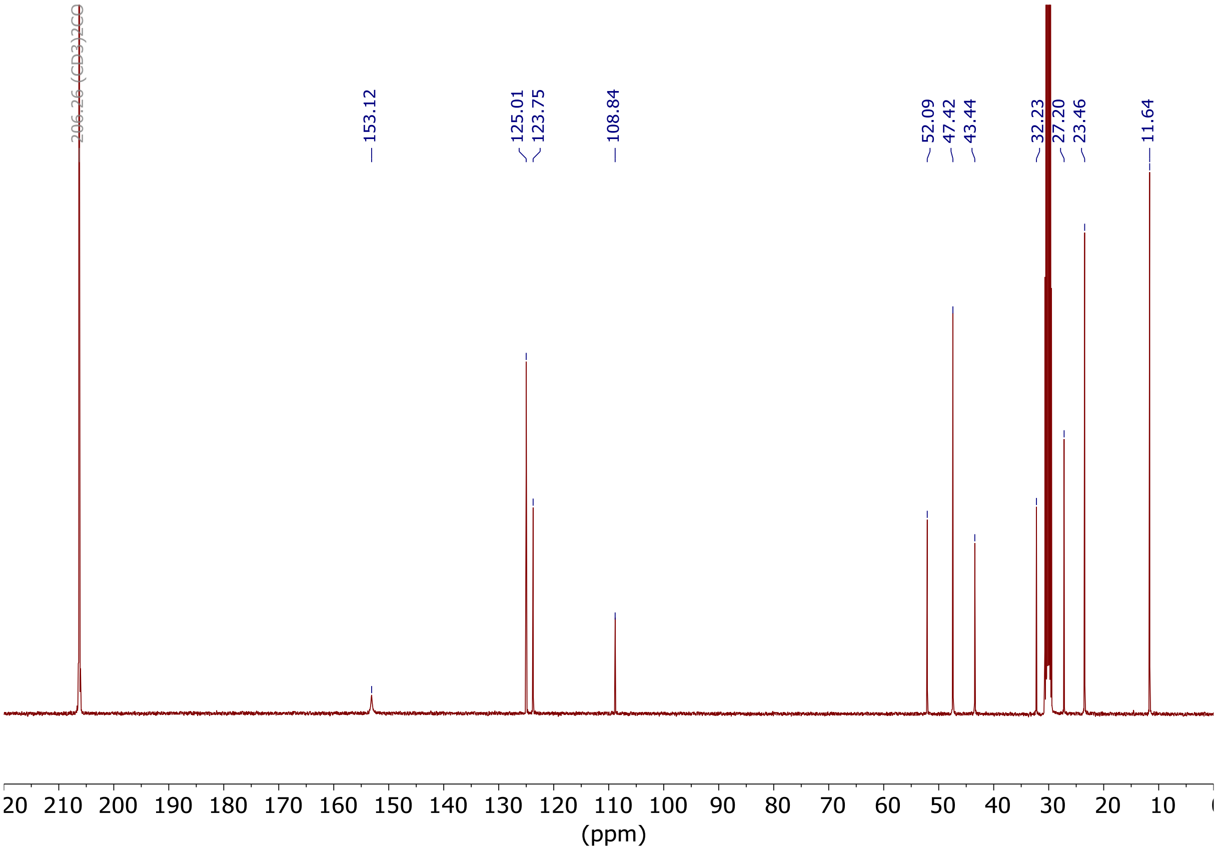

1H NMR - CCl_4

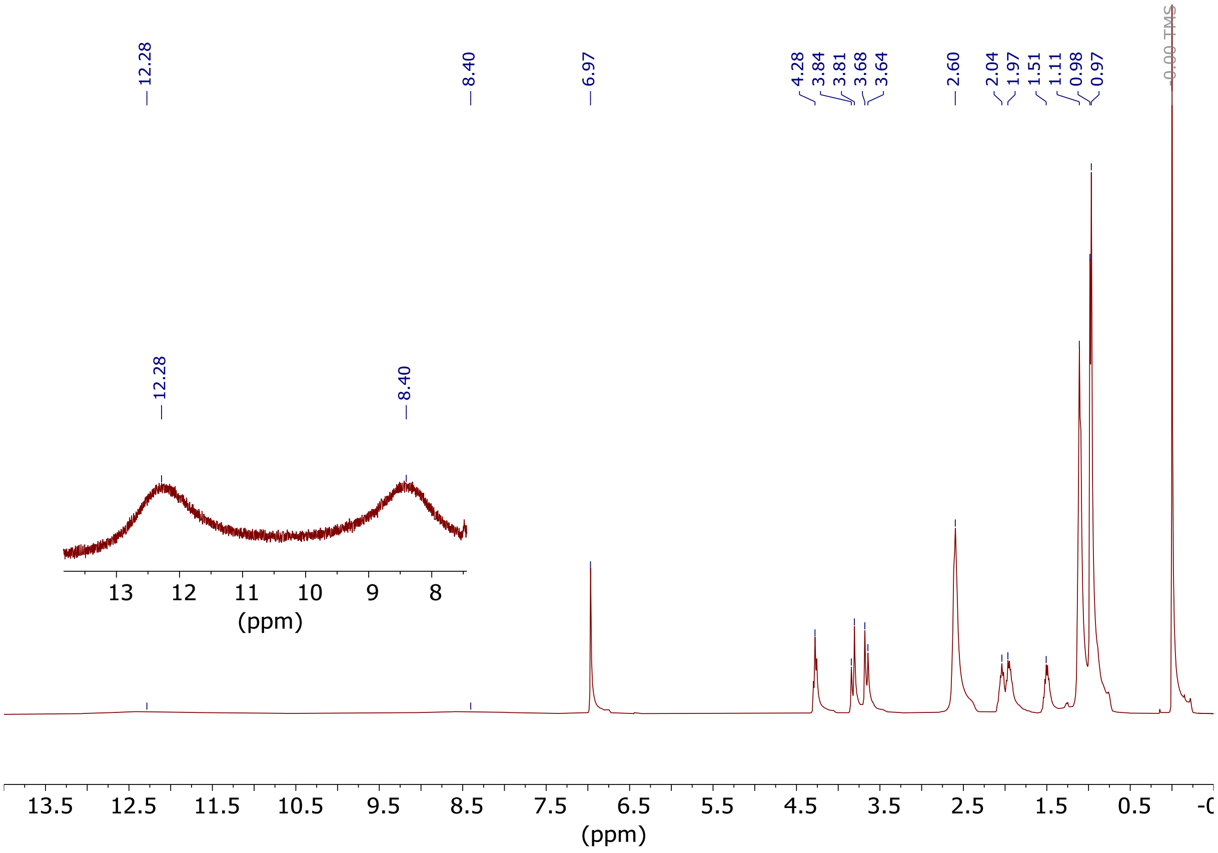

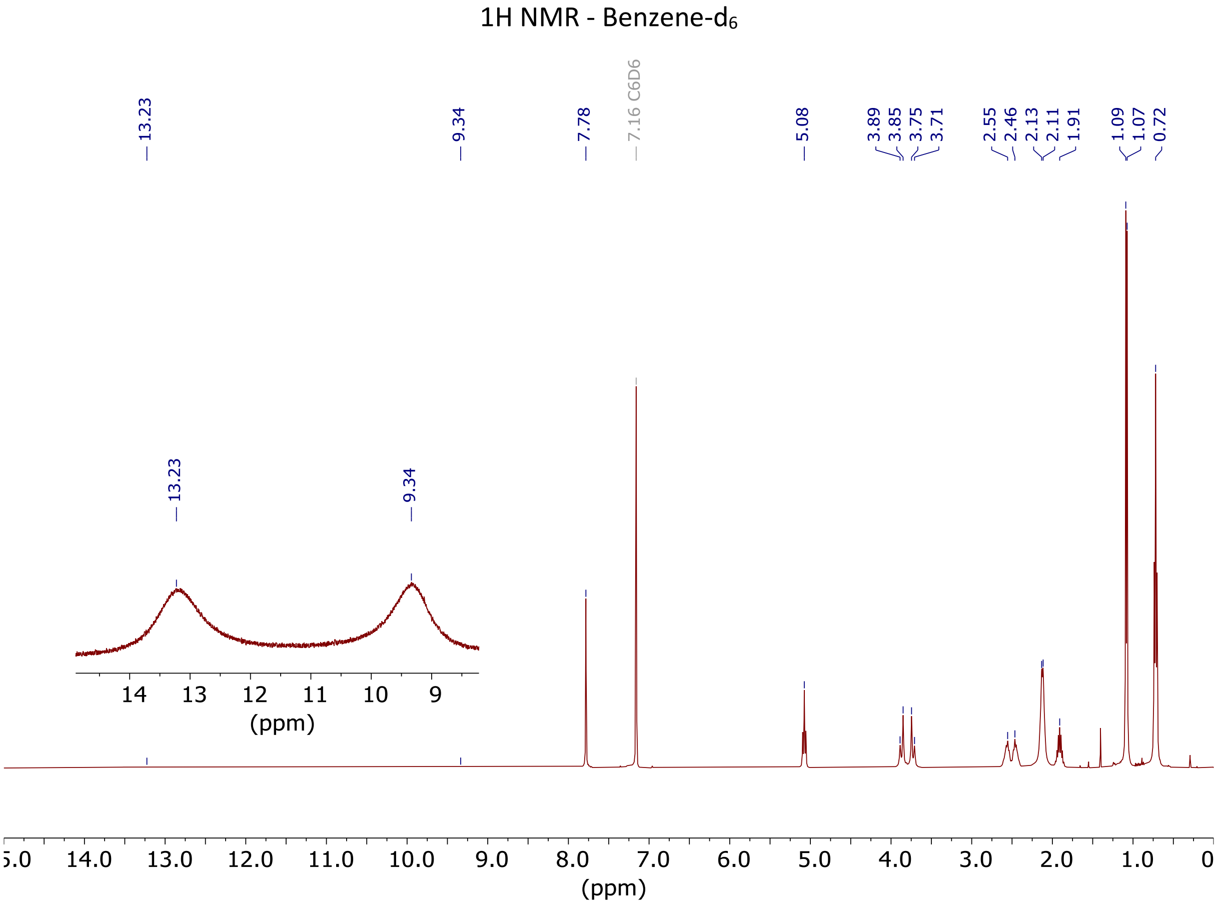
1H NMR - Benzene-d6
13.23
9.34
7.78
7.16 C6D6
5.08
3.89
3.85
3.75
3.71
2.55
2.46
2.13
2.11
1.91
1.09
1.07
0.72
14.0
13.0
12.0
11.0
10.0
9.0
8.0
7.0
6.0
5.0
4.0
3.0
2.0
1.0
0
(ppm)

NMR Spectra of **3**

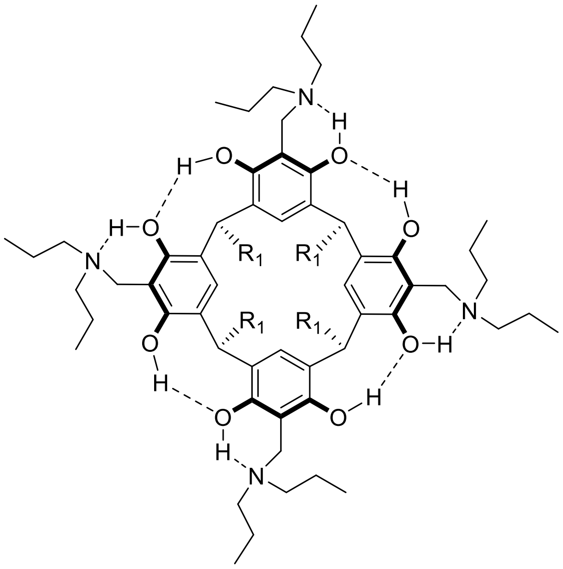

$R_1 = CH_2CH(CH_3)_2$

1H NMR – $CDCl_3$

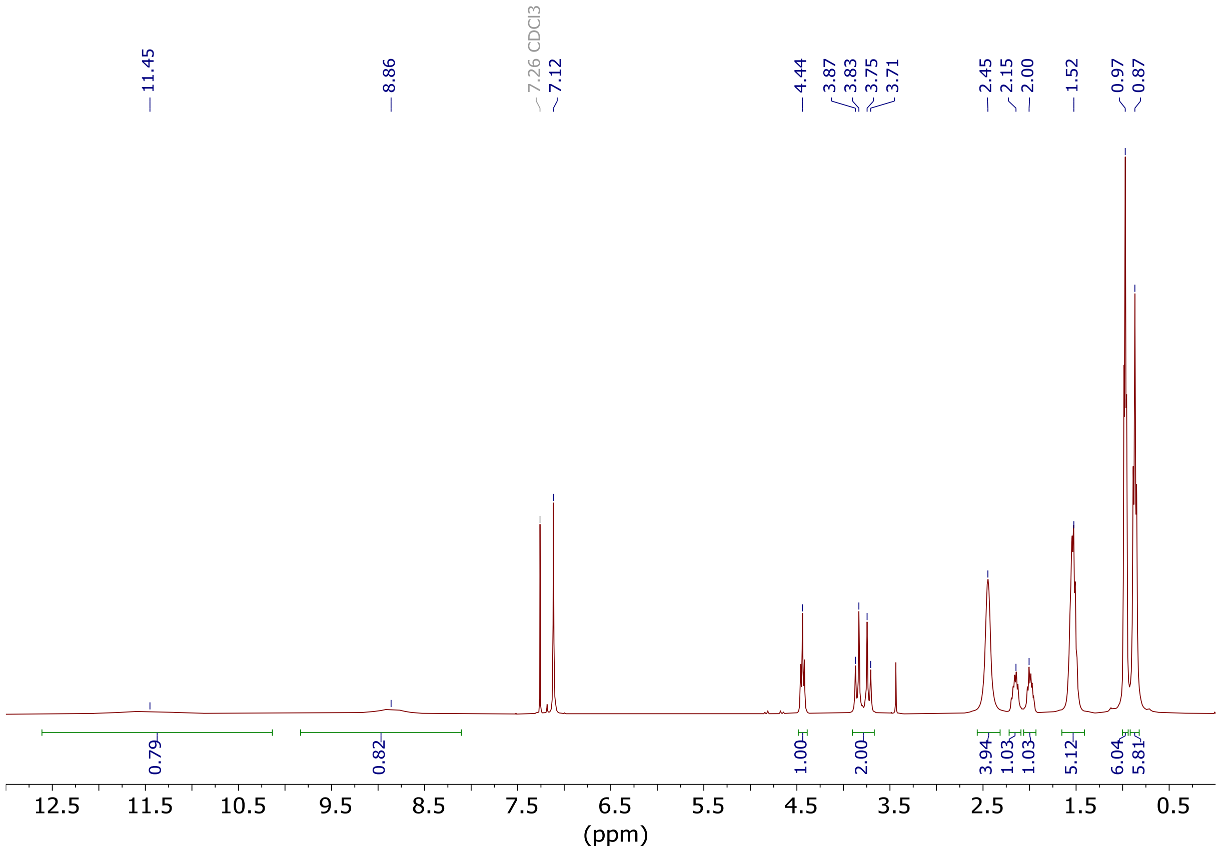

13C NMR – $CDCl_3$

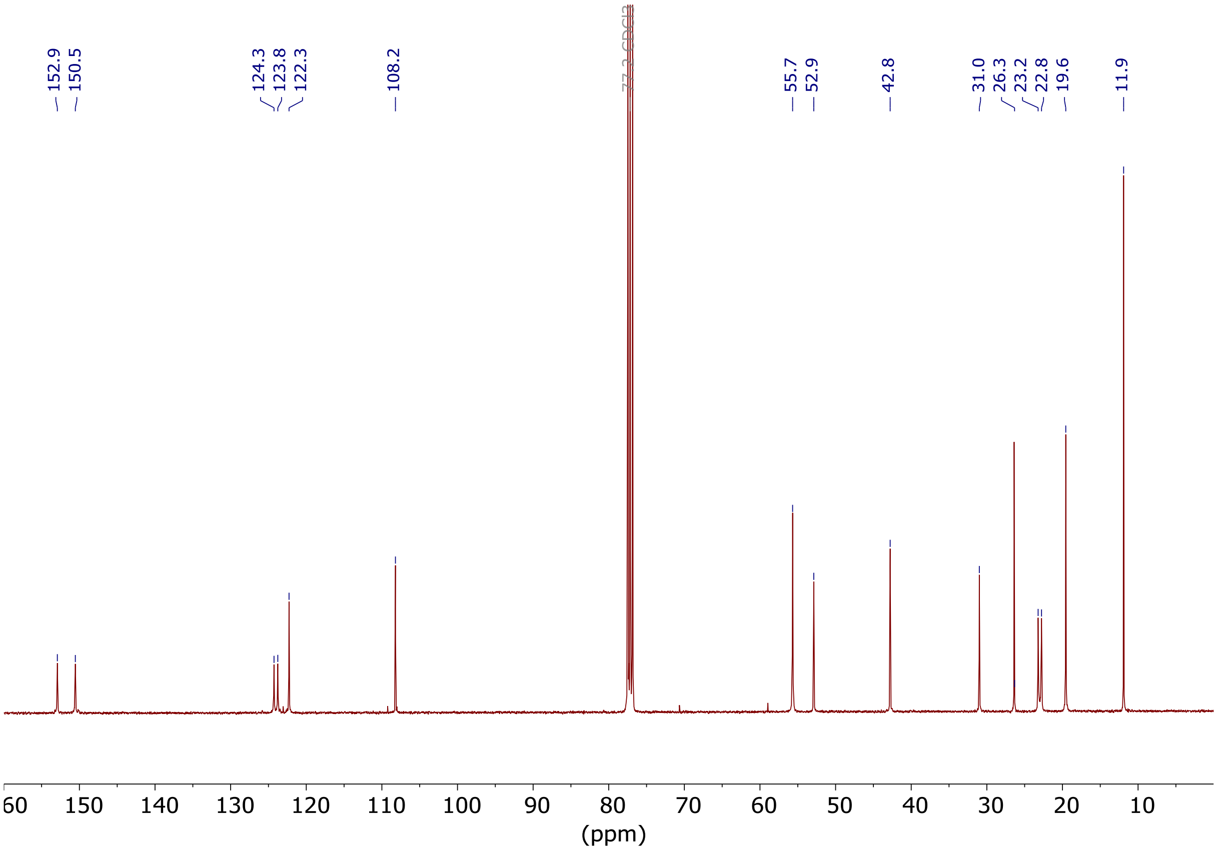

1H NMR – CCl_4

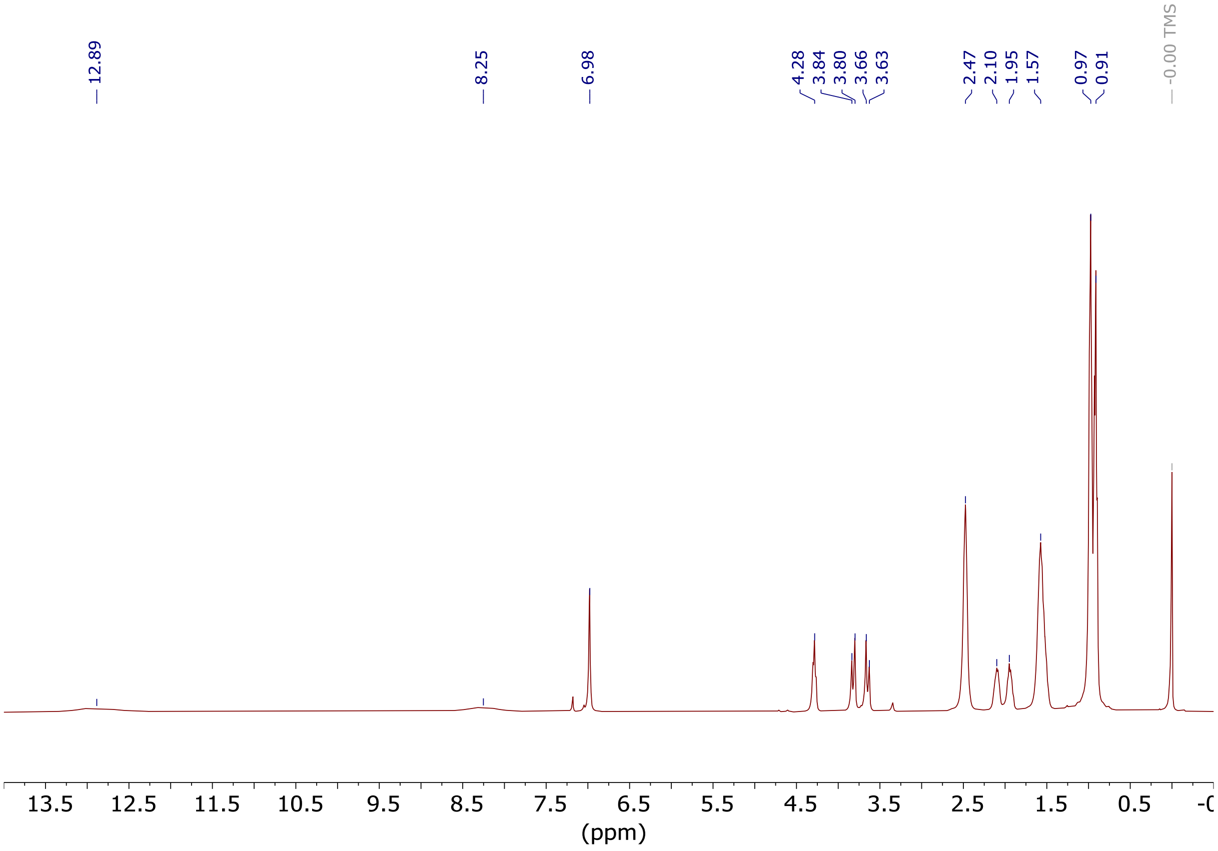

NMR Spectra of **4**

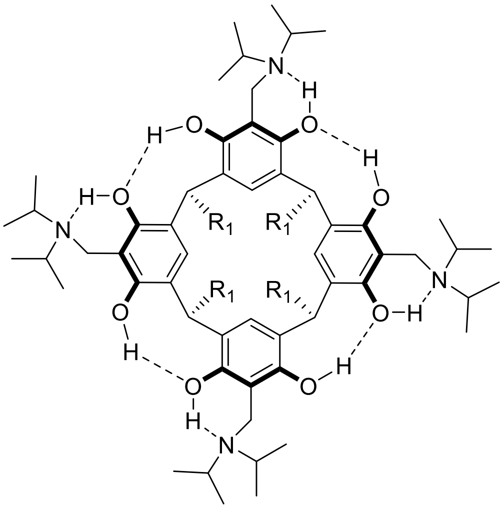

$R_1 = CH_2CH(CH_3)_2$

1H NMR – $CDCl_3$

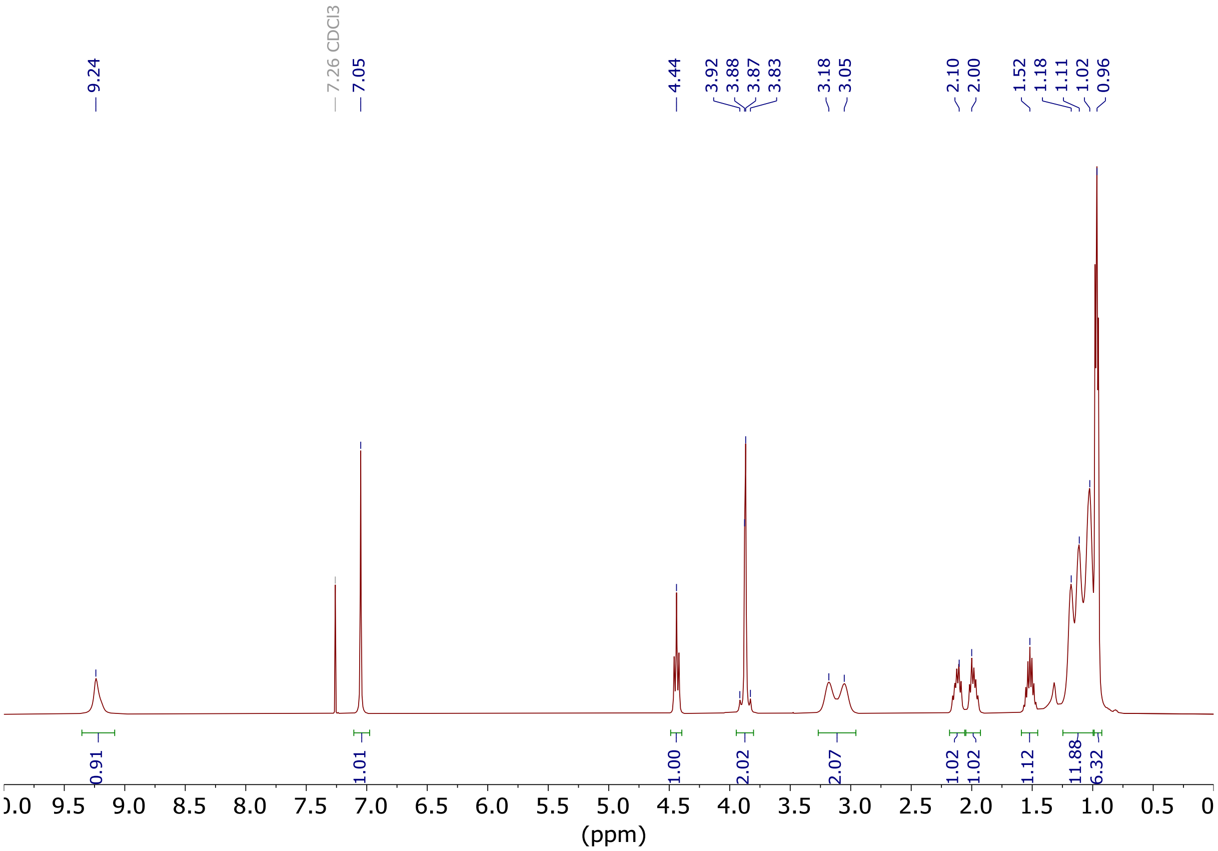

13C NMR – $CDCl_3$

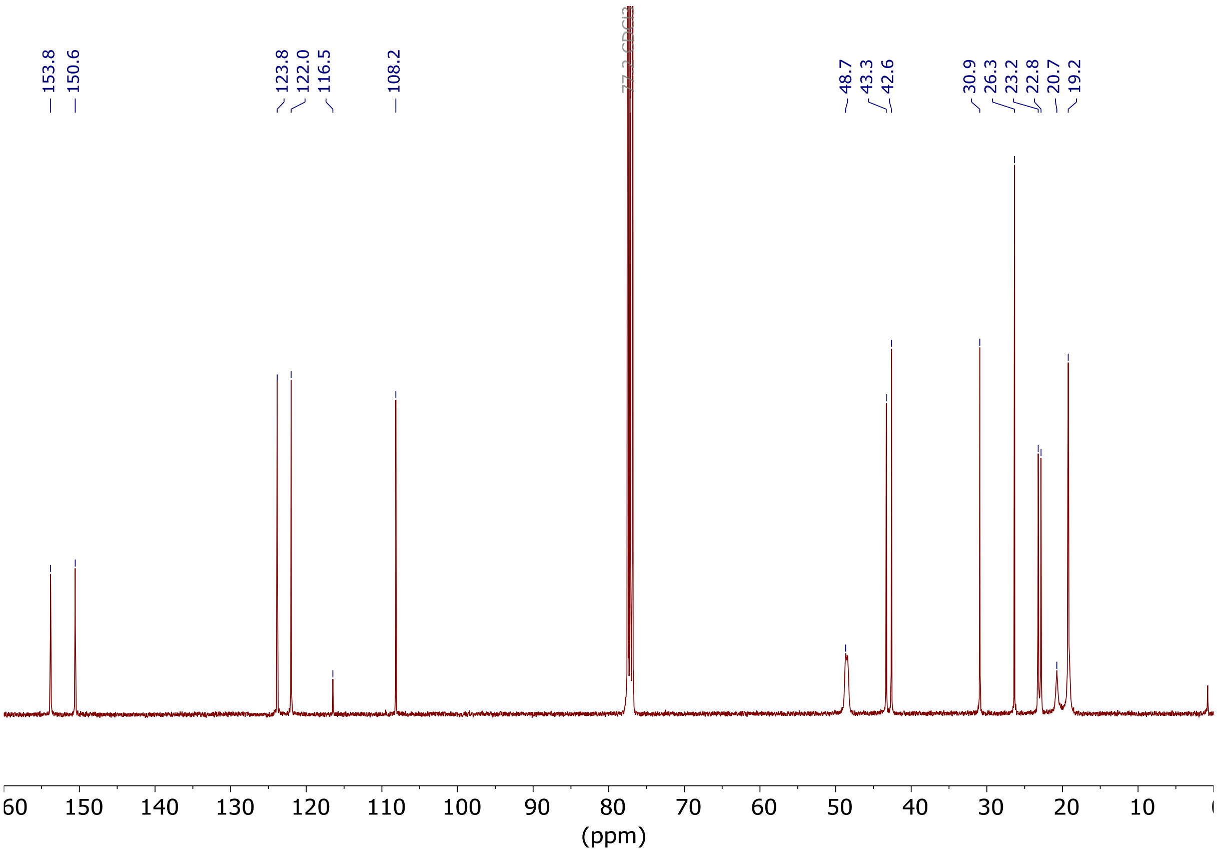

NMR Spectra of **5**

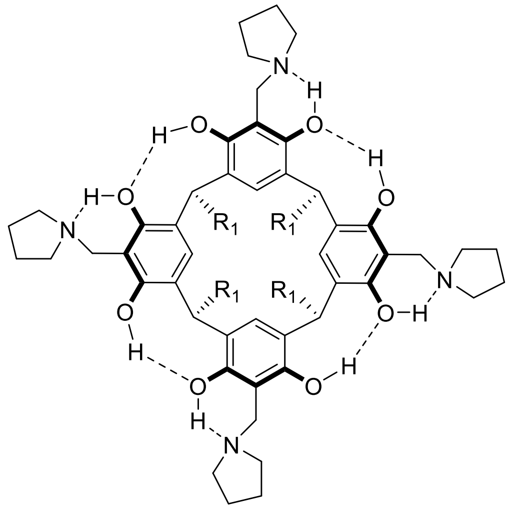

$R_1 = CH_2CH(CH_3)_2$

1HNMR – $CDCl_3$

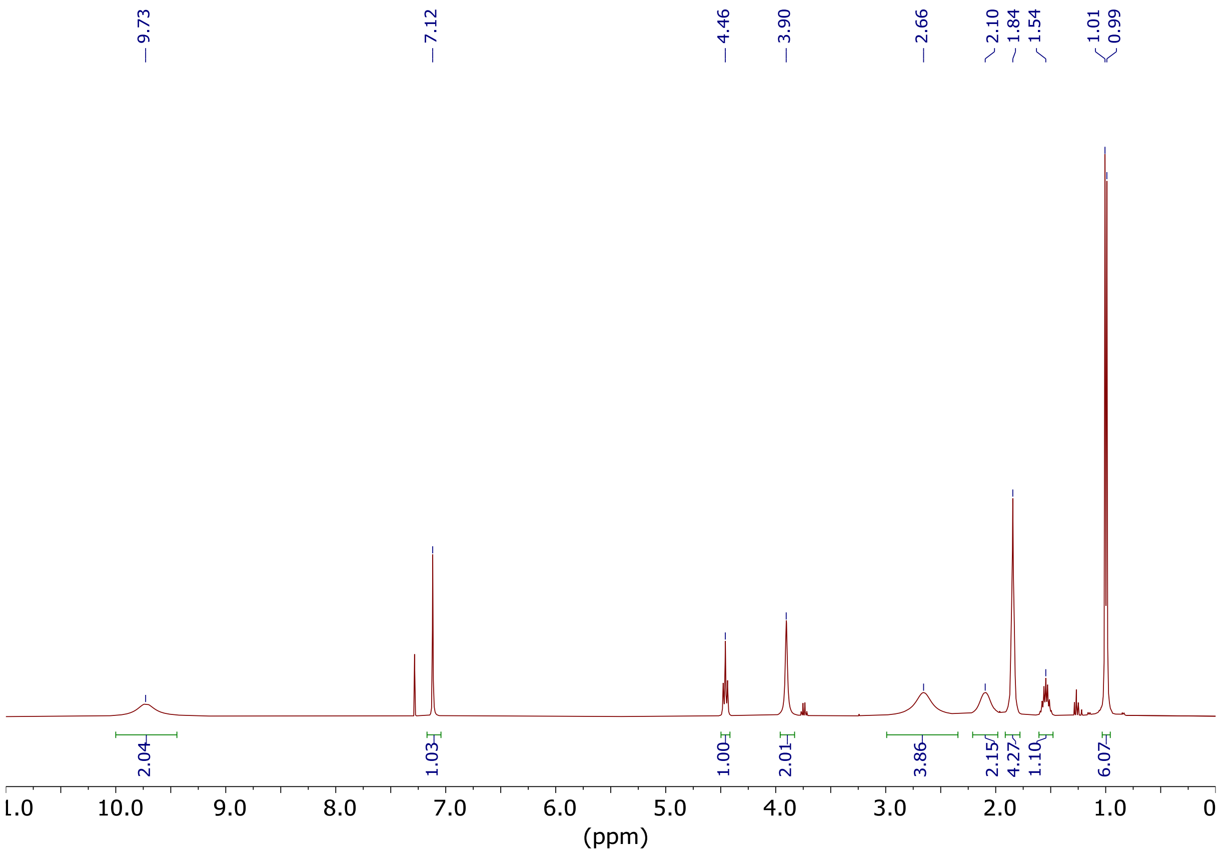

13C NMR – $CDCl_3$

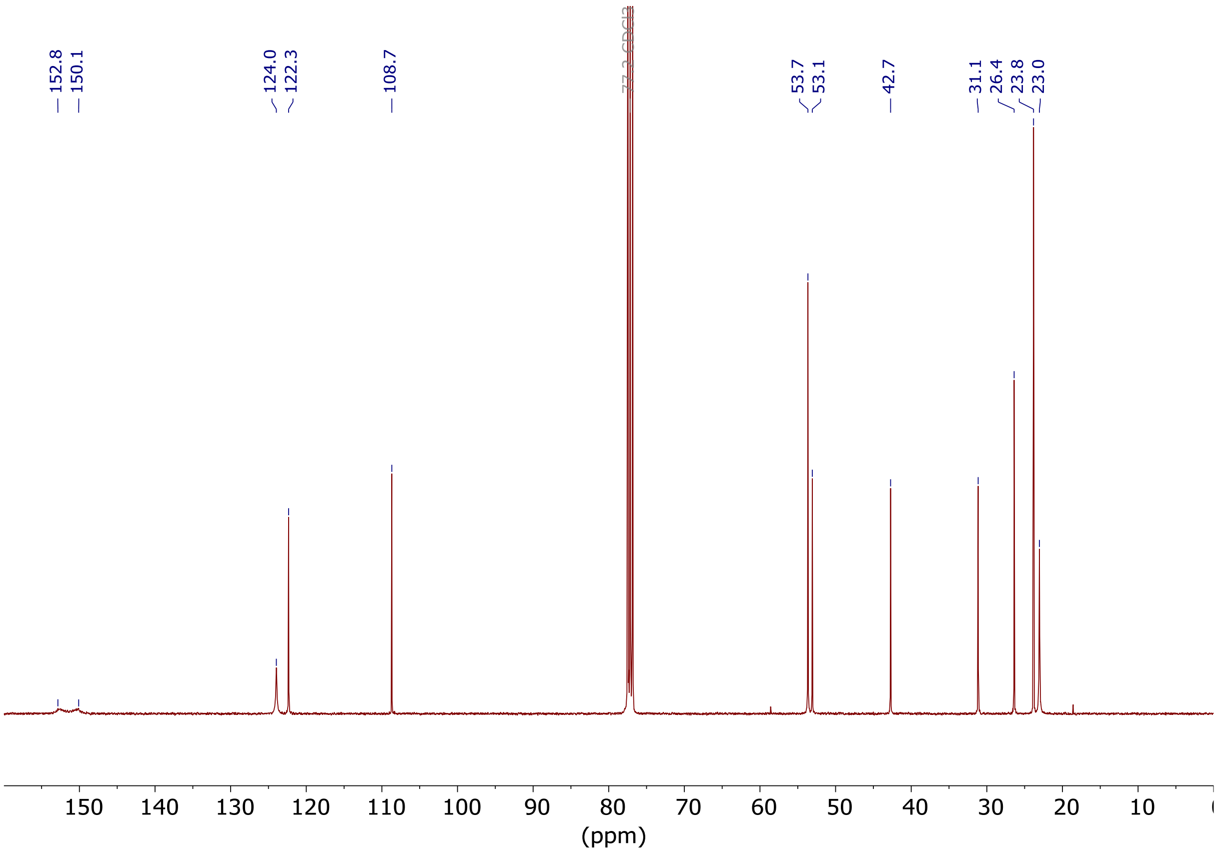

NMR Spectra of **6**

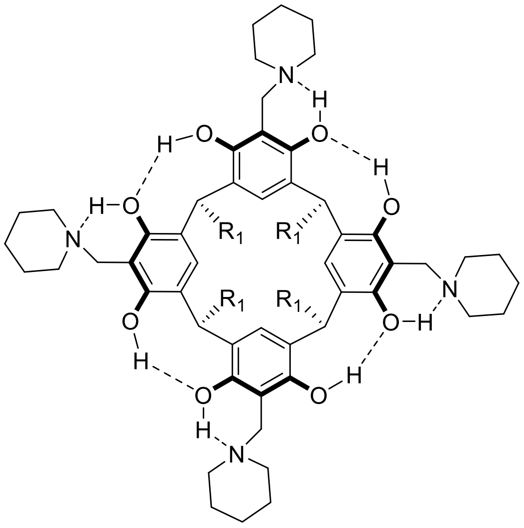

$R_1 = CH_2CH(CH_3)_2$

1H NMR – $CDCl_3$

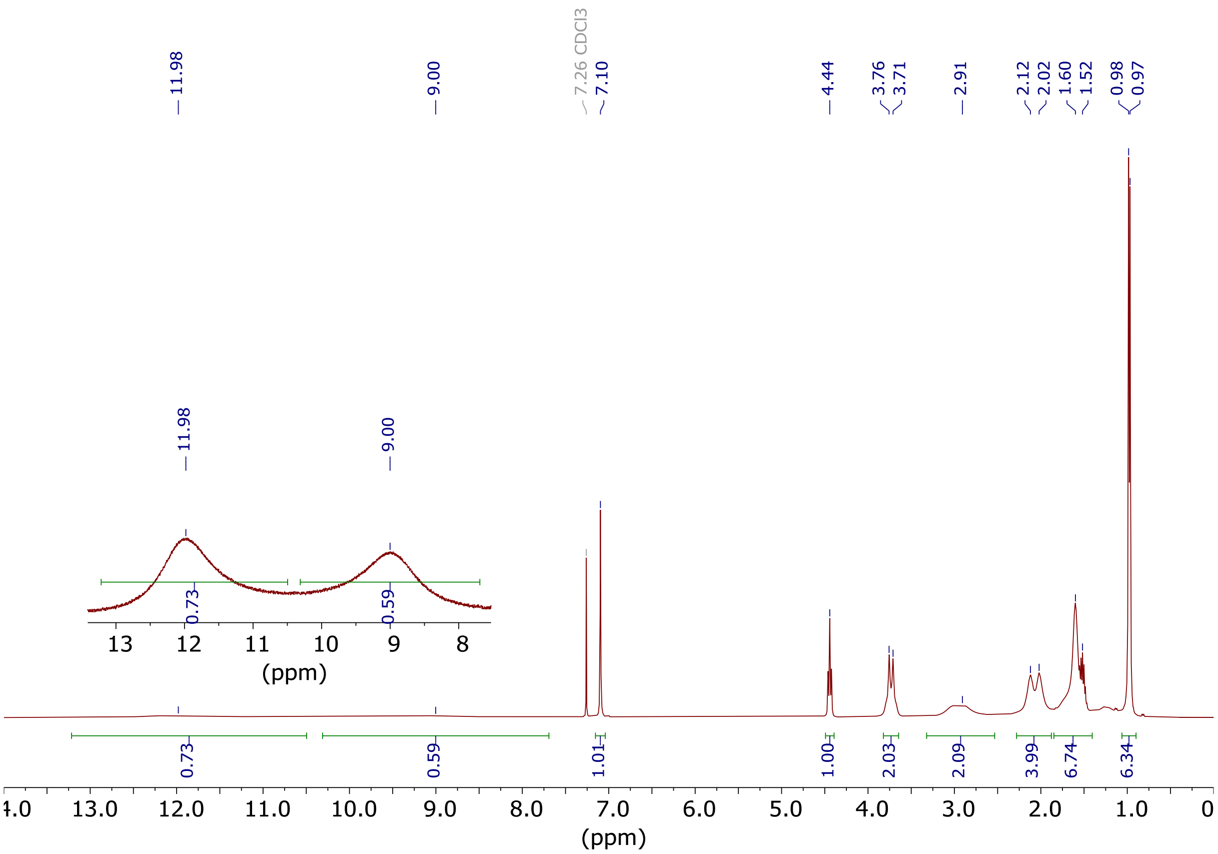

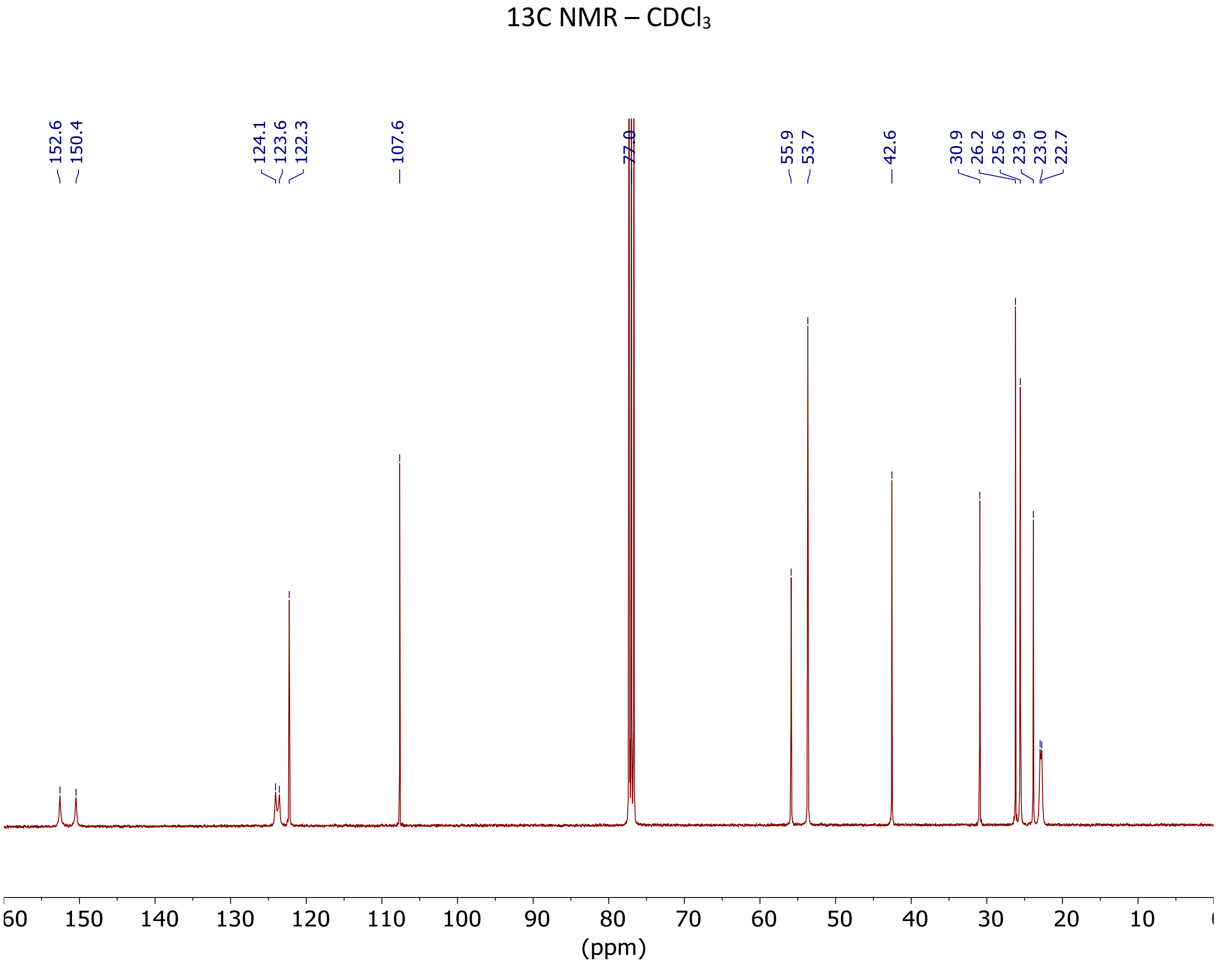
13C NMR – CDCl3
152.6
150.4
124.1
123.6
122.3
107.6
77.0
55.9
53.7
42.6
30.9
26.2
25.6
23.9
23.0
22.7
160 150 140 130 120 110 100 90 80 70 60 50 40 30 20 10 0
(ppm)

NMR Spectra of **7**

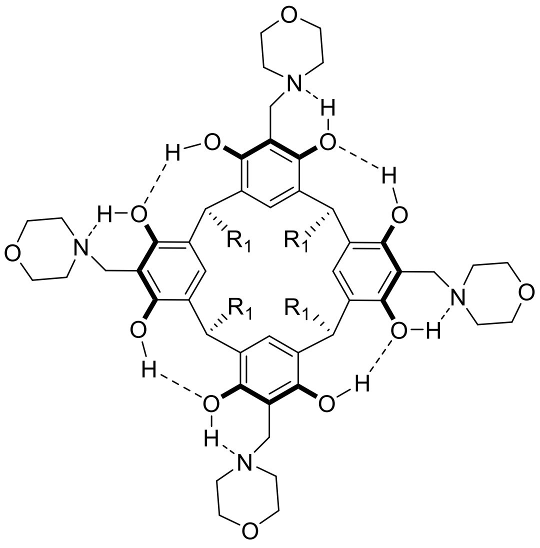

$R_1 = CH_2CH(CH_3)_2$

1H NMR – $CDCl_3$

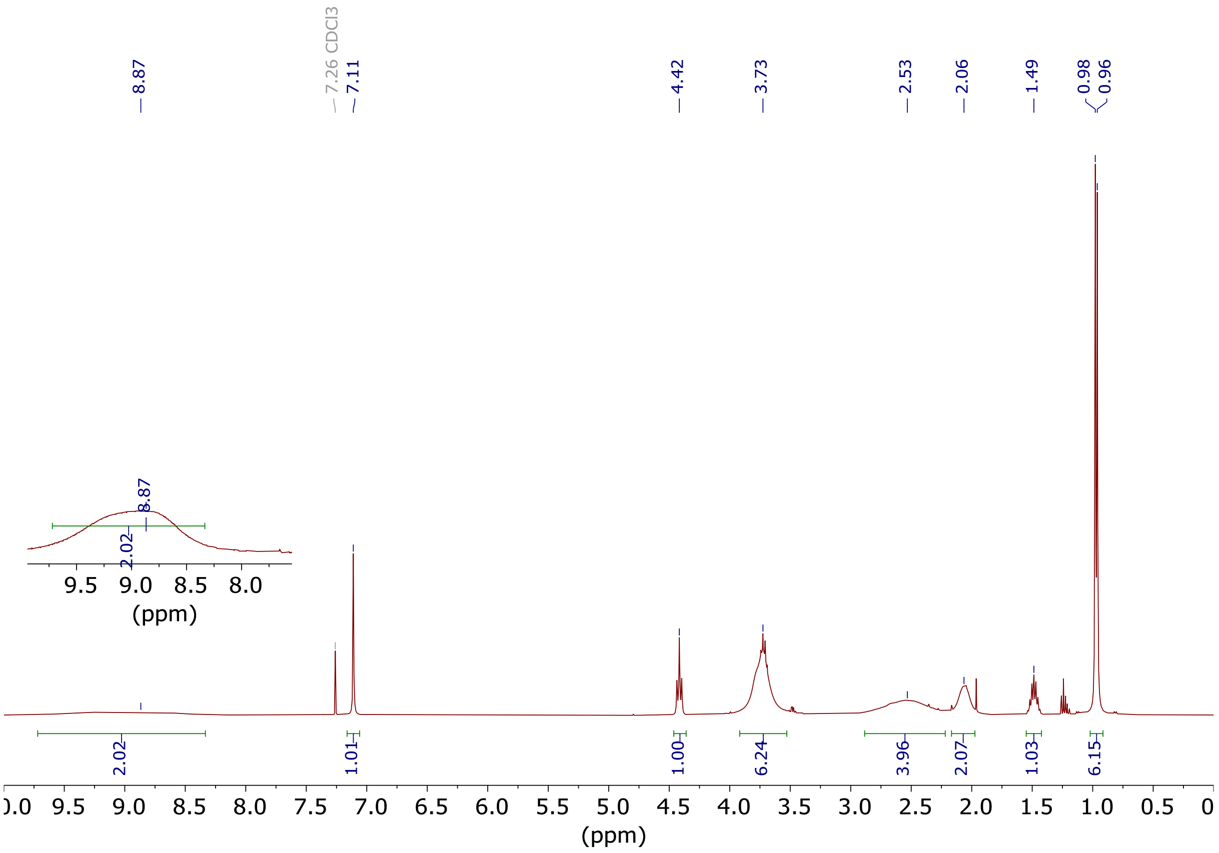

13C NMR – $CDCl_3$

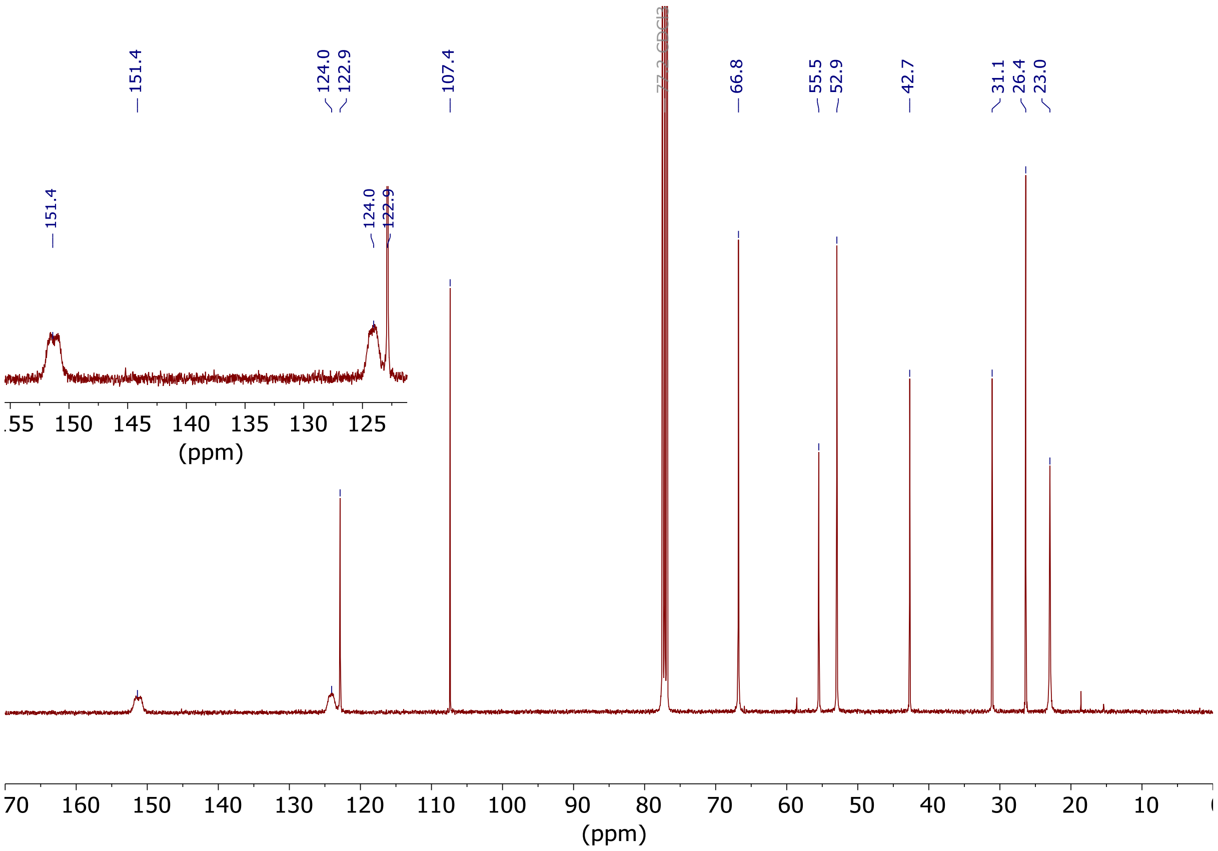

NMR Spectra of **8**

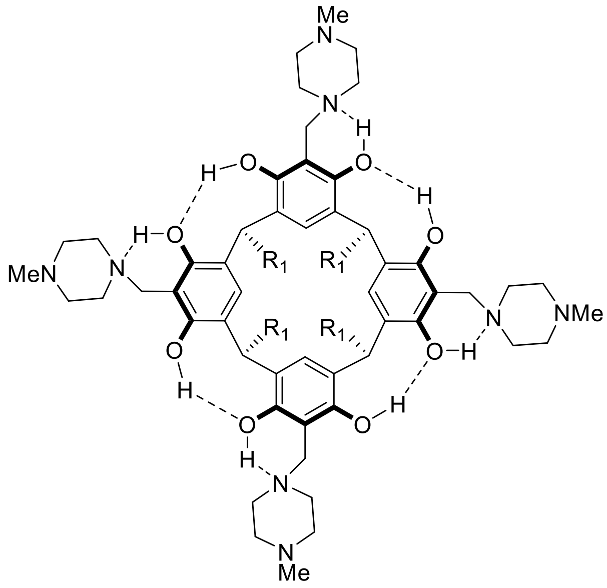

$R_1 = CH_2CH(CH_3)_2$

1H NMR – $CDCl_3$

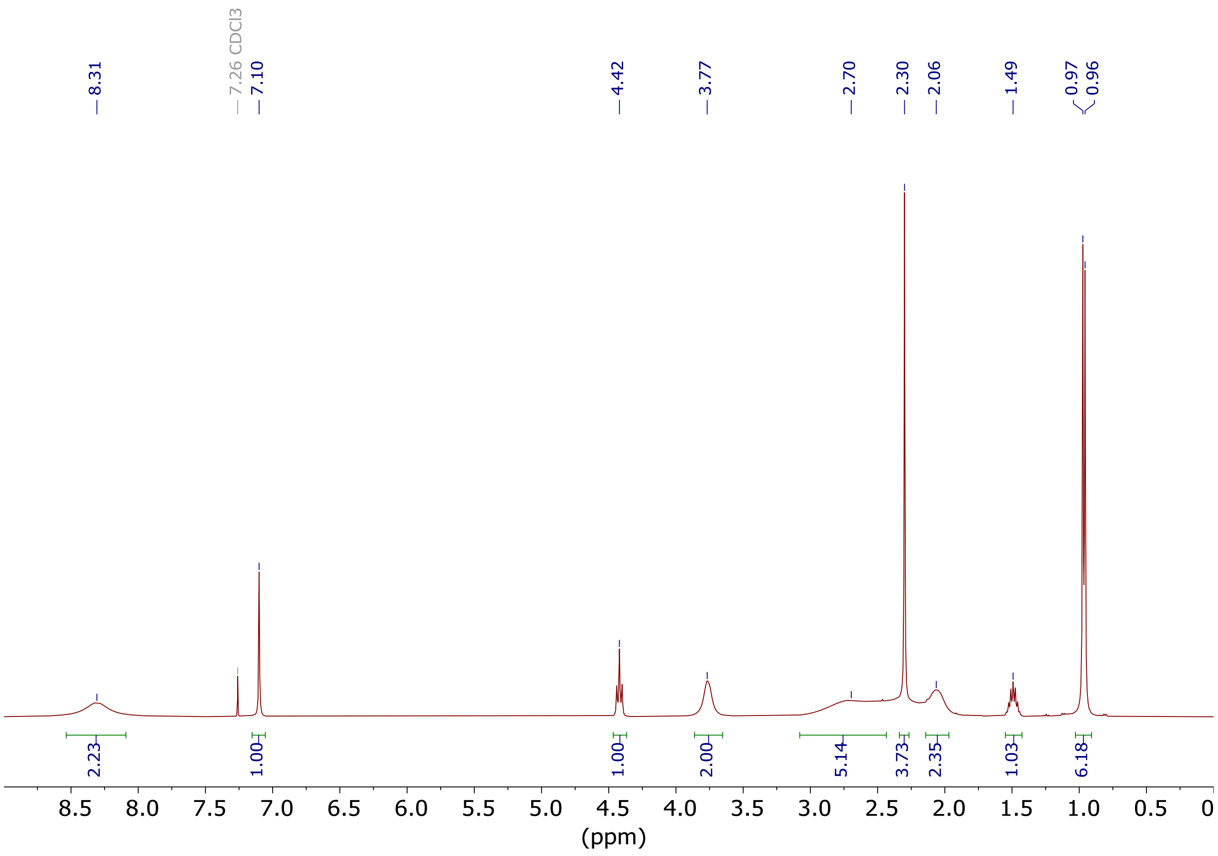

13C NMR – $CDCl_3$

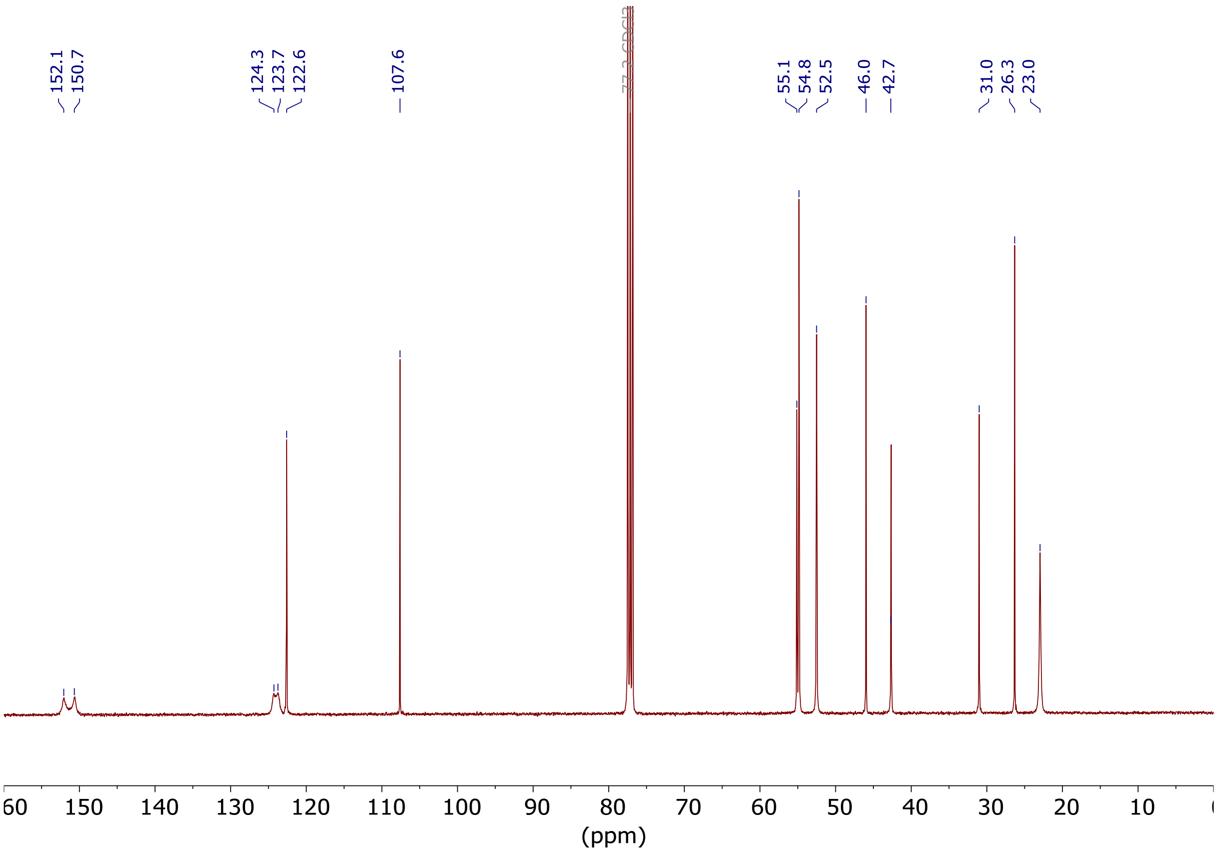

2. The xyz coordinates of the optimized structures in $CHCl_3$

Substrate (S)	Transition state (TS)	Intermediate state (IS)
	**1**	
C -0.76121095295846 -2.12604270645567 -2.47339497835997	C -0.687309000 -2.300332000 -2.350802000	C 0.06314321156344 -2.22965815330407 -2.17162403477232
C 0.28542846956428 -2.40027593326219 -3.35565007719029	C 0.316923000 -2.642008000 -3.258021000	C 1.23166455134260 -2.48118673330350 -2.90807489564649
C 0.79252595267197 -1.42292151335870 -4.21942955449393	C 0.808097000 -1.739564000 -4.200837000	C 1.73869482607263 -1.53509709515084 -3.80673040725766
C 0.22159686143467 -0.14584754520107 -4.20528266873154	C 0.260761000 -0.447898000 -4.227967000	C 1.07933087101040 -0.30573679981354 -3.94200052704789
C -0.85113667761364 0.15314176650795 -3.35465421833207	C -0.776500000 -0.078883000 -3.364463000	C -0.08804138404623 -0.02578696187373 -3.22176649752056
C -1.28735286148905 -0.84359210601879 -2.49049282769327	C -1.199399000 -1.015216000 -2.430970000	C -0.54453797568164 -0.99595962429444 -2.34196090573154
O 0.83389900816375 -3.65399448743728 -3.35777207436174	O 0.805518000 -3.931075000 -3.173521000	O 1.91375861798578 -3.66096187453379 -2.81108795375665
O 0.75760836272843 0.77476866038136 -5.06235704580386	O 0.780449000 0.411047000 -5.150288000	O 1.63375235199282 0.58569937727774 -4.81459792974929
H -2.09535408030293 -0.61004123895903 -1.81250532950008	H -1.988179000 -0.733691000 -1.749243000	H -1.44586297673997 -0.78768909694561 -1.78579007683458
C -1.52447422156008 1.51252425879985 -3.36944597796770	C -1.450808000 1.276803000 -3.465966000	C -0.83979239784546 1.27624164205796 -3.40458869072077
C -0.93874009968065 2.42694110778136 -2.31479348196979	C -0.862244000 2.270249000 -2.484453000	C -0.34101290913596 2.33853305798319 -2.44798302083849
C 0.13815878077836 3.25535019487478 -2.63536370129647	C 0.182717000 3.113275000 -2.863093000	C 0.58630523344472 3.28539080129228 -2.88337652511801
C 0.73279060760508 4.08810589647668 -1.68082270567163	C 0.770426000 4.019746000 -1.981948000	C 1.07540789591041 4.28731021735089 -2.03084122836546
C 0.22371368677321 4.09714692617342 -0.37823749926177	C 0.276099000 4.079813000 -0.671369000	C 0.59256670276663 4.32692539070183 -0.72322859845770
C -0.88049361997953 3.30442863367036 -0.03082254358999	C -0.800185000 3.282456000 -0.259835000	C -0.32706876608490 3.39128659470860 -0.24406796823551
C -1.40818263081743 2.47070051668681 -1.01000506514910	C -1.320269000 2.382405000 -1.180209000	C -0.75550998465216 2.40770733388490 -1.12517001360062
O 0.62731350544780 3.24100372497516 -3.91299251181360	O 0.613197000 3.008467000 -4.173292000	O 1.02792226842463 3.22617665029173 -4.17345366963325
H -2.23586085754506 1.83104801985003 -0.74275019351033	H -2.134508000 1.746399000 -0.869370000	H -1.44892800653611 1.66253150042824 -0.76308123152661
C -1.46417869813426 3.34775895889502 1.36875219499172	C -1.381350000 3.412180000 1.135784000	C -0.81979328763933 3.45330156489274 1.18908625091530
C -0.80431793840445 2.32561837470013 2.26889329711618	C -0.716288000 2.460045000 2.108720000	C -0.07170961201320 2.48491098052714 2.08682871386831
C 0.25632923982131 2.69138389934962 3.09734824215202	C 0.311103000 2.886459000 2.949540000	C 1.07370270409832 2.88137983081355 2.78905157693666
C 0.89249543539203 1.76132236473740 3.92793652170760	C 0.930640000 2.031329000 3.860949000	C 1.73219096766738 2.00974116151379 3.66520467421619
C 0.43690840285329 0.43926614399733 3.93576912878810	C 0.494617000 0.699197000 3.920450000	C 1.24551945099853 0.70602113441255 3.80920754034414
C -0.65022823422979 0.04790268356672 3.14183885803713	C -0.560386000 0.242501000 3.121569000	C 0.09863916360259 0.27628551742522 3.12731670789590
C -1.21473351456254 1.00220282762828 2.30653651540803	C -1.114763000 1.137605000 2.217840000	C -0.51077910193806 1.18079385380997 2.26971537118477
O 0.68961899013397 3.98938921385982 3.07933042414300	O 0.686926000 4.210730000 2.833648000	O 1.60577660673551 4.13375815478906 2.66798712661734
O 1.09613278357308 -0.43159829210864 4.75879984814598	O 1.138064000 -0.110630000 4.809179000	O 1.92804430259938 -0.11273380460244 4.66561570868785
H -2.04055990234573 0.70586565585632 1.67537319342255	H -1.924728000 0.797423000 1.588793000	H -1.40275120417570 0.86707769939787 1.74789616009346
C -1.20196862641871 -1.36315743031539 3.19197946909318	C -1.109088000 -1.165101000 3.259502000	C -0.47006651917362 -1.11242027419652 3.34874066768464
C -0.59718934001961 -2.22403777451074 2.10488275593755	C -0.503515000 -2.102688000 2.235900000	C 0.09307127145115 -2.11771832656389 2.36326828492463
C 0.55420443374273 -2.96886551086604 2.36680314198661	C 0.627258000 -2.860971000 2.545679000	C 1.13899165118902 -2.97066302924355 2.72439362870592
C 1.16136524770707 -3.75455204591524 1.38107543727115	C 1.222992000 -3.721287000 1.623933000	C 1.67296036421859 -3.90357502813991 1.83596402067125
C 0.58485838411118 -3.80862955123552 0.10789153431166	C 0.649644000 -3.824809000 0.347183000	C 1.11756888506236 -3.98006053125507 0.54846183391268
C -0.59105119683476 -3.10115329376529 -0.17789973863014	C -0.509870000 -3.117037000 0.012442000	C 0.06930489100290 -3.15154813911337 0.15228574441696
C -1.12667353487059 -2.30430649002058 0.82600708198867	C -1.035243000 -2.255314000 0.965709000	C -0.40030750170569 -2.22522906827122 1.07137011061594
O 1.10383608121212 -2.92096605808012 3.61921320155483	O 1.128436000 -2.719861000 3.824476000	O 1.62417812246752 -2.83336837006564 4.01231292080445
O 1.21870663595781 -4.59105091594961 -0.81584875968567	O 1.274234000 -4.664125000 -0.525677000	O 1.61179334320694 -4.88460456230695 -0.34610950605982
H -2.01956200016515 -1.73933389117569 0.60145242673176	H -1.921118000 -1.694182000 0.708593000	H -1.20047469089296 -1.56408815560614 0.77077941672877
C -1.26180667586996 -3.20195704985628 -1.53294308981683	C -1.186810000 -3.304023000 -1.330814000	C -0.51804231215124 -3.27188551377394 -1.23702042634305
C -3.05125528226621 1.42121012747665 -3.29662721496417	C -2.977570000 1.185140000 -3.367614000	C -2.36304454872694 1.12380234950989 -3.35345600549866
C -2.99236054512378 3.25134719776902 1.38209057584727	C -2.909415000 3.302172000 1.154368000	C -2.34351457794165 3.31774271128599 1.27759153652467
H -1.22610468206278 4.32820596327601 1.77388709806059	H -1.163139000 4.422443000 1.470554000	H -0.60445835000110 4.45559382141198 1.54744531488248
C -2.73209335256628 -1.40939823248618 3.20387847870689	C -2.640280000 -1.207497000 3.265068000	C -2.00112644025486 -1.15096557291988 3.38709174944147
H -0.88495500869475 -1.79936203889574 4.13577737188607	H -0.809909000 -1.534579000 4.236171000	H -0.15777702608716 -1.43545140863894 4.33803914966206
C -2.79061104274661 -3.24499808661072 -1.45656459402320	C -2.716329000 -3.328601000 -1.244277000	C -2.04886482254486 -3.28581090186605 -1.22931338344881
H -0.96215811086918 -4.15218144818928 -1.96764107118596	H -0.906841000 -4.288296000 -1.695449000	H -0.21773610759393 -4.24243880253923 -1.62370293286314
C -3.70365696494695 0.59839469488998 -4.40474401074427	C -3.639842000 0.258781000 -4.384867000	C -2.95574233384293 0.13271159391418 -4.35248521688896
H -3.36432941139942 1.00985091843518 -2.33719347599428	H -3.279166000 0.866831000 -2.370220000	H -2.68936206745789 0.84418008772410 -2.35150754855745
H -3.44760391599065 2.43956403674475 -3.32796408249845	H -3.376403000 2.195150000 -3.491454000	H -2.79362763626388 2.11161655747021 -3.53848920025955
H -3.38446971805702 4.02831910593784 0.72065673981828	H -3.304117000 4.030533000 0.441643000	H -2.77843544609532 4.05939086426276 0.60250537920218
H -3.32035553727975 2.30378297562843 0.95545984055184	H -3.229439000 2.326201000 0.790764000	H -2.66554315874411 2.34807002400369 0.89814860382185
C -3.63373695115452 3.39651134283179 2.76025787490140	C -3.556004000 3.534979000 2.517843000	C -2.93242616528862 3.51174805508517 2.67239739757622
C -3.39662925871732 -0.63697008803152 4.34066818248096	C -3.307253000 -0.360837000 4.346179000	C -2.64920337910184 -0.25989767756264 4.44329972835533
H -3.13619047965627 -1.04318800121021 2.26032579227512	H -3.037912000 -0.901463000 2.298041000	H -2.41652369270995 -0.89112722949536 2.41379966850953
H -3.02986575405311 -2.45970851509578 3.26447333556278	H -2.940525000 -2.250953000 3.392169000	H -2.29717010035466 -2.18795129398521 3.56700303449420
C -3.37273074140566 -4.35265649625275 -0.58193323373911	C -3.303400000 -4.365656000 -0.289918000	C -2.68246461584526 -4.37603303261400 -0.36808580745000
H -3.18727074921442 -2.29064985279545 -1.11051989672080	H -3.103969000 -2.348527000 -0.967479000	H -2.44077619250310 -2.32522277856798 -0.89635319152486
H -3.16363516342675 -3.36566683177951 -2.47721288086035	H -3.093629000 -3.520899000 -2.252080000	H -2.38581961078408 -3.40389563428162 -2.26242687633716
H -1.30079780627719 1.97388633231272 -4.32823804727841	H -1.249115000 1.662119000 -4.461497000	H -0.61644163828310 1.63789811615250 -4.40485403821301
C 2.09560186407131 2.17269603822806 4.72806277760863	C 2.035534000 2.480513000 4.767638000	C 2.94809627745798 2.47787342633358 4.40412592321569
C 2.44774308473555 -4.46642378056984 1.68770416775589	C 2.447122000 -4.526023000 1.940012000	C 2.84980306925575 -4.77648261940987 2.17605894540070
H 1.22941655262244 4.08131098226479 -3.98444177495926	H 1.435261000 3.499948000 -4.291730000	H 1.45309230382100 4.14164064738547 -4.36226113292452
H 1.29025523537343 4.08693680908569 3.91627482638079	H 1.473474000 4.378883000 3.370515000	H 1.32038469252448 4.54694127817205 1.83230819858960
H 1.04210262828273 -1.34604590667524 4.40506356838175	H 1.073251000 -1.040498000 4.516309000	H 1.73931797951418 -1.04836884225573 4.46807906958693
H 1.74658608232132 -3.72931391478049 3.66537908962260	H 1.956122000 -3.211780000 3.912737000	H 2.43568595933256 -3.34183900212785 4.12749688114239
H 1.01930991803347 -4.29302835006309 -1.73016533060475	H 1.043270000 -4.437413000 -1.448045000	H 2.15310360960199 -5.55780180064757 0.21305679087814
H 1.39998567619685 -3.70210987383001 -4.22292974651562	H 1.572992000 -4.031390000 -3.753663000	H 1.72713146540089 -4.11407444687778 -1.96471216704547
O 0.85392212323578 4.92121351345376 0.51122781924503	O 0.878618000 4.979691000 0.160415000	O 1.00979948258197 5.29118157556105 0.17563659661824
C 1.98748598150566 -1.72979062068337 -5.07657053360412	C 1.894012000 -2.093957000 -5.170507000	C 2.98150665087409 -1.79646974980348 -4.60104210602292
H 0.65233434748526 1.68162829063778 -4.69880078707361	H 0.654024000 1.333060000 -4.851023000	H 1.34301883858709 1.49882553613432 -4.62124760307463
C 1.95142661981154 4.89009855735175 -2.04067712534152	C 1.890044000 4.932489000 -2.403844000	C 2.13564686271957 5.22282350068884 -2.54236854492967
H 0.72749017999258 4.61739494343611 1.43616732177431	H 0.746577000 4.730633000 1.095935000	H 1.73100655799699 5.81322465001631 -0.19581027918029
N 1.95531527711351 -3.11496694883506 -5.58680049292461	N 1.406193000 -2.406595000 -6.512149000	N 2.71381934416855 -2.14690226174387 -6.00318898098183
N 1.88025943431973 5.40671572616474 -3.42213321592158	N 1.423129000 6.227959000 -2.936965000	N 1.87771687306652 5.63572107978447 -3.93069492970178
N 1.96150467057459 3.54740208902182 5.24915657491168	N 1.592171000 2.771110000 6.129364000	N 2.63586375684235 3.30886453648393 5.57629011279470
N 2.44814818707073 -5.02206245760681 3.05598728381951	N 2.165363000 -5.924020000 2.258639000	N 2.71737897263139 -6.12375884644285 1.59929343512227
H 2.28815613559559 1.46638971492275 5.53705086467218	H 2.776952000 1.687446000 4.829521000	H 3.56122082543759 1.61508489388700 4.69437040198724
H 2.97662068906654 2.16393787437689 4.08275852195963	H 2.574759000 3.349435000 4.340985000	H 3.54880556773703 3.09257078936225 3.73617438655544
H 2.65183891558709 -5.24446956008883 0.95065287512410	H 3.099279000 -4.516359000 1.069678000	H 3.75719419348590 -4.33754653543920 1.75765817187148
H 3.27484550687034 -3.75501808843034 1.63805088861238	H 3.037675000 -4.054424000 2.750110000	H 3.01446126644563 -4.85902070866119 3.25794139660936
H 2.07658609256651 -1.01658419363751 -5.89759939697324	H 2.568758000 -1.245773000 -5.259940000	H 3.58401222816322 -0.88974754305482 -4.61504810990339
H 2.89467091089655 -1.63528633881838 -4.47581874359314	H 2.519114000 -2.923746000 -4.784856000	H 3.57638008329359 -2.57882823371674 -4.11294595768946
H 2.10900895755400 5.70324825365867 -1.33063966447440	H 2.535850000 5.100345000 -1.544474000	H 2.25343664051432 6.11381835725381 -1.91207784569951
H 2.83340031137993 4.24830351488368 -1.98782250772700	H 2.535421000 4.442495000 -3.139932000	H 3.09991685515906 4.71173367377469 -2.53549422509144
C -5.13962138391578 3.19784400950665 2.65389862235378	C -5.061533000 3.329979000 2.416336000	C -4.43931815715952 3.29813449069971 2.63004231810276
C -3.30671674829347 4.72661199605484 3.42428493395739	C -3.231856000 4.905180000 3.096224000	C -2.59151238782287 4.86970011095122 3.26967831296153
C -4.91142130464694 -0.71038399988128 4.20316807627608	C -4.821422000 -0.448613000 4.211898000	C -4.16458192693929 -0.38529869584337 4.36454615186325
C -2.95176328399228 -1.11959204903017 5.71429087504375	C -2.863944000 -0.747195000 5.750574000	C -2.15011642701431 -0.55622592653862 5.85011206965024
C -4.89325163138296 -4.26571358866024 -0.58124397613629	C -4.823964000 -4.280281000 -0.311002000	C -4.20013667678592 -4.26034649876727 -0.42389740283113
C -2.90703693362533 -5.73794587743405 -1.00827833986902	C -2.833385000 -5.780024000 -0.599771000	C -2.23110701177733 -5.77528805986099 -0.76408687621498
C -5.20749886053194 0.51973518664496 -4.17733087427213	C -5.141098000 0.205303000 -4.133137000	C -4.46640743135332 0.05854488663700 -4.17356680957692
C -3.39250101544145 1.13816532532401 -5.79354967111553	C -3.342386000 0.660346000 -5.822484000	C -2.59335295743052 0.46914582697378 -5.79240936542349
H -3.30461150615968 -0.41730579257166 -4.34337801500419	H -3.242597000 -0.747060000 -4.227469000	H -2.54547042293463 -0.85473947225482 -4.12890426706460
H -3.23683217851606 2.60021465518397 3.39440236263612	H -3.164354000 2.781289000 3.205066000	H -2.50388203837583 2.74651068808905 3.32262799879121
H -3.10329068625408 0.41130987149820 4.24592738244316	H -3.016693000 0.679067000 4.180388000	H -2.38922748118412 0.77452631395547 4.21101967851137
H -3.02837424517372 -4.18499436871868 0.44145498072162	H -2.970494000 -4.118366000 0.720960000	H -2.37189427459078 -4.20479346273867 0.66618367003391
H -5.59225096707646 3.97478952163565 2.03477645529559	H -5.512582000 4.072864000 1.755872000	H -4.92118393516870 4.05142529396691 2.00385686959877
H -5.38022485044901 2.23386639794309 2.20517769860713	H -5.299823000 2.343502000 2.018153000	H -4.68844729703295 2.31866312849259 2.22152121830174
H -5.61476244675965 3.23704475993150 3.63423030019718	H -5.539424000 3.419890000 3.391797000	H -4.87720007064915 3.36541227788612 3.62586263080735
H -2.24200771699081 4.83470881688319 3.62302921429294	H -2.169249000 5.024128000 3.300277000	H -1.52194858980038 4.98861839692868 3.43449419328781
H -3.61518871103087 5.56235751803233 2.79300893327541	H -3.529986000 5.697836000 2.407260000	H -2.92118918612809 5.67772883412948 2.61341307986568
H -3.82772778233942 4.82127043673845 4.37706464847952	H -3.762575000 5.064357000 4.034806000	H -3.08448918780097 5.00300553424320 4.23255165352013
H -5.23958754048320 -0.33963374269716 3.23191410444705	H -5.150265000 -0.157416000 3.214097000	H -4.53027402071083 -0.14887514189691 3.36511071862416
H -5.26136316221728 -1.73964223183680 4.30231096281715	H -5.168739000 -1.467770000 4.391766000	H -4.48487900255468 -1.40130607861702 4.60323347818363
H -5.40695274449027 -0.11643887724546 4.97120733343912	H -5.319276000 0.202334000 4.930428000	H -4.65339863304172 0.28996090988245 5.06661493950743
H -3.15856045347951 -2.18461594970537 5.83752563756335	H -3.058684000 -1.804205000 5.942551000	H -2.30526736350464 -1.60618491668616 6.10708202342905
H -1.88618847493560 -0.96603521553226 5.87526621664252	H -1.801807000 -0.568904000 5.908276000	H -1.08946500884235 -0.33757650479685 5.96260031048080
H -3.48169736661050 -0.58553564348324 6.50301925691753	H -3.405151000 -0.169189000 6.499449000	H -2.68493362433993 0.04642000588856 6.58404136391523
H -5.23393411266993 -3.28340301877932 -0.25235291439773	H -5.168717000 -3.274373000 -0.068966000	H -4.53466689848101 -3.26769290489834 -0.12105712455952
H -5.29301657709763 -4.43795049661578 -1.58237229360983	H -5.214429000 -4.535891000 -1.297796000	H -4.56542952335305 -4.43889355941773 -1.43690832493117
H -5.33153811567369 -5.01123020123425 0.08237440285085	H -5.267530000 -4.968044000 0.408813000	H -4.67638710832717 -4.98886828793033 0.23231779681524
H -3.15627546758251 -5.92637114128147 -2.05447631206222	H -3.062546000 -6.050063000 -1.632649000	H -2.44464908536149 -5.96832383482801 -1.81719540713787
H -1.83121055813396 -5.85946346768580 -0.89458462127981	H -1.760678000 -5.896425000 -0.454372000	H -1.16361459708812 -5.91947433994757 -0.60878853613457
H -3.38838288036364 -6.51043079436015 -0.40850125155523	H -3.328899000 -6.502289000 0.048822000	H -2.75244117385208 -6.53110197810082 -0.17654985888183
H -5.43857763806897 0.09979057466968 -3.19782120800044	H -5.359601000 -0.121680000 -3.115987000	H -4.73149968020021 -0.21179326160190 -3.15088225108301
H -5.66363940141877 1.51034986171545 -4.22880025378097	H -5.596799000 1.187601000 -4.271074000	H -4.93300111587253 1.02033229800853 -4.39483757817088
H -5.68867571390210 -0.10446712775999 -4.93044204821093	H -5.631275000 -0.485935000 -4.818659000	H -4.90626295797559 -0.68356449295973 -4.83985013958717
H -3.70902532292670 2.17925286075602 -5.88569036503092	H -3.655324000 1.689404000 -6.009884000	H -2.91198374050179 1.48188402165811 -6.04758947065275
H -2.32914515314586 1.09244114635734 -6.02263093079476	H -2.282497000 0.585143000 -6.059850000	H -1.52159716727088 0.40085425526707 -5.97035321162423
H -3.91570204149331 0.56444641211444 -6.55867454556735	H -3.877875000 0.018689000 -6.522101000	H -3.08220473213510 -0.21644580179077 -6.48450344619376
C 3.26679930014926 -3.54461089084789 -6.05856013934608	C 0.539175000 -3.570817000 -6.525929000	C 2.00840743629115 -3.41055880899355 -6.12523878805436
C 0.93249287189653 -3.27891561841496 -6.61810513693694	C 2.522808000 -2.577397000 -7.425158000	C 3.95434932212977 -2.17710594407134 -6.75576945510682
C 3.79641358301278 -5.36116303556455 3.49728007363266	C 1.384877000 -6.063725000 3.473952000	C 1.67096864364865 -6.89696396375505 2.26586074897008
C 1.54473165261425 -6.16551741507291 3.16737740761948	C 3.400735000 -6.683453000 2.341709000	C 3.99230120733918 -6.83415781889976 1.59156215218654
C 3.19317823724235 5.81018474526700 -3.91330651195169	C 1.811309000 6.445605000 -4.318763000	C 3.06220503330213 6.22812976212674 -4.54406407776036
C 0.90774581774281 6.49205257517693 -3.53581616773708	C 1.825673000 7.345112000 -2.097880000	C 0.72148243293172 6.52554027909476 -4.02317028967173
C 0.97778541271717 3.61527838714196 6.32830112424251	C 2.735370000 3.010505000 6.992458000	C 1.92259046955079 2.56444163287681 6.60023620774422
C 3.24933188301468 4.09362602763439 5.66228328584914	C 0.658204000 3.880895000 6.180806000	C 3.85631319860449 3.88073209344831 6.11607870375355
H 3.98634496711278 -3.48272081182603 -5.24580015439633	H -0.319589000 -3.406051000 -5.880298000	H 1.06873736650223 -3.36750881256197 -5.58058509224262
H 3.62435263821685 -2.92746451359773 -6.89122856195797	H 1.052315000 -4.488229000 -6.189473000	H 2.58787846356947 -4.26305356527400 -5.73526184817935
H -0.03220080069968 -2.96750702225241 -6.22673464609926	H 3.131299000 -1.674903000 -7.440042000	H 4.44524408242311 -1.20656158214663 -6.70190472682213
H 1.15679336618968 -2.68029768915580 -7.50817172621331	H 3.173744000 -3.423571000 -7.148186000	H 4.66369495762814 -2.93751704670682 -6.38577397581424
H 4.42335776112307 -4.47302063379793 3.47741434850965	H 0.443086000 -5.529646000 3.378993000	H 0.73458397715833 -6.34698192189316 2.23657905440308
H 4.25386265225790 -6.12723349410053 2.86019385105361	H 1.911823000 -5.678325000 4.364313000	H 1.92497242862030 -7.10043430572272 3.31225162221584
H 0.54921296693881 -5.87681432044265 2.84156274821611	H 3.944366000 -6.609953000 1.401703000	H 4.72523016674917 -6.26377749056369 1.02667273071846

H	1.88222721209314	-7.00751222846988	2.55239884261834	**H**	**4.064926000**	**-6.332816000**	**3.149380000**	H	4.37679378074775	-6.99534132838851	2.60534967521594
H	3.62335679520168	6.61866087537935	-3.31081385700373	**H**	**2.903628000**	**6.472945000**	**-4.465026000**	H	3.37316510190844	7.14418149401436	-4.02851675847310
H	3.87038834893677	4.95993652755186	-3.88890591036226	**H**	**1.402088000**	**5.667154000**	**-4.960630000**	H	3.88336246034161	5.51642518152333	-4.51772127312360
H	-0.05852972978064	6.15525494963910	-3.17022200179904	**H**	**1.478161000**	**7.187710000**	**-1.081509000**	H	-0.14419246154989	6.05007931444243	-3.57061885037366
H	1.21328991439908	7.36976737568747	-2.95528202978507	**H**	**2.917976000**	**7.491452000**	**-2.069796000**	H	0.90485353280155	7.47770175443238	-3.51227691504950
H	0.03015426679951	3.21037459545557	5.98345002148299	**H**	**3.394679000**	**2.144385000**	**6.983385000**	H	0.99618538750022	2.16573116452614	6.19510554926191
H	0.82830251936882	4.65202368061874	6.61896312736667	**H**	**2.397441000**	**3.172158000**	**8.014176000**	H	1.67520888036677	3.22980103259851	7.42591415799093
H	3.93325828782583	4.09702686688972	4.81707986852949	**H**	**-0.214377000**	**3.666495000**	**5.569115000**	H	4.35166206154827	4.48437827734606	5.35700324728319
H	3.11586005328402	5.11665854081212	6.00505481529218	**H**	**0.325648000**	**4.030897000**	**7.205785000**	H	3.62064615337401	4.52324440685146	6.96289042668140
H	3.21028795571187	-4.57704212879931	-6.39441866133733	**H**	**0.175881000**	**-3.742632000**	**-7.536925000**	H	1.78632992667332	-3.60268056944846	-7.17391141892825
H	0.87018706226516	-4.32547757219558	-6.90522412763310	**H**	**2.151068000**	**-2.756121000**	**-8.432140000**	H	3.74671862057048	-2.39681298086153	-7.80191212829671
H	3.75977450420509	-5.73744770712360	4.51658641833155	**H**	**1.162732000**	**-7.114589000**	**3.647329000**	H	1.53487939987545	-7.84226538766818	1.74766108560872
H	1.49203531212751	-6.48776916644184	4.20424524968866	**H**	**3.175474000**	**-7.732069000**	**2.525709000**	H	3.86175715667978	-7.80151650108955	1.11398873302142
H	3.10532989538935	6.15473648426272	-4.94066513785491	**H**	**1.405485000**	**7.394471000**	**-4.665160000**	H	2.84648596225098	6.47169234688903	-5.58091498751053
H	0.80439834812437	6.77983204799801	-4.57897030341219	**H**	**1.375842000**	**8.262871000**	**-2.473488000**	H	0.50228994805239	6.72540164596396	-5.06845956287865
H	1.30184307534970	3.04494837703033	7.20612301122209	**H**	**3.324588000**	**3.891398000**	**6.686941000**	H	2.50922059100813	1.72241287938739	7.00116779045742
H	3.69886873422776	3.51049105338941	6.47451283039415	**H**	**1.102511000**	**4.827519000**	**5.827258000**	H	4.57171313417350	3.11477618413391	6.46107971502493
					**2**						
C	-0.81281993146946	-2.08830159953460	-2.51550041112095	C	-0.59475192161429	-2.23120342143357	-2.47052150751769	C	-0.36038119761067	-2.23180370565481	-2.08553215254893
C	0.22021021727855	-2.35539015356081	-3.41558660452693	C	0.37338679984560	-2.56898085125018	-3.41360443657457	C	0.75077905339393	-2.54417455912506	-2.88402534274267
C	0.71663735093934	-1.36995461529840	-4.27674196358186	C	0.86070018434546	-1.65739946155744	-4.34824839597928	C	1.25241909577641	-1.62805027177580	-3.81465216256258
C	0.14771118561030	-0.09287877922375	-4.24328295114720	C	0.32345995501093	-0.36361944922419	-4.35172355889821	C	0.66216840081893	-0.36131327905287	-3.90583293966167
C	-0.90892786923343	0.20069494853632	-3.37066927102759	C	-0.68459995429841	0.00430109188515	-3.45183430097347	C	-0.44711598959909	-0.02031796061786	-3.12374320221742
C	-1.33459106365704	-0.80375305721394	-2.50983857345904	C	-1.08753292067129	-0.93591700120341	-2.51348182610824	C	-0.90930286184466	-0.96664056346058	-2.22123558068194
O	0.76764450969175	-3.60815823214263	-3.44321385874532	O	0.85798316689696	-3.85934537521642	-3.37109342830637	O	1.36088480021399	-3.76706010379577	-2.82814717290332
O	0.66699906714843	0.83596729210929	-5.10316606013482	O	0.80456761354308	0.50283040037312	-5.29145600220946	O	1.21483420405450	0.50367068528929	-4.80756129387549
H	-2.13009374806244	-0.57457927706550	-1.81538499927678	H	-1.84808042548563	-0.65299143135611	-1.80053740821856	H	-1.76772473123783	-0.71162309429045	-1.61798974128880
C	-1.57645042613693	1.56328667470607	-3.35630099608481	C	-1.35573365466915	1.36453827190481	-3.51875691592452	C	-1.14486340099025	1.31493674891226	-3.27457032020658
C	-0.97794677007845	2.45178811237145	-2.28693748438528	C	-0.77216915910450	2.32442368101095	-2.50279274646331	C	-0.58928098599913	2.34093927050437	-2.31090158638388
C	0.12319084933568	3.25398681336022	-2.58945873552942	C	0.31991699829136	3.12795008978687	-2.83342970432173	C	0.39833315447144	3.22911386832356	-2.73782278060163
C	0.74046628051644	4.05066630432906	-1.61860609733143	C	0.90673906851586	4.00321468936781	-1.91996961985081	C	0.93480695912570	4.20357814517101	-1.88193862155793
C	0.22023737334975	4.06618334102597	-0.32128158531655	C	0.36936726433996	4.06810126780197	-0.62568598342428	C	0.44666694180547	4.27029621750987	-0.57775565780451
C	-0.90946696032807	3.30150142553722	0.00839469622495	C	-0.75079331687216	3.30861154717013	-0.26397437552760	C	-0.52675689072570	3.38776515250727	-0.10418521474044
C	-1.45369540957133	2.49398308785042	-0.98392560885230	C	-1.27436339300506	2.44313464658936	-1.21600641525172	C	-1.00789286596644	2.43361013525763	-0.99065280917104
O	0.61808777583812	3.25798039040571	-3.86418301214612	O	0.79143528958127	3.02254803744496	-4.12714921531147	O	0.84853013911106	3.14811098750786	-4.02283266374988
H	-2.30016775294244	1.87444501695629	-0.72893885389293	H	-2.12532747303701	1.83896358433246	-0.94276703179360	H	-1.74830759579855	1.73180329028940	-0.63570554258793
C	-1.49359581443476	3.33820162631978	1.40742453612053	C	-1.35935809108298	3.43160034698120	1.11953232450026	C	-1.01237509112063	3.46966119610909	1.32993226033714
C	-0.83093849759524	2.30864371772600	2.29677365869738	C	-0.70144527107581	2.48366961069072	2.10145817289298	C	-0.28833786906020	2.48265951745383	2.22761115782064
C	0.21619899241782	2.66966826189539	3.14251812228516	C	0.25807510975378	2.93072481679932	3.00880895862062	C	0.84779218629242	2.85967344158031	2.95728369973067
C	0.85999901007570	1.73008866961335	3.95605056032831	C	0.85814306326175	2.08062154127821	3.93733212017114	C	1.46197322682894	1.97882538776119	3.85260959354427
C	0.41536248236192	0.40550659657430	3.94383386468538	C	0.47954710574318	0.73026660709104	3.93777974005435	C	0.95705003877195	0.67935197172502	3.97249677767961
C	-0.66417138825277	0.01919972752157	3.13640781163338	C	-0.51303048779869	0.25239754024076	3.07431736105199	C	-0.17678243833439	0.26826829153430	3.26185527728833
C	-1.22799751114132	0.98021880409729	2.30872876399796	C	-1.05193647072593	1.14485574154385	2.15873621242422	C	-0.75320028197682	1.18641417529271	2.39471972943897
O	0.63290436446919	3.97285893564804	3.16747317810437	O	0.58413516503946	4.27155128977770	2.94095441297605	O	1.37997328201544	4.11792049640198	2.86269835708187
O	1.06992146886019	-0.47418187508361	4.76205376434639	O	1.11060904749182	-0.07987947326522	4.83669821796658	O	1.61442563196127	-0.15034036662093	4.83679477797048
H	-2.04424339103239	0.68593787628446	1.66410375000342	H	-1.81182980622258	0.78904438071353	1.47756131522768	H	-1.64001531021404	0.88986977623419	1.85377557806386
C	-1.21237026929811	-1.39350275070346	3.16717281181404	C	-1.03062771759674	-1.17074358697841	3.16591871779360	C	-0.77904097149547	-1.10834793358383	3.46900412859918
C	-0.61094306040349	-2.23202260361144	2.06175418353870	C	-0.40021670648899	-2.06497755914550	2.11974014636680	C	-0.25419464362979	-2.11264327612016	2.46252352969842
C	0.55762212100045	-2.95761651222838	2.29838851893804	C	0.77516980576081	-2.76554488726238	2.39883026523542	C	0.79724986344901	-2.97193728325039	2.79096886592064
C	1.16702717199335	-3.71764941127380	1.29413104532442	C	1.39431902394641	-3.58748011582207	1.45802276297458	C	1.30587550341629	-3.90191576428798	1.88451955730905
C	0.57271500430197	-3.77275501183013	0.02982637941762	C	0.80232534579986	-3.70812982142215	0.19112762427199	C	0.72195061584417	-3.96389141030818	0.60845196476626
C	-0.61918547891443	-3.08202342191729	-0.23167040876592	C	-0.39538503968428	-3.05360274544815	-0.11551553356311	C	-0.33642069248932	-3.13172228908672	0.24664151240526
C	-1.15536025350188	-2.30652614976544	0.78857342915297	C	-0.94638255443931	-2.23064263764129	0.85774566217302	C	-0.78295321536046	-2.21215123062204	1.18361736274005
O	1.12455606505739	-2.92400791920749	3.54272512553334	O	1.29401180170422	-2.61058231063298	3.66907791834221	O	1.31496798283595	-2.84727580986925	4.06770451518601
O	1.20358445864901	-4.53958326702844	-0.90935129798226	O	1.45431266225276	-4.50880961996770	-0.69901734781299	O	1.19326857070291	-4.86293813722316	-0.30115665028404
H	-2.06085807443219	-1.75489762837280	0.58235186800907	H	-1.86374011112327	-1.71285208767619	0.62286542771986	H	-1.59140694220428	-1.55074711451993	0.90725721202946
C	-1.30237321748807	-3.17415378851441	-1.58066777930870	C	-1.07755660298103	-3.24612312021879	-1.45448552001045	C	-0.95339277015412	-3.24702342075414	-1.12915659036717
C	-3.10272810506181	1.47498546019453	-3.27736365761345	C	-2.88216230108063	1.26223424899222	-3.43706388977479	C	-2.67240671240416	1.21901255092826	-3.20623471789974
C	-3.02128007235660	3.24050181964039	1.42698423643554	C	-2.88564748636712	3.30123268028398	1.12126017994677	C	-2.53828447116063	3.37799524783439	1.43212328190873
H	-1.25345230344900	4.31551558632610	1.81937265575795	H	-1.15731771789279	4.44413225164622	1.45820556357900	H	-0.76714826271485	4.46650677501989	1.68416086028999
C	-2.74221377601516	-1.44098615791242	3.18933777180560	C	-2.56114826393536	-1.23551716143189	3.15946160887627	C	-2.30977386961020	-1.09823661875565	3.52951928685107
H	-0.88678752964505	-1.84474701399479	4.10104548256508	H	-0.72973786478658	-1.56223462800207	4.13348848439997	H	-0.46311709824678	-1.45427454886968	4.44914306143295
C	-2.83019327809693	-3.22256974244711	-1.49295702487380	C	-2.60638628687636	-3.28904717080716	-1.36799287570174	C	-2.48396164977439	-3.22081532669189	-1.10268955680789
H	-1.00244411817467	-4.11914292328963	-2.02678016368877	H	-0.78456956269840	-4.22411560706350	-1.82657011483553	H	-0.68556495657842	-4.22915556801691	-1.50978455763069
C	-3.76187672044121	0.67297006293729	-4.39673960632003	C	-3.52682142297876	0.39877167555240	-4.51887000927267	C	-3.31030050014564	0.24217195087416	-4.19152906589033
H	-3.41169322874132	1.04608947157895	-2.32430992535124	H	-3.18543451423217	0.87355700150174	-2.46540581190912	H	-2.99956153465364	0.95585029105497	-2.20010761510645
H	-3.49768397762816	2.49434936615405	-3.28847452254766	H	-3.29013837225524	2.27451049977101	-3.49560147712471	H	-3.06889377741023	2.22079024062752	-3.39321541423929
H	-3.41858547038038	4.02071309584477	0.77245611436861	H	-3.28574209118005	4.02472608906155	0.40647523172496	H	-2.95848672041689	4.13149263840620	0.76071642669798
H	-3.34976869495658	2.29442739079220	0.99729107885267	H	-3.18673369279250	2.32014335881672	0.75522182760445	H	-2.89283159595266	2.41758110871336	1.05838058345431
C	-3.65454154285675	3.37523279192363	2.81001976476198	C	-3.54631078281840	3.51973130577402	2.48055753046442	C	-3.10638586616769	3.58928602501266	2.83316375081690
C	-3.39867826309870	-0.68122192927902	4.33940774263660	C	-3.24767862218407	-0.43153989731452	4.26086296420520	C	-2.90969903255792	-0.19774850240542	4.60632749609374
H	-3.15295765116742	-1.06318981454393	2.25323912842043	H	-2.95533203620061	-0.90120986324367	2.20039598127173	H	-2.73151415670209	-0.81179243591608	2.56648454087217
H	-3.03970779903186	-2.49194464806328	3.23980460038961	H	-2.84927059385524	-2.28624474716090	3.24966028671532	H	-2.63830900126317	-2.12664810489585	3.70248250735770
C	-3.40267106735033	-4.34070387184580	-0.62517419726003	C	-3.18316776859017	-4.33889737452196	-0.42129980007624	C	-3.13485186593494	-4.27884802201699	-0.21422742001981
H	-3.22666695610394	-2.27248292802889	-1.13488017234234	H	-3.00448608474845	-2.31408424736910	-1.08744348965904	H	-2.84987578831244	-2.24392738573870	-0.78769570845287
H	-3.21051135869525	-3.33417067554658	-2.51194213053288	H	-2.98022264004584	-3.47966903733652	-2.37742262774787	H	-2.83339245406939	-3.35262660605762	-2.13007439515140
H	-1.35506678739283	2.04354752726018	-4.30658180344737	H	-1.14482660257807	1.78215912953403	-4.49931710918241	H	-0.92105488178038	1.68240474647428	-4.27263805828402
C	2.04354833858793	2.16057677586934	4.77147872472016	C	1.86698978563469	2.55930568345042	4.93810381741640	C	2.62677432389524	2.40677312011894	4.69278341199583
C	2.47012897673652	-4.40298147664588	1.58533629845751	C	2.66546795406585	-4.33265219242310	1.74030629549748	C	2.47816672044010	-4.79235230283810	2.20360693341539
H	1.22461546342285	4.10432713423906	-3.91546242156548	H	1.59540409686865	3.54962611379054	-4.23037263753501	H	1.31954095676984	4.04915613658427	-4.20495916220000
H	1.18741063482604	4.05929135347885	4.04096211231901	H	1.32707050360618	4.45902546755396	3.53095243169110	H	1.14297498875714	4.51785990662327	2.00564479944021
H	1.03742288698078	-1.37980698035153	4.38286691987152	H	1.11858157022840	-0.99487952716939	4.49485881540155	H	1.43683587207310	-1.08114000429282	4.60854594440429
H	1.76813795177555	-3.74094794172687	3.56636678738901	H	2.14235201866216	-3.06888008513659	3.73832209913736	H	2.12286492205672	-3.36586929807471	4.15920410527550
H	0.98197251958739	-4.24339001139928	-1.81944700419715	H	1.17678919496392	-4.31819986715246	-1.61418681857899	H	1.74300536758527	-5.54532856147076	0.26016974828461
H	1.31360529366874	-3.64193332532012	-4.33112921152326	H	1.45237183159578	-4.01955704100206	-4.11579278594974	H	1.23380858730192	-4.18147178446887	-1.95004081089871
O	0.86374207531549	4.86862677928812	0.57914666017501	O	0.99175231732442	4.92006034239170	0.23672394943350	O	0.91051879800631	5.21318271716293	0.32207464399441
C	1.89807358157090	-1.67707890888654	-5.15157142558757	C	1.94388758616344	-2.05429954830275	-5.30448564842737	C	2.40337079937036	-1.97270316195146	-4.71149334051915
H	0.59111547387914	1.73520916987027	-4.71334097343951	H	0.73755133428195	1.41646920173841	-4.95182193741893	H	1.01665112630586	1.43056330228953	-4.56681446240531
C	1.98867192031603	4.80564076125072	-1.97377377497372	C	2.09163192108886	4.85690754108295	-2.26600692817691	C	2.04107587513285	5.08521297726481	-2.39149928762290
H	0.70659095614223	4.58131688386072	1.50381689922186	H	0.76769834268678	4.71298499640960	1.16401295665331	H	1.66914325010995	5.68477223152168	-0.04231804269864
N	1.83628450110872	-3.05425681511595	-5.68451475344693	N	1.52354274273364	-2.93306022123382	-6.40050463944612	N	2.00464480459256	-2.26980883489586	-6.09329041482055
N	1.89910091293248	5.39372168572302	-3.32681264276803	N	1.74460685032448	6.24613679375452	-2.55545007767734	N	1.80351719543288	5.51085747759077	-3.78102271579382
N	1.81933868803359	3.48739908609320	5.38269869551502	N	1.29452689924656	2.75607437695773	6.26773927405226	N	2.26882502640165	2.66942944660982	6.09176381564725
N	2.46942546215519	-5.00752051496146	2.93451252799139	N	2.45582489225266	-5.75211648678960	2.01224754262015	N	2.33951462099274	-6.11996027904608	1.57987455150060
H	2.30613547429053	1.40596373378254	5.51020245531955	H	2.64872080489653	1.80817476138961	5.02767558305234	H	3.36380574701366	1.60452567720229	4.70440009342277
H	2.90962939968061	2.26862467525781	4.11450560650315	H	2.37856389170903	3.46776870063224	4.57227950768599	H	3.09928159848073	3.27478932927683	4.22395545972208
H	2.71513341749669	-5.12709718737756	0.81112418643760	H	3.30462091808883	-4.26071176847829	0.86308154119871	H	3.39235273845271	-4.34566278534625	1.80876390843204
H	3.27136486218862	-3.66093025705056	1.58376563203909	H	3.23398465935594	-3.84069889759301	2.54980418201228	H	2.62693794419585	-4.90053222100343	3.28342479134857
H	2.00154679478870	-0.93735029908512	-5.94311592246121	H	2.40102263710022	-1.14877952433196	-5.70335446921807	H	3.07119703418224	-1.11373703183862	-4.75768448073589
H	2.80932009402563	-1.61704902861394	-4.55238069206205	H	2.73823888188847	-2.54558269228854	-4.72684240213763	H	2.96516256318907	-2.79923118200190	-4.26703125004231
H	2.22597407235362	5.55092807506894	-1.21758054458819	H	2.76847493869345	4.86181770678064	-1.41462497562387	H	2.21533313581316	5.95416222903524	-1.74885132902336
H	2.82873101255505	4.10810580529209	-1.99731328927466	H	2.66891956547285	4.39781454275812	-3.08872206724096	H	2.97511459856840	4.52069759535741	-2.39158528726465
C	-5.16050193395016	3.17344316258075	2.71186690430172	C	-5.04716937482529	3.28853208198740	2.36782594542100	C	-4.62007709081061	3.42717089344344	2.80671395629386
C	-3.32677687148944	4.70149658450431	3.48126183134859	C	-3.25151109137990	4.89390822108334	3.06561833300886	C	-2.71289769585753	4.93315561313634	3.42978221532198
C	-4.91441116197054	-0.75406954479062	4.21269533791205	C	-4.75957794485918	-0.52796488361775	4.10632759894385	C	-4.42963956322260	-0.25895643348200	4.54257783840994
C	-2.94279331223138	-1.17703629436272	5.70463592726787	C	-2.81776348305508	-0.86069622019782	5.65700596033658	C	-2.40997826150959	-0.53683850341359	6.00344912009280
C	-4.92347998699151	-4.25970721771693	-0.61435294747831	C	-4.70460124931694	-4.27200643242668	-0.44596251542220	C	-4.65075770156885	-4.14763088801171	-0.28208948696478
C	-2.93372147868831	-5.71996787428187	-1.06709876061002	C	-2.69412314206827	-5.74503258298981	-0.73882438605788	C	-2.69695146349295	-5.69410241631269	-0.56654025932287
C	-5.26498989251598	0.59423403734445	-4.16478357588299	C	-5.02407992964919	0.28297063002451	-4.26594600958951	C	-4.82404324650388	0.25500911978912	-4.02561799182806
C	-3.45514458829934	1.23521112119891	-5.77765196005523	C	-3.25290398917313	0.91930852352390	-5.92262887237658	C	-2.91710454765162	0.52465847859950	-5.63482889543541
H	-3.36475173595817	-0.34447846562112	-4.35437515624542	H	-3.09797847395536	-0.60383488248578	-4.44369041838735	H	-2.95544633173376	-0.76059537692439	-3.94312841399113
H	-3.25089029239800	2.57549114950195	3.43557464644543	H	-3.14556905383254	2.77078592958766	3.16795941062710	H	-2.69781347189370	2.80839175733217	3.47763124413527
H	-3.10664914405093	0.36819352993969	4.25319606108353	H	-2.96451760262469	0.61594747728970	4.13243040796404	H	-2.60740572200156	0.82830973001302	4.38820106020834
H	-3.05280578078119	-4.18133350107001	0.39764645021917	H	-2.85636337162272	-4.09446247740632	0.59215822064913	H	-2.82843715307341	-4.08226007680768	0.81663976320486
H	-5.61858296252591	3.95281263286518	2.09982947540292	H	-5.50633193148962	4.02459049322756	1.70526487370376	H	-5.08335620727067	4.19842348773184	2.18837711608332
H	-5.40169356764630	2.21151192899272	2.25912969688520	H	-5.26567597422132	2.29874735356969	1.96659148268379	H	-4.90685328861105	2.45805498578586	2.39824536187218
H	-5.62984098395893	3.20614841156882	3.69524023580762	H	-5.53346962829754	3.36879948122617	3.33997873480384	H	-5.04377822446863	3.50585627467130	3.80779766455769
H	-2.26079773030689	4.81248959147914	3.67085753863983	H	-2.19286242642988	5.03311659532342	3.27639687769624	H	-1.63897602747304	5.01163106366740	3.58852559635588
H	-3.64491747166243	5.54079843607007	2.85951196643141	H	-3.56135128893961	5.68320552375077	2.37793341405853	H	-3.01696983779205	5.75341618790037	2.77632912255783
H	-3.83942304281713	4.78647472979824	4.43950290197717	H	-3.79087205900495	5.03880834360627	4.00161683183925	H	-3.19413632266011	5.08298103035091	4.39616886877586
H	-5.24985205811824	-0.37398034366260	3.24755486785306	H	-5.07980229044465	-0.20687020965419	3.11494493022156	H	-4.79606952844588	0.00187926187914	3.54951528402553
H	-5.26328289526718	-1.78437914562806	4.30440724562373	H	-5.09962868317853	-1.55542037224948	4.24886481114967	H	-4.78881674204647	-1.26327718030011	4.77541917358630
H	-5.40441472022792	-0.16773287521195	4.99009350896463	H	-5.27113005424369	0.09487635704568	4.84001651527728	H	-4.88258023910497	0.42880198883329	5.25652129469131
H	-3.14799928200452	-2.24331985935349	5.81946971384269	H	-3.00741059466353	-1.92477827854712	5.81126414858103	H	-2.60849927039418	-1.58281430532737	6.24647962811404
H	-1.87609205151331	-1.02430819298307	5.85845094098628	H	-1.75902871868405	-0.68057098165811	5.83322774286103	H	-1.34013865009852	-0.36598996716464	6.10896118635226
H	-3.46672803578045	-0.65079269978989	6.50260470599796	H	-3.37197745169276	-0.31147650036710	6.41796328124395	H	-2.91091858253852	0.07768199266580	6.75136093313650
H	-5.26558610282315	-3.28194058000254	-0.27359385639264	H	-5.06198790119591	-3.27233802149894	-0.19649426175793	H	-4.97540111360679	-3.14172686724730	-0.01402282487005
H	-5.32893397392914	-4.42346396863612	-1.61461813207937	H	-5.08945049940418	-4.52405692269371	-1.43589058182505	H	-5.01311359040267	-4.35481447100378	-1.29071363454783
H	-5.35495610430240	-5.01329571077431	0.04461930775210	H	-5.14175815148866	-4.97093388585363	0.26699135754595	H	-5.13898453805655	-4.84846260758279	0.39518031110091
H	-3.18670748605942	-5.89914535929917	-2.11402554586795	H	-2.91490944685709	-6.01075848069391	-1.77465988934057	H	-2.89948795887389	-5.91417403694938	-1.61660385918142
H	-1.85694234396090	-5.83836006308339	-0.95908946445246	H	-1.62062375024279	-5.84879010409918	-0.58896253677269	H	-1.63345312637384	-5.84763157790298	-0.39350468288018
H	-3.40959359278746	-6.50008677697139	-0.47282886652622	H	-3.18351011231542	-6.47793396316244	-0.09755550188124	H	-3.23511046575378	-6.42680708268499	0.03504340618573
H	-5.49362096256991	0.16037921483289	-3.19078316285094	H	-5.22829896812096	-0.12788214498353	-3.27669081568856	H	-5.11306830682418	0.03093087613479	-2.99816828386346
H	-5.71891620788462	1.58660513133218	-4.19993081884953	H	-5.50698704333676	1.26024578170612	-4.32510432813547	H	-5.23529520422995	1.23370326552096	-4.28019009050615
H	-5.75052114195221	-0.01742545194830	-4.92538295900563	H	-5.49936597649998	-0.36534508081533	-5.00201525307405	H	-5.29796937390797	-0.48180691536772	-4.67418996739815
H	-3.76988876124078	2.27830982059199	-5.85131322050322	H	-3.60618618132519	1.94670310400205	-6.03188741408523	H	-3.16661837743998	1.55022402500792	-5.91502054017613
H	-2.39281882171859	1.19102050737122	-6.01161695773532	H	-2.19257549727351	0.90371417728146	-6.16747704249332	H	-1.85109223612592	0.38185969937675	-5.80139646205399
H	-3.98257709111184	0.67520131699562	-6.55002350767928	H	-3.76572262066277	0.31137143945098	-6.66787137743986	H	-3.44450391647518	-0.14271111213297	-6.31641532746398
C	3.15484582172781	-3.52276118819682	-6.12785782453247	C	0.13425982131544	-3.30506761975900	-6.59173728592508	C	1.25193226607659	-3.51903205491574	-6.23963912588071
C	0.75464638344669	-3.22790416225443	-6.67107440028965	C	2.54483177297447	-3.82382476409484	-6.90838803814521	C	3.13215969727209	-2.16434787078707	-7.01592320121675
C	3.83427786537847	-5.28948038372378	3.39686081691366	C	1.75824354893171	-5.96760687633757	3.27381599248886	C	1.30025961444568	-6.91883156097968	2.24919536910971
C	1.53335903752059	-6.14068925666067	3.04429445778435	C	3.68327370728232	-6.53069570419227	1.84460641150965	C	3.62987821075608	-6.80229783795547	1.39423522105610
C	3.22741173639586	5.72608248167447	-3.85696932556524	C	0.94273453274490	6.36796252525135	-3.76718218089772	C	3.03808571902151	6.00115411131017	-4.40916140077603
C	0.91190714397313	6.48581125491241	-3.40245621358305	C	2.91103131075618	7.12765779088145	-2.50033227450504	C	0.64130584999201	6.40941087423565	-3.91268334148952
C	0.75301283757033	3.46600345461615	6.40048272477700	C	2.32344286702947	2.83208710846056	7.30502409178260	C	3.44990871327973	2.75423451288005	6.94676442674744
C	3.08215228612466	4.09002513361634	5.82779402410026	C	0.32070578779893	3.84136838060396	6.28938272848998	C	1.33212587882874	3.78219864244765	6.27305004750264
H	3.83346050005666	-3.37006034036161	-5.28930664450739	H	-0.36348178304426	-3.59000867217836	-5.65799686246652	H	1.58007153984799	-4.26935579163117	-5.51055431191823
H	3.53289092443995	-2.90497424685763	-6.95048197868307	H	0.13079134928723	-4.20428583168936	-7.20761845765017	H	1.48069653163681	-3.93182621254726	-7.22261948001008

H	-0.15455226441318	-2.85727497058952	-6.20040555249129
C	0.95177840467507	-2.54378017494723	-8.01289586397092
H	4.38941621760191	-4.35664874999753	3.30308176852855
H	4.32840742927623	-6.01232748800996	2.73744603590997
H	0.56709517001481	-5.77788313264045	2.69790146905846
C	1.90894362192470	-7.40067330855312	2.28320431060701
H	3.72190027794343	6.47625760295515	-3.22933246796220
H	3.82440992128288	4.81785104707367	-3.78070989094446
H	-0.01975881702962	6.08769048457491	-3.00371773238579
C	1.27244957413526	7.77012773104010	-2.67598876631666
H	-0.12377315464016	3.03324878370914	5.92179701700914
H	0.49851132035803	4.49855746193965	6.62897939528918
H	3.74578402128319	4.08786003207221	4.96341063715837
C	2.92608937707639	5.50715741727347	6.33443211090327
C	3.16927402610362	-4.98414553765615	-6.52066972980221
H	0.61060467739531	-4.29653717129519	-6.81571683983743
C	3.89553308956244	-5.76345052174896	4.83236697399792
H	1.41391307242578	-6.36579259349418	4.10192165113154
C	3.20084230298105	6.18470435366529	-5.29875705671791
H	0.73090679846467	6.69136770206983	-4.45532455214595
C	1.06064370935262	2.71016109263079	7.68193772481483
H	3.56395982725358	3.46678721831249	6.59014902645568
H	1.07835266135158	-1.46805620062322	-7.90827729422884
H	0.07249568964397	-2.71066900248661	-8.63331666516029
H	1.81233945882218	-2.93721302102380	-8.55067137240606
H	2.84075664714921	-7.83441954148405	2.64165872233689
H	2.00750944929887	-7.21864933187972	1.21488673514719
H	1.12699555337480	-8.14697778062022	2.41621042896478
H	2.16729758790921	8.23577251522952	-3.08504999189548
H	1.43029202748793	7.60785019539547	-1.61163672902532
H	0.45552889937093	8.48283687781963	-2.77980680562891
H	2.38742676228256	5.55379462690748	7.27833944185536
H	2.39812662066613	6.12479873088669	5.60883709024545
H	3.90950617911365	5.94465045314265	6.49774278227345
H	2.62102080614382	-5.17463453642978	-7.44082644106873
H	2.73977648365874	-5.60244827813267	-5.73336632459051
H	4.19660613152343	-5.30681422422765	-6.68083362030877
H	3.48112703644772	-6.76115499616023	4.96051563737710
H	3.35720324746958	-5.08249637343220	5.49052404060846
H	4.93353305895488	-5.79758110217708	5.15898925661227
H	2.73912257992447	7.16256604907383	-5.41723866839719
H	2.66141479487102	5.47396125118051	-5.92369666912958
H	4.21999066364503	6.25808079476188	-5.67458212057474
H	1.89885189414799	3.14492816431144	8.22336332031213
H	1.28386460719213	1.66186325352473	7.49414930081253
H	0.19193066668650	2.74617045998157	8.33783453100888

H	3.47157596299861	-3.25436256421706	-7.01372811865248
C	2.81639668938454	-5.07494262234507	-6.07672274776528
H	0.84886918224945	-5.36891042552010	3.23896266312151
H	2.33893239283052	-5.58932682077026	4.13287599639918
H	4.04800291940923	-6.32808994247300	0.83678893312155
C	4.80402695687393	-6.28102943266928	2.84801335399658
H	1.48912021373117	6.01200641437813	-4.65759415424828
H	0.09115566853852	5.69979706798677	-3.64931726726631
H	3.37006743456856	6.98235522840832	-1.52145952523652
C	3.96756725560514	6.94932843599191	-3.58570700056934
H	2.92795073514194	1.92846212269318	7.21561500261929
H	1.82818347072676	2.76735332164382	8.27279608207735
H	-0.39831506735264	3.63581180277482	5.49813199214529
C	-0.42841914934269	3.96369863096564	7.59996145237129
C	-0.69614360501029	-2.23372985905785	-7.28098088634810
H	2.26405210048888	-4.12293709794382	-7.91978776714584
C	1.37565998651481	-7.41166371092078	3.52267523164216
H	3.41536047970498	-7.58608472546174	1.86007259751936
C	0.42541091087099	7.76841401283125	-4.02112543706976
H	2.55368407658898	8.15630906814994	-2.51430575326222
C	3.23511262262221	4.05429212472441	7.28847033234167
H	0.78166860431000	4.81135678855007	6.03658632006299
H	3.20269609977324	-4.82612753206936	-5.08661614150333
H	3.56571285148318	-5.70559109453797	-6.55654385825852
H	1.91086350845762	-5.66970002102510	-5.95629899519060
H	4.49946323034758	-6.52148753411385	3.86556674660673
H	5.14062533568173	-5.24544496478065	2.83382854815413
H	5.66176915089462	-6.90816129072699	2.60587931749982
H	3.56460821101177	7.14025137631419	-4.57920979562776
H	4.39290946018085	5.94693696078841	-3.58197784322074
H	4.78410605624785	7.65251290069111	-3.42305896909254
H	0.20469964753878	4.31317033436787	8.41328372849883
H	-0.85599907181415	3.00417727096454	7.88899161417172
H	-1.24280080380428	4.67860789011253	7.49090754212846
H	-0.27644540186389	-1.98987219192522	-8.25663152902492
H	-0.73014147539014	-1.32121760611789	-6.69163442017761
H	-1.72248632276289	-2.57742884837603	-7.42229493577214
H	2.24099749504908	-8.04564822030716	3.70535311375325
H	0.82950009993846	-7.81757195712837	2.67180770889700
H	0.73357031294253	-7.47560507818837	4.39994551434756
H	1.21728467788839	8.46427985225034	-4.29116208806092
H	-0.08346120576634	8.15672858777043	-3.13953344739736
H	-0.28779835157681	7.75165051936615	-4.84399976799926
H	2.67685013847433	4.97891580928829	7.42713094766863
H	3.79385967579141	4.13511729165241	6.35723068117639
H	3.95939536809137	3.98434725282941	8.09978265251284

H	3.51819600634863	-1.14515324279464	-6.94507034768475
C	4.28101829442945	-3.14986292947922	-6.82138095156750
H	0.42463336401234	-6.27670212948335	2.32908150347639
H	1.60180610843461	-7.15917063633200	3.27435344089631
H	4.25422448607127	-6.12877990241287	0.80797185158449
C	4.36005663501274	-7.21330822324562	2.66084544239923
H	3.41738179087803	6.88519537553657	-3.88384086542009
H	3.78348317125279	5.21776764521924	-4.27627577281425
H	-0.19847167902082	5.90313769838568	-3.44005801433927
C	0.79682315632787	7.80129637356947	-3.32477506550242
H	3.98759279744366	1.80808151603233	6.85429553995403
H	3.10160944475195	2.80874665811961	7.97974008513256
H	1.46120157766633	4.54313067538299	5.49521049657907
C	-0.12304651546799	3.34468573693685	6.32638908840550
C	-0.25431248026601	-3.33698822991202	-6.14377424429151
H	2.73670708862650	-2.27171396941213	-8.02762061824949
C	0.93343733308879	-8.17645251494528	1.49334923499585
H	3.45043040119692	-7.67367424970483	0.76885798011513
C	2.88140145694627	6.29593638501020	-5.88463911217604
H	0.40103163209536	6.47594654942014	-4.97106192370938
C	4.41633839770233	3.90616703020827	6.68430662310174
H	1.57333380362836	4.27363806581195	7.21611217111126
H	4.76665560974936	-3.02075005262823	-5.85534529912191
H	5.03745756583502	-3.00157854182112	-7.59275566904060
H	3.93769867193266	-4.18170062845680	-6.88918776945702
H	3.79249760666131	-7.93577956327707	3.24433701175587
H	4.58081549536894	-6.35929870169648	3.29887341369383
H	5.30824433699236	-7.67893623265800	2.39642359632663
H	1.58868733905948	8.36703742937028	-3.81181422634485
H	1.00522369616034	7.77459896361253	-2.25672786809702
H	-0.13214142901131	8.35290470486333	-3.46013710307984
H	-0.27903524083936	2.64011450620425	7.14286034250181
H	-0.43404866459813	2.86322476528187	5.40404331060019
H	-0.77127732632181	4.20720090941771	6.49090900973810
H	-0.60092246711513	-2.63607382344298	-6.90273418623898
H	-0.55537835421767	-2.95391929764829	-5.17331291118613
H	-0.75982850432624	-4.29101458160769	-6.30276674204242
H	1.73079155500479	-8.91665250537629	1.50468468047377
H	0.68992099660456	-7.94966307211670	0.45646079628759
H	0.05792440723711	-8.63307010392026	1.95141810848665
H	2.25164995482633	7.16286515380669	-6.07103262542869
H	2.45372022051018	5.44270079492721	-6.40939302342568
H	3.85844869826233	6.50285649039741	-6.31756834777501
H	3.91979702665430	4.87167929161495	6.77565988214868
H	4.85710644727913	3.84748055518754	5.69056776826863
H	5.23122813993605	3.88317879643396	7.40841294041337

3

C	-0.76957974992748	-2.08301393662068	-2.55775383574003
C	0.25266652112633	-2.34740236679502	-3.47110947389411
C	0.75028173610496	-1.35488497161934	-4.32321182017853
C	0.20415985237863	-0.06950864762263	-4.25556088712264
C	-0.85028660021532	0.21841925773808	-3.37916498977256
C	-1.28226575796272	-0.79508708504333	-2.53241156023940
O	0.78861835667410	-3.60494711279258	-3.51774878324429
O	0.74185327620421	0.87238659363928	-5.08838759805449
H	-2.07463910415903	-0.56994009401305	-1.83291889867610
C	-1.51409796261814	1.58246604989145	-3.35339800489304
C	-0.92369719014846	2.45946358131141	-2.27063538259758
C	0.17956635886765	3.26426520276328	-2.55769049617518
C	0.79023412352752	4.05164377987058	-1.57531599243505
C	0.26217722334763	4.05395249774893	-0.28125351466431
C	-0.86914999831672	3.28531402757495	0.03356362409292
C	-1.40814043905706	2.48878350015759	-0.97041855600426
O	0.68236636301655	3.28214250915137	-3.82924746179247
H	-2.25684385054386	1.86737365365167	-0.72762173310265
C	-1.45792294229690	3.30435470746149	1.43066838677795
C	-0.79129590861235	2.26818798925216	2.30929835182208
C	0.24746116103228	2.62596975798316	3.16633686925233
C	0.89433339737708	1.68077106542958	3.97079799919286
C	0.46098877087049	0.35310288753853	3.93824483283049
C	-0.61242890866471	-0.02988835480459	3.12149262852632
C	-1.17868209761790	0.93687526330182	2.30237261405876
O	0.65115916646612	3.93267119996717	3.21492139816100
O	1.11796909768509	-0.53322191778230	4.74759783243850
H	-1.98979181492210	0.64465092701035	1.65034790590826
C	-1.15508874190221	-1.44459627235186	3.13701061809654
C	-0.55452228984831	-2.26660898710834	2.01917693882525
C	0.61934731412521	-2.98760443688045	2.24217813630169
C	1.22798409880017	-3.73344142674267	1.22712667329378
C	0.62804042605124	-3.77927492090293	-0.03467400655281
C	-0.56875514437291	-3.09227495968601	-0.28307166923355
C	-1.10456553592009	-2.33071408360894	0.74774278606949
O	1.19149168261202	-2.96691884319165	3.48465765884088
O	1.25746911664075	-4.53474387280801	-0.98395258012282
H	-2.01433030471065	-1.78240208233639	0.55190986635411
C	-1.25717324327117	-3.17539766866709	-1.62964874869820
C	-3.04110151103569	1.49353490100745	-3.29113716048242
C	-2.98493609642710	3.19803538022007	1.44631962894145
H	-1.22385782242665	4.27817304622238	1.85438680123073
C	-2.68469634825422	-1.49682272210014	3.16618640530866
H	-0.82391187971649	-1.90611191809780	4.06387535602437
C	-2.78448874599344	-3.22533211869540	-1.53710958216934
H	-0.95913909946285	-4.11732995409919	-2.08336532047144
C	-3.68677635158724	0.69732811441943	-4.42289365317512
H	-3.36029559743135	1.05854256044389	-2.34418770635655
H	-3.43623428538094	2.51286195355264	-3.30081266712576
H	-3.38571634472329	3.98280885416110	0.79939435562516
H	-3.30701320000604	2.25455153485649	1.00617537116345
C	-3.62052790431022	3.31458330578972	2.82998724424306
C	-3.33757969955677	-0.74619543348796	4.32445731258501
H	-3.10103775768596	-1.11366003200349	2.23474107616519
H	-2.97893924782996	-2.54897171269434	3.21101325019798
C	-3.35300994271424	-4.34672792573943	-0.67053406960941
H	-3.18092637102469	-2.27660037393247	-1.17523020541918
H	-3.16759980593822	-3.33461220119838	-2.55530274483234
H	-1.28433028673278	2.07255036544432	-4.29667385740916
C	2.05928795035878	2.11546392310103	4.80939227641047
C	2.52828235839219	-4.42560914347674	1.51167415081688
H	1.28346451608822	4.13334152116591	-3.86951754426470
H	1.18717290266561	4.01285906909728	4.09998360727212
H	1.09249658595438	-1.43323731677164	4.35543332787787
H	1.82751823984613	-3.78865697804082	3.49998610277346
H	1.02271312895765	-4.23897000540555	-1.89059997378787
H	1.32850997973320	-3.63482592991959	-4.40818682036638
O	0.89937231108983	4.84845378101859	0.63065919166756
C	1.90817993934135	-1.66523520564492	-5.22679977675020
H	0.66158326487548	1.76582758181702	-4.68577695163750
C	2.03614839451562	4.81536660022953	-1.91840668831277
H	0.73383173731691	4.55597453724087	1.55193829953195
N	1.82125913585309	-3.03553179043079	-5.77077879776187
N	1.94362019300669	5.42327926679422	-3.26277383631614
N	1.79621342350058	3.42257451683191	5.44771340150927
N	2.51443313647569	-5.05940590935994	2.84767237010158
H	2.32751697461534	1.34981659196655	5.53446075557834
H	2.93164313700555	2.25904212199622	4.16781548540958
H	2.77653266950106	-5.13389692944150	0.72401557379378
H	3.33139787817155	-3.68582369133187	1.53281347937375
H	2.00339257692799	-0.92252447926798	-6.01703705923489
H	2.83438270844349	-1.61829586398733	-4.64927789316955
H	2.27017278571664	5.55082469347135	-1.15167517582882
H	2.87893679361254	4.12149965397266	-1.95278960528460
C	-5.12500723898516	3.10398469714471	2.72846764198501
C	-3.30232765157782	4.63597465035926	3.51531887353955
C	-4.85365594623727	-0.82750192132957	4.20790746202533
C	-2.86918023409887	-1.24405975497632	5.68469471301989
C	-4.87440524918626	-4.27721780423566	-0.66829062485366
C	-2.87076153661539	-5.72336312840807	-1.10645274063014
C	-5.19358785855218	0.62463610950396	-4.21412078477025
C	-3.35622101669526	1.25994255069735	-5.79819621916453
H	-3.29505067124044	-0.32199489920536	-4.37655922492740
H	-3.21184181226562	2.51121084075731	3.44749595879940
H	-3.05238104005168	0.30523967810323	4.24059946939801
H	-3.01016325656281	-4.18297344915684	0.35392370510892
H	-5.58726877662551	3.88533717028135	2.12213263170188
H	-5.35982419214114	2.14418537878465	2.26795328402842
H	-5.59553675204814	3.12611099899917	3.71156039664924
H	-2.23716705597635	4.75336479504251	3.70549135138438
H	-3.62781506015152	5.47952752968003	2.90317076480626

C	-0.509534000	-2.347401000	-2.337371000
C	0.435717000	-2.743923000	-3.284720000
C	0.894958000	-1.884437000	-4.280779000
C	0.418293000	-0.565649000	-4.287181000
C	-0.561862000	-0.142560000	-3.384467000
C	-0.979532000	-1.046882000	-2.417223000
O	0.883861000	-4.048573000	-3.188668000
O	0.925462000	0.261674000	-5.246802000
H	-1.725483000	-0.725950000	-1.704393000
C	-1.212112000	1.223842000	-3.496650000
C	-0.636320000	2.199675000	-2.492729000
C	0.489856000	2.959655000	-2.813198000
C	1.076260000	3.842828000	-1.907141000
C	0.506401000	3.958430000	-0.630431000
C	-0.646217000	3.243555000	-0.280834000
C	-1.171029000	2.370264000	-1.225024000
O	0.995318000	2.801474000	-4.088013000
H	-2.048629000	1.801622000	-0.960270000
C	-1.282864000	3.419076000	1.083717000
C	-0.649800000	2.503311000	2.111796000
C	0.273124000	2.982271000	3.040353000
C	0.844134000	2.162636000	4.013956000
C	0.474799000	0.810291000	4.037834000
C	-0.485662000	0.303131000	3.155803000
C	-0.995460000	1.164734000	2.195147000
O	0.590460000	4.323341000	2.946976000
O	1.078119000	0.027362000	4.980089000
H	-1.729288000	0.783756000	1.498981000
C	-1.010687000	-1.114616000	3.277245000
C	-0.372402000	-2.035427000	2.260394000
C	0.805881000	-2.720103000	2.565634000
C	1.436333000	-3.561483000	1.650151000
C	0.855375000	-3.716596000	0.382118000
C	-0.346315000	-3.081733000	0.052021000
C	-0.910701000	-2.239078000	1.000486000
O	1.313780000	-2.530809000	3.835525000
O	1.519757000	-4.530254000	-0.486067000
H	-1.831498000	-1.735727000	0.748851000
C	-1.012044000	-3.317171000	-1.287249000
C	-2.741462000	1.139282000	-3.447954000
C	-2.809413000	3.293739000	1.063077000
H	-1.083278000	4.442489000	1.390228000
C	-2.541547000	-1.167442000	3.255960000
H	-0.724005000	-1.484274000	4.257586000
C	-2.542475000	-3.343695000	-1.224142000
H	-0.726598000	-4.311836000	-1.618290000
C	-3.370396000	0.213192000	-4.486913000
H	-3.076159000	0.818615000	-2.461926000
H	-3.134293000	2.150269000	-3.584091000
H	-3.194991000	3.990418000	0.314439000
H	-3.106600000	2.299832000	0.729556000
C	-3.493323000	3.564777000	2.401488000
C	-3.231194000	-0.319961000	4.322442000
H	-2.922166000	-0.861224000	2.282051000
H	-2.840143000	-2.212039000	3.378463000
C	-3.143509000	-4.367266000	-0.263590000
H	-2.935080000	-2.358540000	-0.972558000
H	-2.902104000	-3.553400000	-2.235070000
H	-0.976385000	1.613307000	-4.483126000
C	1.790836000	2.683344000	5.053341000
C	2.694743000	-4.313782000	1.968085000
H	1.807118000	3.316755000	-4.188423000
H	1.305749000	4.535335000	3.562235000
H	1.106284000	-0.893639000	4.658025000
H	2.148288000	-3.009428000	3.931588000
H	1.219432000	-4.385703000	-1.403786000
H	1.633556000	-4.178601000	-3.785961000
O	1.132882000	4.813314000	0.225830000
C	1.796940000	-2.337062000	-5.389242000
H	0.886818000	1.181644000	-4.921967000
C	2.289797000	4.659161000	-2.243007000
H	0.855177000	4.662665000	1.149095000
N	1.078030000	-2.561063000	-6.637867000
N	1.979891000	6.047655000	-2.574081000
N	1.135193000	2.905676000	6.339563000
N	2.454405000	-5.725313000	2.239372000
H	2.577526000	1.948626000	5.209938000
H	2.308523000	3.590010000	4.690875000
H	3.357208000	-4.254598000	1.106877000
H	3.249433000	-3.822393000	2.788210000
H	2.542725000	-1.566522000	-5.576298000
H	2.371179000	-3.229153000	-5.083408000
H	2.945984000	4.669762000	-1.375620000
H	2.876428000	4.168641000	-3.040617000
C	-4.991903000	3.326202000	2.274002000
C	-3.210675000	4.961464000	2.936781000
C	-4.742454000	-0.416153000	4.162979000
C	-2.810753000	-0.696115000	5.736685000
C	-4.663657000	-4.285184000	-0.311496000
C	-2.665737000	-5.785000000	-0.544741000
C	-4.879470000	0.160403000	-4.288221000
C	-3.023730000	0.610989000	-5.914568000
H	-2.978965000	-0.792157000	-4.314634000
H	-3.103797000	2.844425000	3.124929000
H	-2.942721000	0.720535000	4.155578000
H	-2.828642000	-4.105039000	0.749222000
H	-5.442151000	4.034980000	1.576526000
H	-5.202528000	2.321140000	1.908098000
H	-5.493017000	3.442648000	3.234896000
H	-2.154777000	5.114641000	3.150775000
H	-3.519327000	5.722419000	2.217288000

C	-0.246761000	-2.210986000	-2.084317000
C	0.884612000	-2.502620000	-2.861569000
C	1.379895000	-1.581129000	-3.790846000
C	0.772241000	-0.323044000	-3.889205000
C	-0.357309000	-0.003746000	-3.128213000
C	-0.818827000	-0.958452000	-2.234208000
O	1.520745000	-3.711441000	-2.785183000
O	1.319649000	0.549395000	-4.786704000
H	-1.695534000	-0.721173000	-1.650523000
C	-1.082663000	1.314164000	-3.298331000
C	-0.562237000	2.355417000	-2.332098000
C	0.405864000	3.267327000	-2.752310000
C	0.916952000	4.250650000	-1.891538000
C	0.417043000	4.308363000	-0.591784000
C	-0.538892000	3.403141000	-0.125155000
C	-0.991325000	2.437618000	-1.014365000
O	0.863219000	3.202920000	-4.036197000
H	-1.716559000	1.718013000	-0.663431000
C	-1.038938000	3.475146000	1.304524000
C	-0.313350000	2.493668000	2.206791000
C	0.823762000	2.875790000	2.931904000
C	1.436538000	2.002768000	3.835364000
C	0.928620000	0.706229000	3.970026000
C	-0.201307000	0.286598000	3.257533000
C	-0.776474000	1.197482000	2.382126000
O	1.357216000	4.132395000	2.831606000
O	1.583118000	-0.113603000	4.846671000
H	-1.660614000	0.895501000	1.840099000
C	-0.789605000	-1.096662000	3.459837000
C	-0.230488000	-2.095634000	2.466324000
C	0.818483000	-2.948067000	2.819625000
C	1.355861000	-3.872877000	1.924656000
C	0.806846000	-3.934297000	0.633272000
C	-0.250907000	-3.112138000	0.247952000
C	-0.727669000	-2.197787000	1.175022000
O	1.302257000	-2.823762000	4.109967000
O	1.308727000	-4.825932000	-0.266995000
H	-1.535392000	-1.542713000	0.881498000
C	-0.838498000	-3.236830000	-1.139511000
C	-2.608304000	1.183226000	-3.253083000
C	-2.564987000	3.367520000	1.389900000
H	-0.807005000	4.473408000	1.663757000
C	-2.321075000	-1.110868000	3.489584000
H	-0.487664000	-1.435038000	4.447015000
C	-2.369230000	-3.239581000	-1.141042000
H	-0.546504000	-4.213607000	-1.515946000
C	-3.206686000	0.191211000	-4.248240000
H	-2.943649000	0.912812000	-2.251589000
H	-3.025493000	2.175273000	-3.446434000
H	-2.985494000	4.116480000	0.713646000
H	-2.904599000	2.403510000	1.011484000
C	-3.151753000	3.573183000	2.784218000
C	-2.956133000	-0.229068000	4.561606000
H	-2.728258000	-0.824589000	2.520258000
H	-2.636672000	-2.145569000	3.648608000
C	-3.015328000	-4.314246000	-0.268951000
H	-2.758772000	-2.271403000	-0.827511000
H	-2.697901000	-3.372434000	-2.175214000
H	-0.851876000	1.681090000	-4.294933000
C	2.611782000	2.428457000	4.663933000
C	2.512335000	-4.770868000	2.277767000
H	1.295797000	4.121233000	-4.217517000
H	1.106688000	4.538979000	1.981755000
H	1.406576000	-1.047383000	4.630502000
H	2.117237000	-3.328121000	4.217080000
H	1.840756000	-5.511769000	0.308469000
H	1.377601000	-4.127913000	-1.910573000
O	0.851160000	5.262637000	0.310446000
C	2.505745000	-1.937146000	-4.713085000
H	1.088742000	1.471905000	-4.560776000
C	2.001507000	5.156964000	-2.401603000
H	1.590419000	5.762272000	-0.056725000
N	2.045751000	-2.259402000	-6.070412000
N	1.728376000	5.605246000	-3.777376000
N	2.268602000	2.713461000	6.060773000
N	2.380316000	-6.095340000	1.645851000
H	3.334595000	1.614182000	4.678012000
H	3.097516000	3.283551000	4.184031000
H	3.442803000	-4.332876000	1.912346000
H	2.624577000	-4.882241000	3.361478000
H	3.177157000	-1.084157000	-4.803856000
H	3.077075000	-2.758048000	-4.271017000
H	2.179218000	6.014127000	-1.744222000
H	2.942387000	4.604827000	-2.434069000
C	-4.662157000	3.386164000	2.741506000
C	-2.787983000	4.925777000	3.379614000
C	-4.473371000	-0.305972000	4.462334000
C	-2.485651000	-0.582692000	5.965423000
C	-4.531992000	-4.210708000	-0.362901000
C	-2.545582000	-5.719767000	-0.619161000
C	-4.720216000	0.143920000	-4.086974000
C	-2.821674000	0.501229000	-5.688123000
H	-2.814709000	-0.799521000	-4.007926000
H	-2.737048000	2.801584000	3.436024000
H	-2.659164000	0.802716000	4.363368000
H	-2.731169000	-4.116419000	0.768065000
H	-5.130792000	4.148576000	2.116272000
H	-4.928645000	2.411826000	2.331805000
H	-5.098657000	3.459957000	3.737490000
H	-1.716534000	5.027317000	3.540702000
H	-3.107717000	5.738249000	2.723898000

H -3.81489998244428 4.70627669039485 4.47479385622984	H -3.757898000 5.138714000 3.862619000	H -3.274447000 5.067046000 4.344680000
H -5.19792670700958 -0.44595083213793 3.24645907009201	H -5.055571000 -0.135294000 3.157199000	H -4.820681000 -0.040040000 3.463811000
H -5.19583645446228 -1.86016954245381 4.29824490340936	H -5.088124000 -1.435361000 4.345806000	H -4.825993000 -1.316455000 4.677910000
H -5.34170697749743 -0.24681066840663 4.99074634518660	H -5.255562000 0.238622000 4.867223000	H -4.950031000 0.369652000 5.172407000
H -3.06776166604283 -2.31177467963908 5.79783646044173	H -3.004335000 -1.752853000 5.931504000	H -2.679732000 -1.633943000 6.188234000
H -1.80234224610307 -1.08589686434183 5.83152740279540	H -1.752749000 -0.511825000 5.912471000	H -1.420477000 -0.402990000 6.096924000
H -3.39020084953567 -0.72295909675439 6.48798349999126	H -3.367955000 -0.116103000 6.472301000	H -3.008595000 0.016281000 6.710623000
H -5.22568607368486 -3.30056854616288 -0.33368908185753	H -5.014056000 -3.277656000 -0.084809000	H -4.879806000 -3.212864000 -0.093818000
H -5.27319233872866 -4.44836421993569 -1.66998578636511	H -5.036917000 -4.550436000 -1.302432000	H -4.872829000 -4.417116000 -1.379182000
H -5.30377072086495 -5.03111841374552 -0.00829715244787	H -5.118450000 -4.966797000 0.407169000	H -5.019006000 -4.925253000 0.300766000
H -3.11197085130637 -5.90562463472285 -2.15562721338723	H -2.876667000 -6.070278000 -1.577474000	H -2.727325000 -5.939568000 -1.673138000
H -1.79437337382225 -5.83493087233151 -0.98724205681858	H -1.595626000 -5.897587000 -0.378984000	H -1.482537000 -5.855671000 -0.429390000
H -3.34761266587428 -6.50578848110885 -0.51595698581343	H -3.170729000 -6.498977000 0.105747000	H -3.079909000 -6.464494000 -0.029003000
H -5.43892704872967 0.18962630216210 -3.24469856003705	H -5.134525000 -0.160274000 -3.277637000	H -5.002845000 -0.104310000 -3.063344000
H -5.64290015585145 1.61892466388041 -4.25396602310596	H -5.330320000 1.141429000 -4.449365000	H -5.168449000 1.108990000 -4.330853000
H -5.66996969874753 0.01659974813778 -4.98336384495659	H -5.343683000 -0.535805000 -4.986766000	H -5.163344000 -0.602568000 -4.746212000
H -3.66787638745577 2.30364170046680 -5.87656011001929	H -3.326973000 1.640720000 -6.114149000	H -3.122879000 1.514033000 -5.963663000
H -2.29057111679775 1.21362619972872 -6.01485521946255	H -1.957448000 0.530338000 -6.116446000	H -1.749226000 0.414742000 -5.851427000
H -3.87164500134143 0.70104454075240 -6.57943517913235	H -3.537564000 -0.030902000 -6.629894000	H -3.310612000 -0.189096000 -6.375475000
C 3.12368007522305 -3.49071387098730 -6.26962753378488	C 0.039894000 -3.590205000 -6.565926000	C 1.132434000 -3.402888000 -6.134303000
C 0.69918055219801 -3.20937500194233 -6.71232922802228	C 1.968106000 -2.683512000 -7.786932000	C 3.149759000 -2.332894000 -7.020144000
C 3.87211829217168 -5.37611363262306 3.30436076614584	C 1.713280000 -5.937360000 3.473074000	C 1.297293000 -6.877222000 2.261457000
C 1.55584023004531 -6.17397197733520 2.93243753792853	C 3.651448000 -6.543264000 2.082117000	C 3.663794000 -6.799416000 1.512193000
C 3.26843711783847 5.78109512039057 -3.78207135257246	C 1.228813000 6.154982000 -3.817778000	C 2.934637000 6.153012000 -4.410787000
C 0.94050781345770 6.49973623743470 -3.32625881506186	C 3.153666000 6.913772000 -2.484457000	C 0.532531000 6.460653000 -3.872884000
C 0.70079291744578 3.36085587791746 6.42979662677328	C 2.090233000 3.054006000 7.435205000	C 3.430486000 2.635793000 6.938123000
C 3.03471945671741 4.03585401151957 5.93983437231070	C 0.117947000 3.946684000 6.265556000	C 1.475681000 3.929629000 6.244164000
H 3.82846539531609 -3.36537689341535 -5.44686675686882	H 0.281842000 -4.350198000 -5.812177000	H 1.326565000 -4.112007000 -5.322340000
H 3.48247635306379 -2.84769580639473 -7.08319707598450	H 0.017727000 -4.123013000 -7.517408000	H 1.327909000 -3.948326000 -7.059218000
H -0.20849159569028 -2.96855167134092 -6.15992343739998	H 2.569412000 -1.772196000 -7.837649000	H 3.659025000 -1.365216000 -7.011637000
C 0.74319780359957 -2.38935301284724 -8.00888518128652	C 2.898361000 -3.894407000 -7.826852000	C 4.186992000 -3.436142000 -6.812644000
H 4.44508490411012 -4.45032175080891 3.24192648650288	H 0.846505000 -5.277019000 3.440493000	H 0.430730000 -6.218835000 2.310821000
H 4.35891742699128 -6.08823969863531 2.62681218734648	H 2.295649000 -5.636036000 4.360577000	H 1.546428000 -7.137351000 3.296246000
H 0.59670017233587 -5.78673953744877 2.58998393418077	H 4.046012000 -6.341067000 1.084017000	H 4.332547000 -6.131227000 0.968498000
C 1.89568498495127 -7.43360494990727 2.14934901475826	C 4.770809000 -6.360792000 3.104573000	C 4.332347000 -7.250527000 2.802629000
H 3.75628136218797 6.52165521282530 -3.13689878384124	H 1.801738000 5.768784000 -4.679458000	H 3.293873000 7.036945000 -3.869973000
H 3.87691873339805 4.87757436068241 -3.72944140023110	H 0.358997000 5.505812000 -3.719915000	H 3.710104000 5.392790000 -4.312010000
H 0.01340578918223 6.08464725954176 -2.93168788467096	H 3.572499000 6.780017000 -1.484833000	H -0.283356000 5.914260000 -3.401187000
C 1.26953519578099 7.78813951092018 -2.58724300324138	C 4.260719000 6.713328000 -3.516874000	C 0.631273000 7.847549000 -3.255427000
H -0.15693600469576 2.92909229295570 5.91518894324425	H 2.748114000 2.182899000 7.405395000	H 3.846876000 1.628758000 6.848103000
H 0.42300106985795 4.38310720943258 6.68057551754680	H 1.539495000 2.981825000 8.373584000	H 3.073969000 2.727198000 7.966864000
H 3.72425926734073 4.06133649346752 5.09526424449103	H -0.547216000 3.683575000 5.444357000	H 1.750502000 4.696419000 5.509118000
C 2.85215497806171 5.44499039761879 6.46773796548356	C -0.719267000 4.090377000 7.522502000	C -0.032901000 3.708822000 6.200959000
C 3.14529075291029 -4.93919368415830 -6.71773340894665	C -1.354122000 -3.036139000 -6.299872000	C -0.339620000 -3.008532000 -6.123140000
H 0.64239328994236 -4.27071747403029 -6.93496742160040	H 1.344711000 -2.687429000 -8.683688000	H 2.717821000 -2.441311000 -8.018067000
C 3.93386257344326 -5.89555329756317 4.72750374437091	C 1.211183000 -7.359449000 3.658654000	C 0.925700000 -8.121793000 1.480797000
H 1.42582095212350 -6.42060377610840 3.98492379498028	H 3.349011000 -7.591269000 2.075831000	H 3.500932000 -7.659738000 0.865731000
C 3.25099362232624 6.27679940526116 -5.21482632324936	C 0.741295000 7.555483000 -4.137062000	C 2.754062000 6.484159000 -5.878658000
H 0.75402510191652 6.71726952933513 -4.37668187918089	H 2.813911000 7.948719000 -2.529283000	H 0.272826000 6.545157000 -4.926291000
C 0.96365767632865 2.57370820910314 7.70544506254826	C 2.942856000 4.320369000 7.462036000	C 4.549348000 3.652114000 6.713672000
H 3.50675351680749 3.40855015821038 6.70595842488352	H 0.550319000 4.927938000 6.003542000	H 1.722972000 4.342894000 7.223710000
H 0.48641579626697 -3.04480108688450 -8.84218863047160	H 3.570541000 -3.875071000 -6.967244000	H 4.684370000 -3.300580000 -5.851117000
H 1.76461863837895 -2.05913440531802 -8.20445621989995	C 3.716477000 -3.933312000 -9.106681000	C 5.224759000 -3.451304000 -7.922397000
C -0.19203417542639 -1.18946515610590 -8.01243648455742	H 2.309201000 -4.809518000 -7.738371000	H 3.683366000 -4.403869000 -6.765434000
C 0.79048729160253 -8.46951107340786 2.27357377458836	H 4.400532000 -6.594759000 4.104156000	H 3.677708000 -7.939705000 3.337438000
H 2.83441600204316 -7.85704393363799 2.50970828694094	H 5.085295000 -5.315681000 3.127732000	H 4.486702000 -6.393619000 3.460252000
H 2.04761418773007 -7.18914675571100 1.09763262165467	C 5.965132000 -7.244546000 2.785952000	C 5.664450000 -7.927906000 2.526631000
C 0.12841166964567 8.78646988777331 -2.69265857540007	H 3.859121000 6.850150000 -4.522395000	H 1.422788000 8.419189000 -3.741811000
H 2.17937055849905 8.23168727396368 -2.99444833011829	H 4.632939000 5.688435000 -3.470146000	H 0.905414000 7.769879000 -2.202240000
H 1.47046416912837 7.57517198520835 -1.53687418587317	C 5.409882000 7.682353000 -3.295246000	C -0.685561000 8.596565000 -3.377876000
H 2.25490928719902 5.43334014441403 7.37991338775223	H -0.114169000 4.482091000 8.341164000	C -0.545395000 2.803358000 7.305684000
H 2.29150369312495 6.02806175579053 5.73420210003819	H -1.059594000 3.099661000 7.830710000	H -0.319388000 3.302869000 5.233341000
C 4.18667389513385 6.11325582256312 6.75425214876355	C -1.912545000 5.005156000 7.299883000	H -0.511032000 4.689664000 6.274026000
H 2.54803009698204 -5.06429304388833 -7.62108758094803	H -1.632680000 -2.389862000 -7.134671000	H -0.541762000 -2.398370000 -7.006321000
H 2.68163642884941 -5.55800102353715 -5.94673460863190	H -1.331022000 -2.400163000 -5.418592000	H -0.540637000 -2.376087000 -5.261407000
C 4.56260527187826 -5.41778703328343 -6.98767097289435	C -2.386554000 -4.132965000 -6.109485000	C -1.257312000 -4.218889000 -6.093508000
H 3.45489998176752 -6.87264115854803 4.79400320576520	H 2.051112000 -8.044682000 3.785870000	H 1.738346000 -8.848310000 1.515436000
H 3.36641491009391 -5.22394548568064 5.37499250531868	C 0.315255000 -7.835420000 2.528184000	H 0.788234000 -7.853156000 0.431828000
C 5.36716626789937 -6.00756245989981 5.22044331749774	H 0.662365000 -7.387167000 4.602244000	C -0.342837000 -8.760524000 2.021908000
H 2.73397868731187 7.23458950578190 -5.27725704741243	H 1.586293000 8.214685000 -4.338715000	H 2.063880000 7.319953000 -5.995397000
H 2.68176194803953 5.57365092275672 -5.82628029938572	H 0.233237000 7.958652000 -3.258650000	H 2.298536000 5.628877000 -6.381642000
C 4.65732690988629 6.43063839227690 -5.77027320520257	C -0.195907000 7.565939000 -5.333415000	C 4.077170000 6.832805000 -6.539892000
C -0.27077572402836 2.54244774947425 8.59155558521644	H 2.296950000 5.199320000 7.497147000	H 4.142168000 4.663097000 6.780541000
H 1.79656369563518 3.01548777067221 8.25440018606090	H 3.523506000 4.399935000 6.541323000	H 4.944930000 3.545213000 5.702278000
H 1.25802769372397 1.55328400993387 7.45842744290444	C 3.880585000 4.334201000 8.657475000	C 5.674245000 3.485263000 7.721159000
H -1.22503853962624 -1.50262721785634 -7.85785337465121	H 4.331367000 -3.038028000 -9.207659000	H 5.754898000 -2.499405000 -7.978838000
H -0.14707716422550 -0.65815980922967 -8.96310461403406	H 3.071327000 -3.987842000 -9.984524000	H 4.759787000 -3.623851000 -8.894123000
H 0.05389854851897 -0.48104722381667 -7.22507518116517	H 4.382432000 -4.795097000 -9.131551000	H 5.967834000 -4.233246000 -7.768346000
H 1.03205638214296 -9.37562210955444 1.71987611408153	H 6.377609000 -7.008205000 1.804317000	H 6.355122000 -7.250299000 2.024029000
H -0.15318941284909 -8.08509618003762 1.88553637449875	H 5.682828000 -8.298202000 2.777478000	H 5.536029000 -8.801439000 1.886853000
H 0.62852724224747 -8.75077034982889 3.31476253737685	H 6.762367000 -7.122489000 3.518267000	H 6.138190000 -8.259896000 3.449035000
H -0.78792343154766 8.38288059230056 -2.26128185893073	H 6.199841000 7.543576000 -4.032486000	H -1.489318000 8.063828000 -2.869206000
H -0.08024094261979 9.03848661080491 -3.73300370500815	H 5.852532000 7.550073000 -2.307139000	H -0.976961000 8.713416000 -4.422073000
H 0.36197036760071 9.71206695397139 -2.16855349448679	H 5.070541000 8.716660000 -3.365718000	H -0.617507000 9.591261000 -2.940136000
H 4.79406704876479 6.18782545075653 5.85200133352435	H -2.573127000 4.610857000 6.527286000	H -0.316876000 3.213356000 8.291298000
H 4.75961154144407 5.55165357570051 7.49271658429648	H -1.595566000 5.999836000 6.984293000	H -0.088483000 1.817492000 7.244138000
H 4.04523908158880 7.12024017379554 7.14357420723352	H -2.498166000 5.120271000 8.210973000	H -1.625789000 2.673577000 7.242474000
H 5.17709115628525 -5.36350299946669 -6.08893014720183	H -3.379528000 -3.715920000 -5.945023000	H -1.097242000 -4.805699000 -5.187981000
H 5.04449063846290 -4.81071865986794 -7.75454303875968	H -2.142512000 -4.753192000 -5.245798000	H -1.081353000 -4.876112000 -6.946761000
H 4.56838100004120 -6.45056610432957 -7.33237666676482	H -2.442260000 -4.788587000 -6.979940000	H -2.306029000 -3.923626000 -6.113900000
H 5.86396089391748 -5.03724620917252 5.21596751229342	H 0.849103000 -7.850329000 1.580053000	H -0.229948000 -9.038643000 3.070126000
H 5.94965326662724 -6.67994346363210 4.59002564587934	H -0.542558000 -7.172558000 2.407057000	H -0.595421000 -9.662402000 1.466764000
H 5.40303373384489 -6.39478665926485 6.23743233983875	H -0.062284000 -8.839584000 2.719315000	H -1.189234000 -8.077536000 1.949879000
H 5.18881253062576 5.47888952296986 -5.77013120629432	H -1.079711000 6.954703000 -5.148764000	H 4.775948000 5.997499000 -6.492111000
H 5.24100174193266 7.13522596328191 -5.17722407356839	H 0.295853000 7.173146000 -6.224058000	H 4.549540000 7.687278000 -6.054425000
H 4.63638039990999 6.79942236019040 -6.79452080627290	H -0.532765000 8.576105000 -5.562038000	H 3.934997000 7.086918000 -7.588934000
H -1.11050014035612 2.07375063973727 8.07754100676384	H 4.560847000 3.481766000 8.635571000	H 5.311906000 3.618778000 8.741536000
H -0.57890204903855 3.54938583997733 8.87538240843581	H 3.323348000 4.284027000 9.593848000	H 6.472651000 4.208462000 7.557436000
H -0.08781006798060 1.98189636121282 9.50709669932979	H 4.486273000 5.239369000 8.681887000	H 6.114027000 2.488765000 7.658792000
	**4**	
C -0.74345130186836 -2.05822316437417 -2.50988028182078	C -0.60675719260554 -2.44230732092281 -2.26744719909913	C -0.55741354210277 -2.24637463578305 -1.97546647218058
C 0.32555690703601 -2.29363725869114 -3.37298159572587	C 0.39557698346777 -2.83641735405771 -3.15297898456289	C 0.60228500620222 -2.58104229849431 -2.69373872521852
C 0.84578955177986 -1.28937253277333 -4.19486163157454	C 0.92718540989911 -1.97604380114001 -4.11153112366209	C 1.18186640939008 -1.67897470185033 -3.59345133135534
C 0.26327779938734 -0.01945297349699 -4.16520827344271	C 0.39965780775106 -0.68201400531701 -4.20534875413700	C 0.61159170172741 -0.40751684333140 -3.74237592948052
C -0.83752787966827 0.24205854326931 -3.33623626800502	C -0.64327476873445 -0.26202012138128 -3.37137256233020	C -0.54408285343215 -0.04507581244869 -3.04169715604551
C -1.28351724447685 -0.78046763933210 -2.50799787710924	C -1.09128135488731 -1.15013266928366 -2.40221858842493	C -1.07821472882885 -0.97634397555360 -2.16270610489314
O 0.88668657726233 -3.53890713574241 -3.42485791257493	O 0.83979836293315 -4.13977921010543 -3.04928137276645	O 1.19974737290193 -3.80511750696075 -2.57852804714530
O 0.80539450953973 0.92857670760149 -4.98771897492525	O 0.93327967653399 0.12567701701623 -5.16766087381362	O 1.24149364129245 0.44092490130638 -4.60814538504593
H -2.11230031274892 -0.57345436015889 -1.84671577288954	H -1.87913570944922 -0.82666352117996 -1.73740574201477	H -1.97030753237270 -0.70356418394388 -1.61972234264160
C -1.52807018939673 1.59220844614499 -3.33544783785768	C -1.29546473476202 1.09746840895206 -3.53963438885924	C -1.20303527311368 1.30252527505220 -3.24253516957793
C -0.96118418432765 2.49450679621038 -2.26074563253875	C -0.73373410567726 2.10833012325489 -2.56154844990888	C -0.65593677525129 2.33637760526617 -2.28276204935146
C 0.10144072722299 3.34715085289359 -2.56368988640483	C 0.36367424660538 2.89641161385078 -2.90935464350449	C 0.33417400622845 3.22048235807960 -2.70785782416965
C 0.68509788192210 4.16724615760499 -1.59141387617546	C 0.92399430111654 3.82509947868190 -2.03239324040801	C 0.84926287070173 4.21653297414931 -1.86631276479005
C 0.18175106703349 4.13715464451356 -0.28765998177259	C 0.36930051883498 3.93918210934352 -0.74955262560190	C 0.34802349793744 4.30042479629839 -0.56932103877406
C -0.90813868789504 3.31760288495990 0.04332552497836	C -0.75972610777163 3.19957767738497 -0.37403809791110	C -0.62417316025231 3.41746531381673 -0.09237887324851
C -1.42640115099231 2.49859514651643 -0.95350906778074	C -1.26498320042768 2.29006082203021 -1.29352025442002	C -1.09135131924655 2.44710373609827 -0.96878487112925
O 0.59350071026397 3.38156573004169 -3.83674814490126	O 0.85274085949118 2.73423812623298 -4.19117319036489	O 0.81014002229442 3.12673995387403 -3.98127151034004
H -2.24146082643077 1.83787774596469 -0.69771245794787	H -2.12190263065595 1.69912451084619 -1.00996418056374	H -1.83166586315680 1.74601962731667 -0.61108696984954
C -1.48558735250817 3.31415864024462 1.44543152850874	C -1.39534674268696 3.39074615564259 0.98997340580480	C -1.11873178428969 3.50978778139009 1.33850024561530
C -0.81669406538305 2.26577401200442 2.30724196632816	C -0.75250072112186 2.49637060605592 2.03147066110342	C -0.41918412376639 2.50622767156732 2.23679492677373
C 0.26140822919889 2.60421663362533 3.12171712804794	C 0.20146538338142 2.98679000378091 2.92221212247726	C 0.75549870070292 2.84331921117922 2.92492658357558
C 0.90992366424477 1.65499647898801 3.91848266769312	C 0.79293230946606 2.18463167962242 3.89857461840858	C 1.36807285020475 1.94513031290839 3.80418302550689
C 0.45141282114434 0.33553359587318 3.90875209622554	C 0.42559461418499 0.83245867704574 3.94980420319328	C 0.81067637291301 0.67076308693184 3.96039327885405
C -0.65531236697764 -0.03169035611675 3.12883058568743	C -0.57548332157894 0.31858444711640 3.11838611611698	C -0.36322974753373 0.29988647167812 3.29396093575618
C -1.23195789767935 0.94227665084142 2.32495554259024	C -1.11286622074034 1.16501864019077 2.15979853720066	C -0.92948260077488 1.23133352549404 2.43375356027635
O 0.70430082963287 3.89673358372130 3.14830823078841	O 0.51435168562197 4.32753067700315 2.79903796264591	O 1.34240268816725 4.07371609090748 2.79783439254199
O 1.12012306802083 -0.55559413335420 4.70289575246972	O 1.06547075710720 0.06127649219423 4.87514012236124	O 1.47014528551337 -0.17845808259417 4.80336234014306
H -2.07216909913777 0.66155471375840 1.70578947660391	H -1.87637869684717 0.77695337453545 1.50081556427389	H -1.84222852590666 0.96304196865067 1.92225794068262
C -1.20828480434781 -1.44215622767019 3.15597050847017	C -1.10416096138411 -1.09180410525782 3.29129393319367	C -1.00556861943345 -1.05261299636498 3.53356875573994
C -0.60572915240989 -2.28047034494597 2.05102066075717	C -0.47855087037579 -2.05477219719145 2.30543247351859	C -0.51763589128237 -2.09581003551065 2.54817014714231
C 0.53682956519880 -3.03958081252276 2.30015847556396	C 0.66429519696355 -2.77993411395750 2.64679460403580	C 0.48357333558795 -3.00157713544775 2.89665470186603
C 1.14423934487488 -3.80901720981453 1.30356275718078	C 1.26402064260762 -3.68248960588228 1.76866458001286	C 0.94650943204846 -3.98376750133900 2.02045212140424
C 0.58199130639511 -3.82705310335371 0.02469432748652	C 0.70321726863158 -3.83291094707465 0.49161781591452	C 0.37446572585336 -4.04167134074319 0.74113478226055
C -0.58918638311576 -3.10615547757831 -0.25073955851666	C -0.46555152803822 -3.15688110952683 0.12594825463859	C -0.62738072468750 -3.14947531846803 0.35211534553653
C -1.12731195783849 -2.32809940696739 0.76651002144365	C -1.00827166599266 -2.26829692763088 1.04363203135870	C -1.03658901661517 -2.19023421715943 1.26404803675217
O 1.08209722285891 -3.04551705130425 3.55338137110005	O 1.15375740120998 -2.58122441115090 3.92268476176892	O 1.00407271291969 -2.89040231596579 4.17759034604260
O 1.21575755438746 -4.59161743488403 -0.91555604438260	O 1.34699060801270 -4.69264988431276 -0.34617253740315	O 0.78953267325960 -4.98122120627019 -0.15170410430026
H -2.01454555646943 -1.75134079223828 0.54856413634341	H -1.90319803140794 -1.73279391781488 0.76571673499160	H -1.80067672048441 -1.48776915581695 0.96347137446951
C -1.24924884687751 -3.16665397651881 -1.61275052322830	C -1.12900212903759 -3.39826881709367 -1.21477865783028	C -1.21901011138618 -3.23804428587394 -1.03827590220847
C -3.05412067288956 1.48405171369568 -3.28048388863031	C -2.82526459120712 1.02336019423649 -3.50947498187789	C -2.73359298028708 1.25125482005249 -3.22253658904177
C -3.01312971775959 3.21272322002724 1.46279943991095	C -2.92101875331908 3.25584249792966 0.96503991793446	C -2.64675024509115 3.45270198626594 1.42493630484347
H -1.24601840309896 4.28096878786454 1.88199158094250	H -1.20358007355164 4.42039527907560 1.28014209956009	H -0.85420002855628 4.50058596840809 1.69638382546554
C -2.73774634345302 -1.49253117266024 3.17552984887547	C -2.63537804868458 -1.14526826334195 3.28232116055279	C -2.53530286688160 -1.00189869838149 3.59827347230853
H -0.88499223248304 -1.89668393092401 4.08916334887076	H -0.81414336484767 -1.42837988917945 4.28256522252165	H -0.69470011314226 -1.38611086366683 4.51994153069338
C -2.77760846510670 -3.22270541497913 -1.54734467487684	C -2.65893512849352 -3.41101464124747 -1.14540701242266	C -2.74649095601850 -3.13854941304324 -1.03697046416061
H -0.93955894602413 -4.09908446519263 -2.07836136122279	H -0.85107470886908 -4.39824744321688 -1.53670293619480	H -0.98599484796552 -4.23008191154364 -1.41657625092157
C -3.68522985110976 0.65734134318942 -4.39838152406797	C -3.44561428905416 0.08836291385116 -4.54523555507972	C -3.36825226607239 0.29088847726671 -4.22567277134377
H -3.37488362828904 1.06822017096678 -2.32539275386942	H -3.17626552205547 0.72245436064512 -2.52290210899633	H -3.09847564976336 1.00263866915059 -2.22570275759108

H	-3.46007084695843	2.49864942182835	-3.31452257845856
H	-3.41224111977294	3.99898732687513	0.81674137446880
H	-3.33728031673276	2.27110274521795	1.02026610758546
C	-3.64589226249574	3.33205220613888	2.84751143441501
C	-3.39820249504682	-0.73562753703469	4.32530117771123
H	-3.14825040140972	-1.11751054822047	2.23807642209477
H	-3.03130445343625	-2.54464069082771	3.22609327109787
C	-3.35360671042948	-4.36218305453557	-0.70992822101099
H	-3.18332285725734	-2.28289101547251	-1.17285538866809
H	-3.14489385775589	-3.31388886968538	-2.57316642731709
H	-1.29941248272023	2.07302718723221	-4.28367684565565
C	2.12602140165318	2.07054119817982	4.69469177931085
C	2.41133406418810	-4.54320963756540	1.63365711358166
H	1.14494051560449	4.28035639370934	-3.87316476641851
H	1.24240679226487	3.96499929649274	4.03649833248533
H	1.04481219082451	-1.46664880055459	4.34546393214472
H	1.64078976193887	-3.92286814038018	3.58358851735651
H	1.03466639863173	-4.26015296907742	-1.82151358739789
H	1.37697647065782	-3.56037918589677	-4.34167468186077
O	0.79823334609191	4.95272705357555	0.62090925630166
C	2.05903808654716	-1.59698159707369	-5.02351951256485
H	0.66829708539946	1.82889762156384	-4.61634315073650
C	1.88312730653049	5.00110720300700	-1.94865217990975
H	0.69446139847174	4.61278357114257	1.53573338414002
N	1.95951542584384	-2.92073581860072	-5.67669516724724
N	1.76735261989282	5.56477615662736	-3.31484535638662
N	1.95018030344298	3.39187954760445	5.33632148285218
N	2.33131850321207	-5.20699416179129	2.95349924282596
H	2.41672824421475	1.29646843092815	5.39718681781832
H	2.95750820992011	2.18020530485647	3.99537880933944
H	2.68084420640158	-5.22524977262993	0.83391142245736
H	3.22321872616211	-3.81704230623439	1.70915412145369
H	2.26237580322933	-0.79421649055539	-5.72468144203685
H	2.92270047379273	-1.65339579651440	-4.35774263388051
H	2.04723511482695	5.77456156316858	-1.20198468879905
H	2.77178707670188	4.36740673423111	-1.93997275068842
C	-5.14490276085533	3.08192582641901	2.75787171458460
C	-3.35917945911234	4.67247367472928	3.50907976726268
C	-4.91333156945370	-0.83215854678061	4.20898094745942
C	-2.92662365663960	-1.21674975927312	5.69056830840728
C	-4.87486854262303	-4.29240371819999	-0.71771987195269
C	-2.86823931520120	-5.72921371093092	-1.17172232110893
C	-5.19385048622884	0.58434590132789	-4.20318487022013
C	-3.34124117577952	1.18683605649370	-5.78343463816375
H	-3.29117985663714	-0.35923572516061	-4.32232994459459
H	-3.21241272926657	2.55006225712499	3.47559774652433
H	-3.12200015538823	0.31731397988306	4.23183659951276
H	-3.01764712655907	-4.22018812180702	0.32022868467962
H	-5.62907034449259	3.83977293649387	2.13887299914926
H	-5.35792217510179	2.10803031293524	2.31691141373284
H	-5.61170341863776	3.11158133867111	3.74257350108862
H	-2.29510998503216	4.83050224162045	3.67131693574533
H	-3.73016021100175	5.49556963047516	2.89493530816839
H	-3.84942633428324	4.73690642793200	4.48055278997494
H	-5.26086847605995	-0.46813964700829	3.24194917213907
H	-5.24600497398913	-1.86655123065466	4.31418598061608
H	-5.40721028677186	-0.24471467510263	4.98307236584693
H	-3.11228460951222	-2.28594835892906	5.81161474401051
H	-1.86211545551127	-1.04418212136313	5.83841737939498
H	-3.45576882291306	-0.69576493193833	6.48854369969624
H	-5.22857360014210	-3.32409825990502	-0.36213273489175
H	-5.26580436556671	-4.43901005551557	-1.72639003317563
H	-5.30954966865539	-5.06209618120073	-0.07976583888202
H	-3.10439028624047	-5.88977597765932	-2.22562232757266
H	-1.79244250437349	-5.84240246846877	-1.04979533686514
H	-3.34721035999035	-6.52407471030749	-0.59985950869962
H	-5.44702847400945	0.17047862031597	-3.22652429347457
H	-5.64611737567823	1.57582171958392	-4.26986961412427
H	-5.66049887756320	-0.04301810187178	-4.96282480384408
H	-3.65029905705740	2.22889492620174	-5.88932804941797
H	-2.27337186924504	1.13409533338618	-5.98824687301629
H	-3.85043206135977	0.61058194512186	-6.55609050514420
C	3.28975362838126	-3.52928087795834	-5.92760893785265
C	1.01506844097372	-2.98684361490254	-6.83397558325617
C	3.66318203444417	-5.37026989898812	3.58744399302434
C	1.46921980738172	-6.42869414910142	2.99642519440775
C	3.10295114495698	5.75682249114111	-3.93738915804035
C	0.72452170058723	6.62921566215903	-3.35605303858535
C	1.06275191119844	3.39825354971389	6.54055780890417
C	3.23858514010078	4.10621517269725	5.51713747042695
H	3.83449962755726	-3.37586201591051	-4.99438759455422
C	4.08760165170660	-2.87139012868012	-7.04502218952925
C	-0.34726895955012	-3.51899513806449	-6.41072059816873
C	0.86837148298275	-1.68810176156155	-7.61497546328567
H	4.14949305695010	-4.40324104745095	3.44658945038553
C	4.54557084552527	-6.43158816882974	2.94410645524980
C	0.06970487899887	-6.10639421035936	3.50183304714634
C	1.40396666371312	-7.21290076547416	1.69302953633952
C	4.07409206483357	6.66317331347403	-3.18457554187238
H	3.53107225259891	4.75131163593821	-3.90608173307797
H	0.00162162624877	6.28524904683684	-2.61549326415542
C	1.17651453148086	8.01544634979342	-2.92063535996467
C	-0.35845292658440	3.80860463178400	6.18185342826952
H	1.45921402846204	4.17434181893810	7.19117099992469
H	3.75263993976903	3.97827348775390	4.56306023961649
C	3.01600985102397	5.59874825241655	5.69877756152637
C	3.17407618962437	-5.03191377724190	-6.12531376624387
H	1.43932722067487	-3.71968386769937	-7.51637047340404
C	3.53192579535405	-5.58045322845735	5.08719959326062
H	1.92682640064757	-7.08222561279587	3.73530514046767
C	3.01895230128555	6.14349840829779	-5.40157671592642
C	-0.03016035667886	6.68007936456612	-4.67757217202516
C	1.06171357678473	2.10388587492485	7.34238203215417
C	4.13282159076706	3.53172631350037	6.60783124919783
H	0.35776994546105	-0.92160383657752	-7.03652576823956
H	0.27269727967168	-1.87864062885033	-8.50641507167366
H	1.82752972981811	-1.29017162670846	-7.93697169422474
H	2.39065008419245	-7.46501855783836	1.31243354349023
H	0.86735389404396	-6.66972943699950	0.91858785376241
H	0.86975363949713	-8.14499079184339	1.87082415652451
H	1.80471512620471	8.49102318528582	-3.67030999761013
H	1.71928115115904	7.99164148035317	-1.97832208395109
H	0.30040139361404	8.64746478799490	-2.78048525075219
H	2.56117746264053	5.83717022056984	6.65884451208081
H	2.37934270684244	5.99772974279050	4.91115369359990
H	3.97256885615201	6.11724113531589	5.65780790078875
H	2.70490483788560	-5.29105693491636	-7.07299853915157
H	2.59569339117847	-5.48688792292733	-5.32322047307695
H	4.16707750535606	-5.47861741303016	-6.12420257760413
H	3.12095788468038	-6.55783789258862	5.33444626316435
H	2.89435911264704	-4.81958427411547	5.53382348985338
H	4.51426173584145	-5.51680386986707	5.55251423753803
H	2.71116055800428	7.17757493925435	-5.53628446663725
H	2.32970930952461	5.50159252392762	-5.94539186786039
H	4.00470187045126	6.03789132173896	-5.85182941453692
H	2.06406387588243	1.79082676602498	7.62386368537515
H	0.59008434197619	1.28991308806341	6.79628249741372
H	0.49320131910704	2.25857581366932	8.25808310432922
H	5.07525843098194	6.51891599239395	-3.58932332047342
H	4.11475305920888	6.42755687116660	-2.12371996361456
H	3.82511252107588	7.71458306915732	-3.29043935432457
H	-0.31569110877193	5.68398807780794	-5.00725853217360
H	0.54134626695984	7.15460964751338	-5.46938901792984
H	-0.94165545638163	7.26050978217082	-4.54011357041978
H	4.18066554167463	-1.79772943745678	-6.89319873204112
H	3.62757083578603	-3.04064860749839	-8.01747480151577
H	5.09239017968968	-3.29046943037841	-7.08003756678464
H	-0.85982142964373	-2.82274188287658	-5.75099631606535
H	-0.25259451300327	-4.47217264492397	-5.89502691169437
H	-0.97207641639104	-3.67014701099773	-7.29054736189157
H	4.65485198624526	-6.26843566404803	1.87363706503167
H	4.14317640985831	-7.43168392296296	3.09833111847749
H	5.54021721675290	-6.40593242667948	3.38729772215112
H	-0.47594695550578	-5.48485392474005	2.79561376057193
H	0.10778131432004	-5.58546122089614	4.45618629068739
H	-0.49240569695600	-7.02934581563485	3.64180934289279
H	-0.84459593866035	3.05909217098743	5.56176432039065
H	-0.37032404468998	4.75475881150343	5.64552893937510
H	-0.94885387330023	3.92693951055007	7.09002185964737
H	4.30286615839188	2.46605893360517	6.46731060120288
H	3.70187997570566	3.68139975691675	7.59667711514174
H	5.10214827048278	4.02819421484006	6.59329368252298
H	-3.20877115471973	2.03473671632397	-3.66703196920685
H	-3.30511441058837	3.94091435809984	0.20501968725921
H	-3.21225263723726	2.25689144002747	0.64157677532332
C	-3.61548986646137	3.54167890774708	2.29454371929024
C	-3.31842994351099	-0.26573625776909	4.32681992196214
H	-3.02320417648161	-0.87251845446907	2.30129185312716
H	-2.93113758869942	-2.18573730466119	3.44115358813273
C	-3.25928680353123	-4.41734797968749	-0.16623729693765
H	-3.04261935181711	-2.42031696301670	-0.90240407843195
H	-3.02748404019835	-3.63102171352526	-2.15077920289671
H	-1.04514400310336	1.45393112457609	-4.53469909237513
C	1.78944623013056	2.71419029369694	4.88768247388131
C	2.48972822620617	-4.47092329363177	2.12880856883154
H	1.65486776136214	3.26284017741994	-4.30469144251242
H	1.26753723544781	4.53938704929495	3.36787912569146
H	1.03712550051017	-0.87389417197875	4.59569099659719
H	1.97950419184745	-3.07179855553528	4.03662745284841
H	1.09348515856169	-4.53059126447061	-1.27495306773925
H	1.58363331497714	-4.27750032040863	-3.65203366693416
O	0.97962381029353	4.82457891212452	0.08527016663171
C	2.02274346441751	-2.42216078544190	-5.03060341229272
H	0.84843993092806	1.05904473784787	-4.89181557887130
C	2.11641329930854	4.66299734099558	-2.39611250668604
H	0.72636224542553	4.66782202170350	1.01532703478414
N	1.57521117114605	-3.01311451487586	-6.27835167216608
N	1.86774785311269	6.07711187907626	-2.68685043921445
N	1.28676962665455	2.91303691503515	6.24764130541227
N	2.29318924421866	-5.89566893594744	2.40656694452512
H	2.60397254309557	1.99875593685520	4.93929252111899
H	2.24930334048830	3.64184540799373	4.50360154132540
H	3.17326733647990	-4.39295403394121	1.28932559216343
H	3.01733610457930	-3.98530633474197	2.96800675670339
H	2.64377730418426	-1.55045175495244	-5.23824514567673
H	2.69440275922617	-3.10528267469742	-4.48027444795722
H	2.79868788327459	4.61826377596432	-1.55338790712095
H	2.66671089602092	4.19062846460652	-3.22825272439112
C	-5.11322518139691	3.30332198933862	2.15714648981320
C	-3.33458721167443	4.94319377737578	2.81712780449786
C	-4.83058139694929	-0.37571594941831	4.18714978421300
C	-2.87921116098926	-0.58988562725216	5.74780162606494
C	-4.77893952273334	-4.32119960998011	-0.19523067933217
C	-2.79852915946826	-5.84294676695572	-0.43644276041131
C	-4.95557593042033	0.03176953737321	-4.35459389203760
C	-3.09387670929348	0.48394682135687	-5.97256130770700
H	-3.04994978143063	-0.91478648396405	-4.36720869828469
H	-3.23359594072841	2.82902297759624	3.02898200098582
H	-3.03918476068090	0.77012981271656	4.12238879087005
H	-2.92842321836043	-4.14617742653814	0.83908304963057
H	-5.55738174817251	4.00526255799923	1.44892725955650
H	-5.32160895025420	2.29470000667230	1.79971822403598
H	-5.62178309554523	3.42959089262048	3.11285474594766
H	-2.28055097296443	5.09436927999181	3.04133285238226
H	-3.63270725250617	5.69729103855841	2.08600641208297
H	-3.89130430967110	5.13233643903733	3.73486723150637
H	-5.15590230170118	-0.12732822068887	3.17663021396067
H	-5.16871692287029	-1.39079213888933	4.40407664423012
H	-5.34009757530727	0.29740181381321	4.87654731353985
H	-3.06137666841532	-1.64067812605971	5.98225127425057
H	-1.82089863848307	-0.39072603181278	5.90467219136419
H	-3.43224134411160	0.01133450872969	6.46931015243074
H	-5.11691982004835	-3.30838760696851	0.02663169382761
H	-5.16810244316176	-4.59279144379961	-1.17829134473342
H	-5.23020284819609	-4.99148847437271	0.53621110354569
H	-3.02359333958803	-6.13791001577794	-1.46347481372726
H	-1.72773063443454	-5.96392223320199	-0.28132178361737
H	-3.30309513178354	-6.54425311987173	0.22796535321547
H	-5.21559890913961	-0.28738412127727	-3.34491562277742
H	-5.40674980716064	1.01196884339400	-4.51963455761700
H	-5.41532516181833	-0.66586857818655	-5.05457416110157
H	-3.41667322792937	1.50588792497477	-6.18091231670267
H	-2.02405540577814	0.42620486033576	-6.16224418312139
H	-3.58632955430243	-0.17269188688975	-6.68957173646227
C	0.19769800392825	-3.41194631803493	-6.50680707230975
C	2.50663344078691	-2.99821681699352	-7.39256908147293
C	1.56170757400124	-6.12552243059704	3.67418217832173
C	3.55552535608752	-6.64676477338864	2.23323349663493
C	1.15627955108534	6.27026145140430	-3.97198687360036
C	3.09605929256835	6.87943126957129	-2.49614307837533
C	2.39104899831581	2.94771900278492	7.22787375722394
C	0.28077698149650	3.99905316377389	6.32779360424597
C	0.07748142224356	-4.88924849277999	-6.87838819042020
C	-0.53683932505539	-2.52879287230579	-7.51477908837178
C	2.82559138491711	-1.59642110670763	-7.91825259175209
C	3.78925477414784	-3.76179405148365	-7.07254619765805
C	0.05291796023821	-6.04872209852619	3.44816834689996
H	1.84409059855371	-5.34140487179525	4.39612389285378
C	3.92382704726940	-6.78625485532074	0.76293310680929
C	4.73745710144822	-6.11896880515837	3.04785304124948
H	1.49144417726922	5.49954913307137	-4.68611900152038
C	-0.35170398415127	6.12094717061665	-3.78240847396027
C	3.43649947509669	7.03780816165662	-1.02120389958284
C	4.31038364042162	6.39791196100085	-3.29138715653631
C	2.97094718327818	1.55849853337860	7.45507322621233
H	1.93781558097487	3.23307873891353	8.17171643892502
C	-1.11953228167601	3.45447983049172	6.05152937466706
C	0.23713078550991	4.73762747794801	7.65886012926051
C	1.85829044704425	-7.45793567374155	4.34938610967974
H	3.34579118883961	-7.65461485331550	2.57636043194651
C	1.40458259647816	7.61484421644901	-4.64241874942491
H	2.85024373891213	7.87699011397578	-2.84523133484634
C	3.49628544864755	3.95991540956486	6.92187704770715
H	0.51339472363094	4.75489642165175	5.56197621111463
H	4.36151083823415	-3.26684340404419	-6.28804815789879
H	4.43037086734962	-3.82666841967999	-7.95148194882520
H	3.55996087214442	-4.77264002017995	-6.73778956004419
H	4.46648295951157	-5.95317485423337	4.08964618347870
H	5.11907601492914	-5.17994547938287	2.64876029342598
H	5.55638720862753	-6.83744114903029	3.02753729285312
H	4.06289081024750	6.22171956364434	-4.33724575790045
H	4.72099884586383	5.47410216122659	-2.88562953170072
H	5.10059390763333	7.14736778532096	-3.25802234398334
H	1.16394553690888	5.25026377152255	7.90123951277167
H	-0.01561935553293	4.06738260415796	8.47996762744885
H	-0.54692093402982	5.49069483219562	7.60423296013263
H	2.89109065005572	-7.56340211832342	4.66963474948755
H	1.60640368819953	-8.29734319752424	3.70169922355568
H	1.23369472354534	-7.53570080819959	5.23741344109583
H	2.43834959365537	7.76966590056135	-4.93820930173662
H	1.09689856478359	8.44154792151544	-4.00245008405657
H	0.79862961436716	7.66210214209236	-5.54542452794248
H	3.08862110968003	4.94209291148829	6.68713763434326
H	4.11386038006056	3.64524262380447	6.08152326128977
H	4.15278248609171	4.06922944053180	7.78462709274139
H	0.54944481047365	-5.51281520714349	-6.12014127481384
H	0.55167888307777	-5.10468134692220	-7.83619298306626
H	-0.96945098463381	-5.18367259451091	-6.95956920169577
H	-0.26306871219334	-6.87624358700185	2.81293325605259
H	-0.25491444901639	-5.12742679739541	2.96948793468910
H	-0.47342622925134	-6.12604450613964	4.39970696933703
H	3.09009322395853	-7.19574932478235	0.19712280599724
H	4.77463879884500	-7.45984702906131	0.66278693020290
H	4.20623866133524	-5.83855414111828	0.30765157957665
H	-0.72109570677056	6.92579613038004	-3.14692550671834
H	-0.62758500635551	5.18111030365093	-3.32037945296755
H	-0.85896335273050	6.18371372436621	-4.74524960470088
H	3.75322317645197	6.10430261231466	-0.55940526678168
H	2.57780716422294	7.41195415172495	-0.46838195276647
H	4.25575402530278	7.74798549298857	-0.91130201318962
H	3.49243375254829	1.17379187961829	6.58010536994198
H	2.18443866754292	0.85358191425396	7.71491742815291
H	3.69103440245097	1.59127883770964	8.27251438811323
H	-1.40448803660280	2.76255466203917	6.84399083720319
H	-1.18251822802035	2.92507042139709	5.10893652400430
H	-1.84451341972941	4.26842612947619	6.02829599493749
H	2.01382751728116	-3.53564993567108	-8.20275368332352
H	3.36953474915817	-1.00565340718978	-7.18187958564200
H	1.91350843133773	-1.06125246132257	-8.17033824887433
H	3.44825298514215	-1.65641291791657	-8.81124413254675
H	-0.31483816202442	-3.28656080767152	-5.55617948551814
H	-0.11362493104317	-2.62588409487847	-8.51460535554568
H	-0.47762156168140	-1.48332524987703	-7.22022776288677
H	-1.58937701134575	-2.80761006975363	-7.57556773957507
H	-3.09522280271708	2.26331451817805	-3.42448356347815
H	-3.04110304994035	4.21643361428893	0.74943471131358
H	-3.01863488242481	2.50121526257484	1.04487922554442
C	-3.22785720238563	3.67690995317302	2.81857146863340
C	-3.11167817718249	-0.06656312672664	4.65779190671635
H	-2.95194994434933	-0.72616003728239	2.62974064128880
H	-2.88787695659536	-2.01843573080354	3.79266920042719
C	-3.46056726131326	-4.18787034878859	-0.18829142400394
H	-3.06624262803693	-2.15491741069514	-0.69421070546931
H	-3.08855925975791	-3.22015850424018	-2.07186439744970
H	-0.93751796410417	1.64631787569254	-4.23910645412556
C	2.61179178961593	2.30621525355604	4.56131651435100
C	2.06082173461706	-4.90581877619943	2.45204058394118
H	1.21855242358723	4.05554664825958	-4.17650959504750
H	1.06521270135295	4.50075341415192	1.96620509520776
H	1.22023800074673	-1.10266154131733	4.61828787203117
H	1.83359384722850	-3.37612376550004	4.25148552918154
H	1.44832882430659	-5.59135985879970	0.39897612952769
H	0.97424682782361	-4.23488909565543	-1.72522973037087
O	0.79577159253059	5.26058709343386	0.32120801087220
C	2.41422793351462	-2.02929450551360	-4.37405653654213
H	1.00883372313639	1.37174027101021	-4.42330327866994
C	1.95752892877253	5.08718682049112	-2.39053231277420
H	1.56319261293496	5.72126701705410	-0.03928812411469
N	2.14369852794720	-2.28913668840729	-5.79421393306724
N	1.71328899706088	5.53047541013141	-3.77391629922040
N	2.36224012158058	2.55968377619374	5.98621678875219
N	2.24444070785401	-6.09409061736058	1.57920688948239
H	3.29978456257137	1.46404058947023	4.50601525988489
H	3.10348408697163	3.14668836692631	4.06377741039893
H	2.99402535090136	-4.34163142751995	2.46523135265259
H	1.90538488086665	-5.23749243563571	3.48263600723941
H	3.09370517913461	-1.17939413561546	-4.33297991923321
H	2.92329647860334	-2.86360101509935	-3.88375835802077
H	2.16977189803809	5.93096181704131	-1.73131114027851
H	2.87354699944901	4.49494742896722	-2.41439809839392
C	-4.74557990173348	3.56459528889835	2.76914527318355
C	-2.79778689148981	5.00394857699409	3.42795664050121
C	-4.63294539303670	-0.10114414084235	4.60394815038623
C	-2.60859508500158	-0.38260282376453	6.05909636255908
C	-4.96228000729395	-3.93294448519336	-0.20665671052426
C	-3.14669505289029	-5.61096312840806	-0.62750014750597
C	-4.88524414080221	0.34196230939645	-4.10137554341463
C	-2.92906222651970	0.56672099647594	-5.65704501505592
H	-3.04651781256177	-0.72115180227210	-3.96962094523230
H	-2.85683390094785	2.88022585192794	3.46617904485959
H	-2.79343454148526	0.94892492671056	4.41468474152069
H	-3.11679434634989	-4.07067666677719	0.84306023382489
H	-5.17487490104576	4.35207416017044	2.14676317246541
H	-5.05712841010570	2.60612157381888	2.35352691747856
H	-5.18128235379770	3.65374170180045	3.76419184897959
H	-1.72483644641311	5.04189453816979	3.60855462225356
H	-3.05709624070881	5.83706508147209	2.77153734787864
H	-3.29325343932926	5.16894747894476	4.38469479590588
H	-4.99997801854502	0.14521230657731	3.60735884499464
H	-5.00942572663472	-1.09332375255921	4.86022426898657
H	-5.06887474891304	0.61034169061718	5.30512226072013
H	-2.81789127485165	-1.42080995527261	6.32529782883048
H	-1.53594969443629	-0.22245032953930	6.15498268190659
H	-3.09804220070961	0.25334226180040	6.79665179460340
H	-5.19680594029228	-2.92659809927355	0.14177270990709
H	-5.36343074030714	-4.03635640734888	-1.21661948442104
H	-5.49104322031022	-4.63986438088712	0.43285861447195
H	-3.40818049654527	-5.75845314951253	-1.67720507509535
H	-2.09221411152838	-5.85381890551073	-0.50986335275344
H	-3.71498089791971	-6.33229906973571	-0.04004103751834
H	-5.20799955093993	0.12091410250836	-3.08334347733691
H	-5.26432563356885	1.33217177814230	-4.36147362941996
H	-5.36019241813551	-0.37931687781414	-4.76645174658164
H	-3.14509293129263	1.59914479434290	-5.94003812105465
H	-1.86261232951763	0.39828133519075	-5.79618796195925
H	-3.45462493435691	-0.08498116208243	-6.35501187542366
C	1.34008115588552	-3.50402244158437	-5.99425757870224
C	3.31982978459374	-1.98988231748438	-6.62195412692433
C	1.58677573511354	-7.28971184048412	2.18286274278160
C	3.68023515435118	-6.22113022923092	1.16767192726229
C	2.97293101512614	5.81616047001404	-4.50789603889853
C	0.64754727125600	6.56748507654914	-3.93919452430169
C	3.55882574680744	2.27988993340239	6.79195371948209
C	1.54170995388375	3.76080412893181	6.20271338150102
H	0.71361622121082	-3.53925237697906	-5.10495444797329
C	2.10387235823944	-4.82753196433201	-6.03220236998818
H	3.54912287759843	-0.94928609450866	-6.37120002542927
C	4.59717826713561	-2.78494494171270	-6.31758672358376
C	0.11345893760841	-7.01197315879617	2.45703888625998
H	2.07364728875702	-7.49545449790896	3.14356037624466
H	3.98916703925289	-5.18677980698753	1.02320361715702
C	4.57451827551208	-6.81719757018961	2.24286410819969
C	3.67412709261708	7.10050295148103	-4.08662517326056
H	3.62749496312519	4.98074870513196	-4.25457053967527
C	-0.68656235151451	5.94183494006340	-4.32075841121407
C	0.48484244502881	7.51146183230308	-2.75615539626278
H	3.79963796831968	1.24254517422218	6.53824858951180
C	3.30188772708999	2.30024630470726	8.28873779430496
H	0.89848440879250	3.78658861638825	5.32519495890855
C	0.58451749335042	3.64094106111879	7.38434319297995
C	0.36107424234764	-3.40235634116838	-7.15963927336113
C	3.03477854279876	-2.01183954184665	-8.11370943011930
C	1.69188217783143	-8.52919698772511	1.31198845008403
C	3.87509495043600	-6.88083682727507	-0.19063685691756
C	2.75003531613299	5.75994455774837	-6.00995966363489
H	0.96348300932608	7.17375034708439	-4.78448083325465
C	4.81714836503638	3.09440298511476	6.46189464786132
C	2.28323954907696	5.09694501805957	6.22967857790640
H	4.80398325304777	-2.81973010860916	-5.24985173576052
H	5.44897529679946	-2.30471157835810	-6.80079542880525
H	4.54336751511971	-3.80673492119609	-6.68425887246218
H	4.37581315320947	-7.87519864566599	2.39922206360911
H	4.45116569205472	-6.30379897754959	3.19542986660061
H	1.42251248413277	7.98114712786222	-2.46900158370386
H	-0.20961898480802	8.30332424642904	-3.03107783841211
H	1.07751917633531	3.75572011570161	8.34542193891512
H	0.08235386201678	2.67536687128345	7.37055290655176
H	-0.17541960071891	4.42018301879659	7.31427213541002
H	0.83964723058246	-3.51078987597112	-8.12871697980149
H	-0.15628911715080	-2.44494422285874	-7.13909117006163
H	-0.38514188704849	-4.19348820192592	-7.07621607107695
H	1.33111038672835	-8.33043604552906	0.30446234912503
H	1.06866666023813	-9.31113237857867	1.74118260183848
H	2.15847571418095	6.59840254804836	-6.37378723265462
H	2.24813352879100	4.83764188923295	-6.29636145692443
H	5.00367233674079	3.12995067416858	5.39046743998381
H	5.68528484917009	2.62881368680559	6.93000229257212
H	2.66354534086485	-4.94574925578180	-6.95835678630558
H	1.40489330712763	-5.66183448095877	-5.96295350590289
H	2.79874412021722	-4.90803705410463	-5.19848649766946
H	-0.43055180683680	-6.84106736857616	1.52975953726123
H	-0.05068039238225	-6.15757852241924	3.10602729990264
H	4.64239290165116	7.17112320691995	-4.57980430381477
H	3.84723571929490	7.13179194967627	-3.01246948877037
H	3.09858834695328	7.98154832683967	-4.36647167692869
H	-1.10235923621958	5.35778998000308	-3.50283381950606
H	-0.57927343826156	5.29116135529123	-5.18557926800438
H	-1.40122184920847	6.72493143238339	-4.57159706992708
H	2.42362146156508	1.71311350688084	8.54770040120980
H	3.16621505819003	3.31091420687926	8.66731395959965
H	4.16123185168763	1.87435373991915	8.80564966340668
H	3.87616710446974	-1.56945523995288	-8.64621837567223
H	2.14134786032002	-1.43974304219620	-8.35384307700570
H	2.90965991508926	-3.02395123094497	-8.49214562755113
H	3.15728787775641	-6.50679856146187	-0.91737380576018
H	3.80404782442999	-7.96215897162033	-0.16229268071818
H	4.87147187180639	-6.63074311276933	-0.55257998422041
H	-0.32061938431672	-7.88156071249654	2.94588319137761
H	5.61743465965386	-6.72007236638254	1.94619395058786
H	2.69991159478206	-8.92271846174184	1.24856814316137
H	4.75445963800632	4.11616114917809	6.82729672400083
H	2.96109215780277	5.19205802645078	5.38357683156052
H	2.85793467915056	5.22249320641762	7.14554094701234
H	1.56858094550163	5.91899405787797	6.17608250347380
H	0.07035325820795	7.00560324120054	-1.88607150988350
H	3.71032600097531	5.79695073614389	-6.52148178635372

5

C	-0.80284184760027	-2.10039443046143	-2.50408223151718
C	0.23619078477152	-2.36970666406940	-3.39707713944398
C	0.74489172824455	-1.38316476213529	-4.24946076931928
C	0.18433320160122	-0.10210168108115	-4.21151685505663
C	-0.88286414326287	0.19152008520148	-3.35221310632705
C	-1.32107790147094	-0.81460567924433	-2.49995617062730
O	0.77553380077854	-3.62771178492041	-3.41792258906802
O	0.72299138846939	0.82828519399679	-5.05645445458818
H	-2.12457703971903	-0.58612070231136	-1.81481667681511
C	-1.55215879084863	1.55321153342451	-3.34949965687617
C	-0.96758566959248	2.45352491437343	-2.28204412341378
C	0.11600044869794	3.27905835364142	-2.58896805182870
C	0.70897996016509	4.09986952108414	-1.62279472547948
C	0.19545187579232	4.09402885454667	-0.32185335484888
C	-0.91531765683114	3.30479392150032	0.01160945225929
C	-1.44438201896067	2.48645255111044	-0.97978562319956
O	0.61244726253787	3.26958243830603	-3.86383764894649
H	-2.27740002446951	1.84912121102513	-0.72404284789324
C	-1.50060479626238	3.33171879649888	1.41066764483880
C	-0.83687719556127	2.30176034388529	2.29911324679066
C	0.21359435466866	2.66463706303885	3.14157439078362
C	0.85541633836706	1.72638232406496	3.95849980531493
C	0.41476689562538	0.39964824154183	3.93889612957390
C	-0.66632480662085	0.01183667983491	3.13501917487131
C	-1.23498888708880	0.97410246126416	2.31191923552833
O	0.63187565138442	3.96818972970650	3.14729687173556
O	1.07972694784466	-0.48041999125835	4.74753660218907
H	-2.05454228066251	0.68080600443487	1.67118315794257
C	-1.21322986653711	-1.40157371564397	3.16870856939734
C	-0.61607526074570	-2.25013113851095	2.06763591912429
C	0.54043273808776	-2.99388375275313	2.31351297673433
C	1.13186562239252	-3.78064158689258	1.31858669720228
C	0.54570250200689	-3.81847840152683	0.04894332725572
C	-0.63043366306455	-3.10649038432372	-0.22241049539285
C	-1.15863013966522	-2.32055809934686	0.79403030348878
O	1.10693873992372	-2.93888778521534	3.55770499619965
O	1.17426784342963	-4.59114116026081	-0.88726604974133
H	-2.05367137893748	-1.75385880468444	0.58328612381819
C	-1.30609353941917	-3.18634213446567	-1.57644655207298
C	-3.07920881620092	1.46311215167841	-3.28207954530420
C	-3.02819624437893	3.22857546339123	1.42412265788427
H	-1.26622457947450	4.30855164531028	1.82665587956499
C	-2.74341415005817	-1.44996084494695	3.19082668791912
H	-0.88904516357074	-1.84878670613157	4.10489955182962
C	-2.83474345992563	-3.22134549063802	-1.49524021495211
H	-1.01285980451840	-4.13193039362580	-2.02534918210742
C	-3.72912880254176	0.65509911922095	-4.40254767481842
H	-3.39540095298993	1.03954408452921	-2.32901580188685
H	-3.47454853055664	2.48218355406970	-3.30179652293190
H	-3.42513987887126	4.01046684156930	0.77139323577710
H	-3.35173974193088	2.28367439273214	0.98823155836047
C	-3.66777967015748	3.35559262306985	2.80497787946380
C	-3.40169855814768	-0.69240044733426	4.34118750216296
H	-3.15429207017677	-1.07241814485266	2.25471048732128
H	-3.03962477736971	-2.50131029419159	3.24055192933608
C	-3.41937466709860	-4.34653104305908	-0.64504935832616
H	-3.22283370143761	-2.27298214758609	-1.12372376860919
H	-3.21351922629779	-3.31409531303819	-2.51666457899452
H	-1.32547922264866	2.02693164956566	-4.30156861887424
C	2.04178344262372	2.13910347439829	4.78353745398192
C	2.41402108432519	-4.51390122541506	1.59874665408896
H	1.23539409145476	4.09735136177284	-3.92191562691463
H	1.22464777355671	4.06002453409087	3.99009646206070
H	1.03658494079212	-1.38719476617304	4.37165363267553
H	1.77554509635333	-3.73015256444380	3.58883226232674
H	0.96780393975190	-4.28410422454125	-1.79691474901076
H	1.33967072464635	-3.66916168815427	-4.28412296483702
O	0.82954415146705	4.90257386358633	0.57937055246040
C	1.92512725066438	-1.68768247449102	-5.12860834009101
H	0.62670806180884	1.72910686966992	-4.67476780604195
C	1.92940498501083	4.90909198854734	-1.96432265492083
H	0.68849975800564	4.59808527519975	1.50176875671189
N	1.87092393325870	-3.05827076297605	-5.64748537547375
N	1.88223218594384	5.40664753963552	-3.34319684665847
N	1.87493431132780	3.49275300149596	5.32226828297655
N	2.45981573489923	-5.01143931853254	2.97743510464673
H	2.22893452102807	1.42246618947332	5.58537197599941
H	2.93546270235920	2.15353535551323	4.15447922057561
H	2.55992791469253	-5.33050383303675	0.88910324056841
H	3.25958056863518	-3.83262226766141	1.47536578932455
H	2.00222408105186	-0.96828599109517	-5.94617806562149
H	2.84494426965630	-1.60302127265081	-4.54426769327500
H	2.06097916857205	5.73420493196269	-1.26190986347294
H	2.81829816517011	4.27878667974542	-1.88099671772737
C	-5.17250107015895	3.14808575907346	2.69981856490488
C	-3.34864099964071	4.68101519371602	3.48216792446795
C	-4.91722685686551	-0.76915852876652	4.21392339934714
C	-2.94508465567040	-1.18851506604146	5.70606688518615
C	-4.93833018632936	-4.23915496270697	-0.61761986648298
C	-2.97902124023604	-5.72442153366695	-1.11937665057247
C	-5.23401421574910	0.57707367552876	-4.18203439954168
C	-3.41155262340139	1.21053581783117	-5.78377504747225
H	-3.33226289747680	-0.36204076194825	-4.35198134389853
H	-3.26354519925110	2.55551973917546	3.42966674678647
H	-3.11232477389823	0.35777692038837	4.25599116927446
H	-3.05759071499604	-4.21356077914155	0.37751720900270
H	-5.63107997483703	3.92719725580567	2.08782101465898
H	-5.40796464321014	2.18634181379375	2.24370496087524
H	-5.64611682441582	3.17646264433344	3.68127351996806
H	-2.28394999220092	4.79646157777870	3.67650971788789
H	-3.66810660415590	5.52107482307851	2.86213651528506
H	-3.86549601256652	4.76021310635316	4.43864317501072
H	-5.25338491729353	-0.38991820600609	3.24869908412936
H	-5.26334457360749	-1.80042478857799	4.30539158174740
H	-5.40905687792552	-0.18421993628123	4.99121401970173
H	-3.14638058468776	-2.25575697243867	5.81904075536502
H	-1.87910395099354	-1.03191059778771	5.86108149300919
H	-3.47170700193426	-0.66552769510354	6.50439657105998
H	-5.25927854344525	-3.26555918661272	-0.24574456442664
H	-5.35652201900389	-4.36694845261073	-1.61790778833463
H	-5.37720618411118	-5.00327813247457	0.02411663449058
H	-3.25152500948743	-5.87993942593895	-2.16522898493401
H	-1.90265378118414	-5.86120452752104	-1.03128046873722
H	-3.45744631992996	-6.50821848791220	-0.53207362450170
H	-5.47008058340134	0.14716272572008	-3.20805541946957
H	-5.68789831921895	1.56916756883175	-4.22471490889937
H	-5.71347415116636	-0.03787415010018	-4.94383026683794
H	-3.72533212208139	2.25338309725009	-5.86492346474459
H	-2.34745467506058	1.16486516345326	-6.00931100050886
H	-3.93318597817389	0.64696805717302	-6.55747911630690
C	3.12108334933208	-3.52798133975939	-6.25161840364975
C	0.85559530058533	-3.27294719152654	-6.68670429039208
C	3.78774389350916	-5.44016995053167	3.42662077289554
C	1.59145979756312	-6.16623468323454	3.23793978276770
C	3.16455697350509	5.89891566521076	-3.85585946643950
C	0.94715654981855	6.51795112533693	-3.55810625458196
C	0.89259621831111	3.59664527385065	6.40897795303683
C	3.09891432399727	4.08074079386996	5.87488949927563
H	3.87701663826438	-3.67885105188403	-5.48371211638282
H	3.50006803184843	-2.77833565151832	-6.95822405374532
H	-0.14263525285274	-3.21040802884974	-6.26022702310090
H	0.94315032896280	-2.49648174306921	-7.45725068772485
H	4.43850509634203	-4.57681544558405	3.54845573799483
H	4.23997204677156	-6.10232980605643	2.67726062050381
H	0.54515915605513	-5.87531135724105	3.18244252645116
H	1.76828486983655	-6.94298064650976	2.48314885420329
H	3.62096578614691	6.58113742355953	-3.12742755735833
H	3.84894556240918	5.06782501640463	-4.01265749882062
H	-0.07977320556444	6.17564470028225	-3.45454831117500
H	1.12097166995291	7.30090631680019	-2.80911828336921
H	-0.11301277318638	3.43071280216226	6.03012895802577
C	1.12193345450730	4.99795595574866	6.95991754910877
H	3.79586478698867	4.32025647688792	5.07435944225708
C	2.60554039446140	5.30040969404093	6.65060206114744
C	2.71743136214909	-4.80665436480226	-6.98114214607911
C	1.20318372056605	-4.64693382646953	-7.24672479419674
C	3.51082577219284	-6.19631967357813	4.72451913434077

C	-0.63933564038250	-2.34458613591902	-2.28221863710234
C	0.37225183174017	-2.72006869424022	-3.16758914381332
C	0.87342766903507	-1.85526338261891	-4.13987661225223
C	0.33775256469696	-0.56018391249217	-4.21142615646755
C	-0.70707942505352	-0.15854132525911	-3.37166782536459
C	-1.14569967537799	-1.06141304340647	-2.41271550686761
O	0.85570672601665	-4.00918877857232	-3.03559238223054
O	0.87564530567974	0.26609882665777	-5.15218823852601
H	-1.94132955182691	-0.75419476805895	-1.75020571576069
C	-1.37302435454154	1.19580215039362	-3.52610361617209
C	-0.78765810061096	2.22191415369176	-2.57683365219763
C	0.25589577940518	3.05417638105598	-2.98386561877940
C	0.83663338328389	3.99233740695111	-2.13124445261147
C	0.33733024082462	4.10068435601578	-0.82484578124891
C	-0.73513063197154	3.31210994378546	-0.38773452797359
C	-1.24800536213649	2.37778962826360	-1.27814105487115
O	0.69131393988947	2.90129460522840	-4.28518280683162
H	-2.05910016834881	1.74773835614518	-0.94719917068358
C	-1.31900615773212	3.48348865456672	1.00271589392923
C	-0.65560272350912	2.55957297810777	2.00413435473444
C	0.38528716358966	3.00503839920985	2.81827186657623
C	1.01454608836815	2.17366545207968	3.74467526186739
C	0.56479646567195	0.84997556525870	3.85921551878309
C	-0.50525199890181	0.37651230835970	3.09072648252833
C	-1.06367989220163	1.24513543586145	2.16358053345794
O	0.76683765340648	4.32245546535138	2.65746045580568
O	1.20956821864639	0.06708509235493	4.77136469903583
H	-1.88458374773222	0.89059665637797	1.55723456681369
C	-1.06265572495313	-1.02101789358876	3.28281378366860
C	-0.46551791565027	-2.00096404510902	2.29409646791019
C	0.65204533192135	-2.76534870203263	2.63492411114146
C	1.23763517846240	-3.66562299388555	1.74583834585482
C	0.66751276776264	-3.80518491490229	0.47151035447938
C	-0.47823466208156	-3.09002480846024	0.10625270841985
C	-0.99389250234087	-2.18790615838284	1.02703045906976
O	1.15268400297746	-2.58547132758718	3.90922125507014
O	1.27884131638095	-4.68835654144517	-0.36772524144511
H	-1.86925626781408	-1.62088625033201	0.74743376216272
C	-1.15229951121394	-3.31096334978814	-1.23346377526363
C	-2.90107421127819	1.11708366908490	-3.43827308423650
C	-2.84713769248976	3.37617255567989	1.01867953316155
H	-1.10037756307566	4.50282938426081	1.30887886017507
C	-2.59397276415341	-1.05585944530714	3.29273665640021
H	-0.76458537260821	-1.35658246509207	4.27215145104503
C	-2.68208552992734	-3.31666962228275	-1.14991807945938
H	-0.88217328341625	-4.30867504720210	-1.56810056153223
C	-3.55973104287186	0.15738356254373	-4.42658159652585
H	-3.21491125038651	0.84134937614029	-2.43194775611714
H	-3.29148328131939	2.12422092608110	-3.60555729913818
H	-3.23710330449028	4.08383705178946	0.28284060915261
H	-3.16715763323010	2.39042104623943	0.68212313664069
C	-3.50062367273689	3.65009219682593	2.37108909253980
C	-3.25492656117584	-0.16564154402627	4.34198395324670
H	-2.99148390136640	-0.78571074949728	2.31493970024612
H	-2.89851329345565	-2.09225312371172	3.46081203105084
C	-3.28084098891249	-4.32321183201714	-0.17055043417205
H	-3.05873473338569	-2.32573152143410	-0.89761420378096
H	-3.06064830925325	-3.52927165396596	-2.15316218995220
H	-1.16067454030594	1.54460028048766	-4.53278792268881
C	2.13848627797281	2.64620472050477	4.61705739559465
C	2.43837336888362	-4.48909635383264	2.10448797298972
H	1.47359899472923	3.44876525047381	-4.43824112338871
H	1.55236771593398	4.50647086467936	3.19044973455979
H	1.12880925122852	-0.87332899900339	4.51884357401915
H	1.97252465895325	-3.08708022064434	4.01571469303232
H	1.06291449870634	-4.48197526668117	-1.29861578380868
H	1.64968615510574	-4.11583423734670	-3.57738316419025
O	0.94165319569729	5.02550768510114	-0.02659070075274
C	1.95470739783911	-2.25918687174071	-5.09671213319840
H	0.74769861616391	1.19842634701435	-4.88894444897200
C	1.96849335949208	4.87745789026428	-2.56073372792268
H	0.81007598237333	4.80609927591216	0.91663327229503
N	1.45614409003152	-2.56277052076232	-6.43950612223192
N	1.53974151748703	6.20561728438981	-2.96238039907403
N	1.70516000744873	3.02982007990125	5.94927606952353
N	2.10287065607562	-5.84693117510217	2.49488125112962
H	2.86092829427155	1.84001048172602	4.72346150493767
H	2.69618444580033	3.47580269940483	4.13782030610904
H	3.09443260869127	-4.54654827139579	1.23867877806560
H	3.04048732811603	-3.99702419398667	2.89428258966295
H	2.64993052435567	-1.42897073111143	-5.19467398398929
H	2.55842076935790	-3.08706806152592	-4.68061676569398
H	2.65801187155114	4.99082456929532	-1.72732642312222
H	2.56194562812425	4.40255223136976	-3.36761191949388
C	-5.00651167345592	3.44858095818528	2.26665494866341
C	-3.17385304852203	5.03446151645646	2.91273091366236
C	-4.76973835295760	-0.26205242726692	4.22225727931779
C	-2.79995560830081	-0.48954596571174	5.75818358637892
C	-4.80006385009577	-4.21672659047533	-0.18862225913374
C	-2.83213691735314	-5.75098633689349	-0.44948616834804
C	-5.06264773529906	0.11623657473180	-4.18301483219740
C	-3.25194144570392	0.50872970769855	-5.87533983691974
H	-3.16520134650961	-0.84329041398177	-4.23350674656298
H	-3.11677044761097	2.91450610477473	3.08175630155024
H	-2.96867796161485	0.86692709596659	4.13027690267482
H	-2.94052952468994	-4.05751094777732	0.83317444872826
H	-5.45142054603916	4.17662649918026	1.58582177342403
H	-5.24666965582933	2.45353012064786	1.89156894138859
H	-5.48881668606911	3.56421953716606	3.23721367789851
H	-2.11165984261155	5.15491500181805	3.11799598851795
H	-3.46601879730791	5.80887246505818	2.20080470825902
H	-3.70789935477195	5.22168221204534	3.84427841643468
H	-5.10584458709613	-0.01294783539954	3.21549577493285
H	-5.11403686050243	-1.27357893377624	4.44587291760657
H	-5.26420151247124	0.41713372417517	4.91658631222799
H	-2.98366505861877	-1.53914174980594	5.99663369897647
H	-1.73860027400570	-0.29495708852505	5.90175605475191
H	-3.34185709388904	0.11485845133045	6.48546392798862
H	-5.12979540964251	-3.20171615133685	0.03555673627891
H	-5.19736039338106	-4.48507939749479	-1.16930576917898
H	-5.25097752562356	-4.88446707500723	0.54534929972950
H	-3.07000269035825	-6.04162898645071	-1.47479032592618
H	-1.76053591228323	-5.87921907412084	-0.30617189279384
H	-3.33461155503850	-6.45098435173928	0.21792106167102
H	-5.28857882842546	-0.17524759634858	-3.15677177821951
H	-5.51400784617730	1.09464280289921	-4.35782357881097
H	-5.55038338922499	-0.59665468405158	-4.84778513586065
H	-3.56492410101727	1.52996146682830	-6.10151640926375
H	-2.19017374263201	0.42689198093266	-6.10163755113524
H	-3.78101173717366	-0.15833056835461	-6.55580410377641
C	0.56538435952085	-3.73010861445229	-6.50107827254884
C	2.55995782669192	-2.84605105271848	-7.38726083133557
C	1.33729546932224	-5.96284298850714	3.72962276247534
C	3.27324027903983	-6.68839087681543	2.72161192526929
C	0.71358435861706	6.25178841014562	-4.16214610140742
C	2.65076476345131	7.10379774095166	-3.26043575743130
C	2.81098585161102	3.37883892866226	6.83581344520227
C	0.82611048573873	4.19122170718546	6.00343529404555
H	-0.14271763052164	-3.59239820731189	-7.32224613337096
H	-0.01814686678924	-3.82036195053155	-5.58936988957312
H	2.49328482975257	-2.18552917764687	-8.25244806457403
H	3.51739866021531	-2.64845723788339	-6.90575194918989
H	0.32887490571020	-5.57498176321352	3.60336367776855
H	1.81650243903775	-5.38999262905384	4.54443059655866
H	3.79213686488709	-6.88537570076826	1.78450573909814
H	3.98733739274426	-6.19027143802805	3.40031635072752
H	1.18706980722965	5.68964832034310	-4.98800756705320
H	-0.26421422240269	5.81068113977637	-3.98181916353252
H	3.19658439074420	7.35391973758838	-2.35174458263453
H	3.36279299467549	6.62670662367240	-3.95613306678027
H	3.39650469937616	2.49501700340464	7.08535081113644
C	2.12786786518254	4.02604393252370	8.03956705601838
H	-0.14555283867995	3.96868295943535	5.56801727353505
C	0.77343641540312	4.52582808953340	7.48956412248027
C	1.46478172778349	-4.92190069690389	-6.80029674663231
C	2.43976615691850	-4.32477616529331	-7.80973363857758
C	1.38918081187890	-7.45470319723022	4.04404834573163

C	-0.11914801952826	-2.28131302090331	-1.96877428214109
C	1.02972318550525	-2.57818335592411	-2.71907842045034
C	1.52623624170029	-1.67812690419659	-3.66884361361206
C	0.87663266554944	-0.44884141275032	-3.84396310922862
C	-0.27121743086744	-0.12445044682712	-3.11108004463767
C	-0.71800716800940	-1.04946288982927	-2.17923333657930
O	1.69960760622631	-3.76182737386538	-2.58271841683008
O	1.41678759887848	0.39781599731245	-4.76984428460319
H	-1.60453620153568	-0.80691414519735	-1.61255000848117
C	-1.01919485522316	1.17264362144889	-3.33899144100955
C	-0.51884414472349	2.26690226891843	-2.42009457382792
C	0.42994222503964	3.18040538064457	-2.88074954805214
C	0.92048872220745	4.21061671481405	-2.06315756976271
C	0.42168108626620	4.30979082303278	-0.76470977308034
C	-0.52086036803427	3.41052866480064	-0.26143879720109
C	-0.95250056927006	2.39874535926585	-1.10828756321226
O	0.89014731952890	3.05801938562105	-4.15990275202483
H	-1.66380956132488	1.68098425953055	-0.72685325691354
C	-1.03233237916998	3.53902709784847	1.16043587619493
C	-0.29239388449985	2.61918425408339	2.11428015992759
C	0.82480578412343	3.06128481525520	2.83380836911999
C	1.46722509635535	2.23717900830948	3.76559426123227
C	0.99367914082029	0.93464376154212	3.95157526954271
C	-0.12381735606845	0.45916999874265	3.25139955411148
C	-0.71818037499014	1.31697013169933	2.33727165247785
O	1.34076987668064	4.31740326786676	2.67880034242683
O	1.65584384886077	0.16272235009065	4.86667290691007
H	-1.58899814919167	0.96883451421999	1.80157213732909
C	-0.68275701737001	-0.92656898772488	3.51252109691402
C	-0.09930393351167	-1.95801608346327	2.56702183622031
C	0.96480850851414	-2.77315352149031	2.96097760094830
C	1.51467590688324	-3.73352443498193	2.11239804921573
C	0.96321885035445	-3.87218167890194	0.82836667076225
C	-0.10262367034192	-3.08264185344915	0.40063384702922
C	-0.59180094872802	-2.13016052478224	1.28182834425314
O	1.44701933819592	-2.57501680879920	4.24228664415693
O	1.47433699026029	-4.80146754610407	-0.03071827946722
H	-1.40833545320247	-1.50209651376434	0.95581460566467
C	-0.69175780326366	-3.27656728150449	-0.97899293739142
C	-2.54269844317007	1.02254887031067	-3.28406247641322
C	-2.55597854306800	3.39756667989589	1.23928139683176
H	-0.82766680055701	4.55866540798450	1.47392271700759
C	-2.21383695512911	-0.97324489720731	3.53514083143212
H	-0.37881921632661	-1.21511557118256	4.51484689838342
C	-2.22246401080719	-3.29961376800905	-0.96901807259885
H	-0.38809371826801	-4.26403842386647	-1.31736157451930
C	-3.13335117436433	-0.01378599439391	-4.23728927309357
H	-2.87169614883288	0.78786910837114	-2.27152080517470
H	-2.97301251565023	2.00109600989031	-3.51399909972512
H	-2.98833502684951	4.10661937096202	0.52836079276921
H	-2.86915073741923	2.41033397450595	0.89966484651633
C	-3.16014485557833	3.64779289386730	2.61846100350692
C	-2.87690452860849	-0.05485077514719	4.55812846813325
H	-2.61944925037468	-0.74184616736604	2.55056044811267
H	-2.50749928841736	-2.00607633707525	3.74099921578036
C	-2.84828797978726	-4.34388128887156	-0.04696785807352
H	-2.62051590060003	-2.32365333326013	-0.69265114063577
H	-2.55841001411584	-3.47977909064303	-1.99346506728277
H	-0.79519555231263	1.49966998805185	-4.35097426752121
C	2.63731564012309	2.76392834010614	4.53954598475715
C	2.69022534971727	-4.58913478119762	2.50000413526153
H	1.33661208703529	3.95761522255471	-4.38111172539713
H	1.09480450058273	4.67891877800162	1.80712837397444
H	1.50778089723855	-0.78284961057920	4.68159821981855
H	2.26809036346672	-3.06269159035534	4.37759096583950
H	2.02131057756175	-5.44836378956617	0.55404546662042
H	1.54533336102766	-4.15409939468784	-1.69994985541853
O	0.84322336324115	5.30405183638750	0.09936803589280
C	2.73666910269110	-2.00006344646655	-4.49203277530001
H	1.16218716657263	1.32447512312759	-4.58778586243780
C	1.99452301572243	5.11892387535492	-2.59804962381692
H	1.58428654855688	5.79204393093132	-0.27905885858590
N	2.39730698307840	-2.45433718396091	-5.83810758918554
N	1.76306862016567	5.45723423817677	-4.00087804179599
N	2.23139624523398	3.60878884029407	5.65977392246954
N	2.55841181827692	-5.94664863133369	1.97410974336385
H	3.26087950029240	1.93122768077111	4.89018372047185
H	3.25114885630458	3.37740000864735	3.88196379188266
H	3.60635817713065	-4.16701705577371	2.08122849290238
H	2.83547582283243	-4.63484153227884	3.58722210135302
H	3.34006045114292	-1.10037750443926	-4.60194332541001
H	3.35136294827199	-2.74857397594328	-3.97522336187177
H	2.09518701974198	6.04268845255428	-2.01248712807260
H	2.95996093435844	4.61133752839173	-2.54368256368348
C	-4.66663935496079	3.43240190623522	2.56852416658711
C	-2.82508800263332	5.02893801477879	3.16358822935206
C	-4.39096910234685	-0.18665354573320	4.46430357490487
C	-2.39477783934067	-0.31020445148279	5.97883594192853
C	-4.36688246921331	-4.25672539088986	-0.12574462217756
C	-2.36992216056719	-5.75771049124353	-0.34680701355020
C	-4.64656005113682	-0.06435385186455	-4.07338787667047
C	-2.75066444196890	0.24035427822100	-5.68884372997964
H	-2.73461313809662	-0.99138844591987	-3.95685877617629
H	-2.73953984595222	2.90956768558864	3.30421048094765
H	-2.61679157297877	0.97339285944450	4.29925214889605
H	-2.55414518757902	-4.10241708319659	0.97792498593330
H	-5.14266989843633	4.16294754857258	1.91166171647353
H	-4.91160374488870	2.43898521554498	2.19234276019953
H	-5.11415392803479	3.53434450629288	3.55711919335538
H	-1.75789550715346	5.15208746425555	3.34045180856351
H	-3.14124447313550	5.80892282860187	2.46795076148458
H	-3.33303999569843	5.20438635602215	4.11186198840558
H	-4.74458362393193	0.02156335341685	3.45430140669370
H	-4.71191563127543	-1.19664386524556	4.72649625470817
H	-4.89013401167401	0.50622621050506	5.14149973195345
H	-2.55136010070945	-1.35299792328476	6.26270364710658
H	-1.33610845942155	-0.08613676228576	6.09868567801933
H	-2.94002406368851	0.31143445250482	6.68889837455643
H	-4.71985422115312	-3.25290822282458	0.11276859114639
H	-4.71897837061756	-4.50134136750308	-1.12963192789019
H	-4.83924921622866	-4.95127265530084	0.56899683358454
H	-2.56385108228269	-6.02190162514378	-1.38835046407215
H	-1.30283542208248	-5.87613921026257	-0.16697948150765
H	-2.88910269304403	-6.48261998141062	0.28034338706674
H	-4.92758848486866	-0.27054451196577	-3.04001096523002
H	-5.10098779789257	0.88667683947982	-4.35764515689596
H	-5.08517867897475	-0.84080050727044	-4.70019007592795
H	-3.05010427806801	1.24246518104524	-6.00291069081278
H	-1.67830910442548	0.14602421389914	-5.85033594197905
H	-3.24279311279503	-0.47412080964084	-6.34872701269615
C	1.71053600917390	-3.74035629243478	-5.89662687339576
C	3.56643301878812	-2.64449233949399	-6.68782905370903
C	1.51723531534559	-6.75757605318052	2.61837306787461
C	3.77544330909779	-6.76005286937178	2.06666489535925
C	2.90200144268376	6.09928630165476	-4.66650008173946
C	0.63495250214052	6.36853311158508	-4.23402005948095
C	1.53986721925004	2.90604354761949	6.73603331113197
C	3.35743830401280	4.24838342612843	6.32994662432653
H	0.70413318033660	-3.66326010771955	-5.49022000276032
H	2.24838026839995	-4.50091257517557	-5.30754437368323
H	4.02878454635298	-1.68731357971670	-6.92757920733727
H	4.32753659812643	-3.26290754000001	-6.17941151633500
H	0.53020207808995	-6.36896438901597	2.38019654289292
H	1.64151038132354	-6.72537261996233	3.70846121074188
H	4.52640374541123	-6.39144315168998	1.37129404476835
H	4.19085712388757	-6.69725076498690	3.08040273962916
H	3.28199576147457	6.92000895401709	-4.04499231096305
H	3.70763207724177	5.38203760171753	-4.80736093244931
H	-0.30713776432889	5.87520364732613	-4.00850691315614
H	0.72601494262968	7.24514222806547	-3.57978365687413
H	0.55562644072196	2.57151801083621	6.41440894696027
C	1.50356522880592	3.93445034539947	7.86208093256811
H	3.81117359173752	5.00212096683876	5.68710058070864
C	2.74553433096032	4.81810145514343	7.60868806506738
C	1.74891096284816	-4.09310297122103	-7.38024733813535
C	3.01713718965783	-3.38468941449125	-7.90652141733355
C	1.77865412208899	-8.15534239330089	2.07454393222676

C	2.03159121461737	-6.63191160319364	4.61967718886464
C	2.78857571994853	6.64205135105234	-5.13615583813351
C	1.29758621206331	7.00795299189636	-4.95700433935261
H	1.09850670826089	2.83366706895785	7.17049161122071
H	3.58785755115130	3.36277508974409	6.54573148849347
H	0.89786226250566	5.04682170549695	8.02267052621219
H	0.47387381700857	5.71217169183391	6.45634199907723
H	2.69598259717256	6.20190129705045	6.04881994174426
H	3.19388834492264	5.45166781668241	7.55229942428041
H	2.90609931053982	-5.67818260947780	-6.35834953891868
H	3.28916281583629	-4.93106302921006	-7.89744814129082
H	0.95477456564165	-4.71645824970433	-8.30289930972920
H	0.64139362147933	-5.42060050218060	-6.72787573585075
H	3.65995068894201	-5.54957316384685	5.58617514772582
H	4.18453962471871	-7.04271659019789	4.83366062771933
H	1.90168993325095	-7.70499374955665	4.73637245945536
H	1.43563138501851	-6.14496512424669	5.38866398737082
H	2.92472961348171	6.00325973572294	-6.00584939959597
H	3.41618471982990	7.51880536964470	-5.27577468007247
H	1.11334074315038	8.07436273161839	-5.06085295016708
H	0.68804775958085	6.49722960169937	-5.69942079029454

C	2.69943764200725	-7.93891405798589	3.38371695847082
C	0.66641051246935	7.73671274439602	-4.50608975868383
C	1.98225579387164	8.30375598212077	-3.92796839615738
H	3.48867198165110	4.10005967667578	6.34717169738104
H	1.25897106223612	5.03934967891912	5.44164216542034
H	2.73319905523803	4.83219340108589	8.44829120103952
H	1.97536991194813	3.29879817939613	8.83408343034253
H	-0.05099930998071	3.99859407446689	7.96523299832720
H	0.61961293575387	5.59014521213069	7.65318677018510
H	0.92007702440136	-5.78174344784226	-7.18700291626958
H	1.98808004276758	-5.24052760688578	-5.89717915326685
H	3.40478078753573	-4.82780132076056	-7.82148639775214
H	2.02177932155150	-4.39637923227590	-8.81371120521494
H	0.53047232811759	-7.96408775594773	3.61161772823423
H	1.36926106429159	-7.63412814541716	5.11670246063699
H	3.39749345582428	-8.36101995905877	4.10334498850550
H	2.49288057988871	-8.70581421161008	2.64032850648481
H	-0.19379979799577	8.20770997214507	-4.03487293273821
H	0.57743110602192	7.89278615507167	-5.57890858664409
H	2.62175595489429	8.73572348278589	-4.69462347229493
H	1.77550048708366	9.08348215211882	-3.19810515413144

C	3.29237715257693	-8.17735066128549	1.76377869851915
C	2.31202837489530	6.63314262942418	-5.97011712895232
C	0.79586504970328	6.75686731437310	-5.69747935119367
H	2.10731661818863	2.01463260489714	7.04970844095391
H	4.13892489166015	3.50883471066197	6.57862362878434
H	1.52135107829130	3.45617692429419	8.83933158629987
H	0.59354945917489	4.52887355777334	7.80541142566559
H	2.45540521929078	5.85718486964228	7.46617110742033
H	3.45451564520766	4.78845033210980	8.43372621767895
H	0.86191783465095	-3.71432896026617	-7.88478571036805
H	1.77635770317610	-5.17023416436266	-7.53274076336170
H	3.74860642036009	-4.08324877384502	-8.30794975919519
H	2.76493404307913	-2.68457537577736	-8.70050327886870
H	1.20090277490777	-8.31841590399774	1.16737609608169
H	1.48660359075415	-8.91985478463610	2.78997725811140
H	3.82736019883887	-8.90646916198734	2.36700749046951
H	3.46576387325823	-8.42848422126518	0.72007262691906
H	2.49961927272912	5.94272905440332	-6.78901721229753
H	2.76262224677622	7.58545619002701	-6.23806014898975
H	0.41734050076009	7.75855893357831	-5.88475196126820
H	0.23732579952759	6.07218523365990	-6.33187478858885

6

C	0.18302944054556	1.32423833645554	-2.94698962269748
C	-0.94565128530065	1.35237611247084	-3.76697660129097
C	-1.52844059725094	0.17714888838745	-4.25440889089372
C	-0.95932023803192	-1.05383836588008	-3.91117507596329
C	0.19127433492030	-1.11473688579636	-3.11301103523069
C	0.70547319392252	0.08106893710586	-2.62739076261167
O	-1.48807965087696	2.56326189154079	-4.10174259266301
O	-1.57575055377625	-2.16798420680871	-4.40818269127567
H	1.58155561188679	0.03573558617394	-1.99729809559252
C	0.86091139126582	-2.43716089765781	-2.78717548031211
C	0.29569961694166	-3.00456460723564	-1.50229863191629
C	-0.75283572466642	-3.92323186552762	-1.54612290862444
C	-1.32420155657320	-4.44320705014283	-0.37960560149292
C	-0.81230149086314	-4.04504880325074	0.85932225849259
C	0.27111977920229	-3.15883451569314	0.93628563982379
C	0.76511789809869	-2.63505633805716	-0.25168921720057
O	-1.22894985529614	-4.32226766430318	-2.76594352421910
H	1.58139339660515	-1.92933141528182	-0.19559180070498
C	0.90532257237067	-2.79501467002239	2.26608058034303
C	0.30663889935375	-1.51400095118626	2.80565787964305
C	-0.78888857945651	-1.56668333509085	3.66796251106778
C	-1.38211306227951	-0.40434687212148	4.17344932318635
C	-0.85463949117974	0.83882317059730	3.80875404207254
C	0.26458891240424	0.92461548951904	2.96926415702529
C	0.78764356570808	-0.25961868062187	2.46554983092082
O	-1.28825997537090	-2.78909976325559	4.02708129862584
O	-1.47774212245196	1.93973224496912	4.32674218578946
H	1.63736350712931	-0.19543486485963	1.80193423028250
C	0.89457433176874	2.26074161657594	2.62208180103921
C	0.27725850150479	2.81671437449408	1.35649967631613
C	-0.78674073071294	3.71528603137768	1.43504777672383
C	-1.40056663870196	4.22974667687363	0.28775529445986
C	-0.92117047802139	3.84167448023539	-0.96732554261039
C	0.17300810817188	2.97286843846344	-1.08000030392358
C	0.71389513456820	2.45747522448700	0.09113273076379
O	-1.23344369443324	4.10121302109755	2.66993722304910
O	-1.55943712747144	4.36606432321044	-2.05714770670415
H	1.54263181146467	1.76915426537545	0.00757248582452
C	0.77128846368992	2.61914171257456	-2.42925516611181
C	2.39228035964707	-2.34048603797029	-2.81958031595830
C	2.43902904890279	-2.80473184264868	2.19361290105522
H	0.64486893264670	-3.58685753788067	2.96790962626918
C	2.42803043855913	2.19625142515966	2.60599325603445
H	0.64301091758099	2.94980572158814	3.42799362471179
C	2.30602032358279	2.66202025334022	-2.40557992068810
H	0.47216005584805	3.40292160574167	-3.12473786280740
H	2.66126716371814	-1.63036901258434	-3.60254667744162
H	2.77867569395373	-1.91704083307839	-1.89122151820677
C	3.12366694885293	-3.65020639165523	-3.11246720233274
H	2.72911159260571	-3.58852927644484	1.49274811652923
H	2.81920293711266	-1.87480587333397	1.76841184235221
C	3.15323239220991	-3.07952564289075	3.51638175807709
H	2.73582785120734	1.48930846027696	3.37752084151255
H	2.79403874714222	1.78394061318486	1.66455198348483
C	3.14003380679623	3.52004491931822	2.88121430948973
H	2.60032011896286	3.45767804996000	-1.71991582341890
H	2.71930227935332	1.74397546634609	-1.98519904453518
C	2.97306726342669	2.94159756412632	-3.75155467561004
H	0.59793541289195	-3.13220249028832	-3.58436620493612
H	-1.78140391575511	-5.17838428071846	-2.57279354463467
H	-1.89479694474786	-2.60415759043374	4.84892607647835
H	-1.32741361148177	2.73144199019551	3.76532417158798
H	-1.80646179987954	4.94836999365651	2.49694611512049
H	-1.48299241225012	3.75757160020383	-2.82501142594332
H	-2.12889635421698	2.36518826623158	-4.89378714770887
O	-1.40769325453753	-4.57777031735208	1.96905067073638
H	-1.38630252187188	-2.95713457979452	-3.85485800010863
H	-1.31073584198320	-3.97246746116790	2.73705968198925
C	2.88298972025976	-4.74186153023977	-2.07922015806373
C	4.61611224570500	-3.38106321895115	-3.26260580753725
C	4.47403915965422	3.11656500718272	-3.55404425501255
C	2.69804612941346	1.88729688332039	-4.81453344734257
C	4.64120690357580	3.28143827128392	2.99054867191165
C	2.84901119096607	4.60760567020132	1.85641514319685
C	4.65159741244783	-3.21276614297645	3.27272362479823
C	2.88147735977750	-2.04268120917306	4.59727472869176
H	2.75250897708487	-4.01733711607819	-4.07604137176870
H	2.78800735086461	-4.04531817201622	3.88367556601352
H	2.78763417248974	3.87845619296635	3.85502616510921
H	2.57221750708031	3.89394413888490	-4.11700952227166
H	1.83555340526049	-5.02627176328015	-2.02084100484905
H	3.18602667165540	-4.41125653784935	-1.08497429672256
H	3.46032700173329	-5.63382317755989	-2.32538410407183
H	5.03976022510512	-3.02077387710355	-2.32326557968393
H	4.81175361652034	-2.62726911876856	-4.02566616072552
H	5.15459372701897	-4.28733120131691	-3.54013092420068
H	4.92734022436080	2.18929022144949	-3.19858809262563
H	4.69296441534630	3.89469323648352	-2.82238947706216
H	4.96644026773796	3.38625062666210	-4.48845978361801
H	1.63782560988690	1.79604759262740	-5.03633890275407
H	3.04824611194634	0.90654126547173	-4.49003854189033
H	3.21614509423021	2.13639533637622	-5.74142695035370
H	5.04712034991774	2.93198798369496	2.03934928644384
H	4.87259568511733	2.53000096974850	3.74590935646374
H	5.16821679306547	4.19790209377859	3.25635294482928
H	1.79517303555332	4.87252766770572	1.82881021072669
H	3.12878669459286	4.28287664039665	0.85342072009912
H	3.41657393795098	5.51011831676091	2.08629688672613
H	5.06976443762655	-2.27038490716971	2.91398199889790
H	4.86882222702127	-3.97747278871944	2.52653350327627
H	5.17850268170614	-3.47879436360369	4.18919851639427
H	1.82756768169361	-1.99073414015916	4.85860061700813
H	3.18390161806064	-1.04785838373481	4.26788296630238
H	3.44158857224373	-2.27960825993079	5.50262713662411
C	-2.78716898177259	0.24534707577007	-5.07267459314570
C	-2.50353842989946	-5.36888236691148	-0.47763054073914
C	-2.60568311348910	-0.50422406862582	5.04018445726655
C	-2.58757021195944	5.14100114776520	0.42290551787409
N	-2.53929259285082	-1.66990317261930	5.93970378746546
C	-1.55892643961106	-1.46847915665183	7.01463584013935
N	-2.36823581868525	-6.29333202609880	-1.61828060809909
C	-1.33433715953667	-7.30620133414215	-1.36912709066550
N	-2.79625230518434	1.42047112358841	-5.96134515016125
C	-1.85360885063685	1.26238601843626	-7.07694300106420
N	-2.43241207085123	6.05925200914926	1.56584894108722
C	-1.41402817158246	7.08336476492451	1.29981300382986
H	-3.64693951673568	0.34423820601252	-4.40579384796427
H	-2.93386195929999	-0.67441972245567	-5.64110302105318
H	-3.41110856591646	-4.78568688179685	-0.64888986405570
H	-2.64948481367548	-5.91677151331900	0.45447108805019
H	-3.48717715923567	-0.64092975834951	4.40961255841868
H	-2.76086117691174	0.41621611692677	5.60508841362701
H	-3.48397616128348	4.54669946280660	0.61360795799395
H	-2.76344463204604	5.69333571565862	-0.50138840472726
C	-3.70457429170033	6.66326834761370	1.97093673277262
C	-3.85376346677973	-2.03421165589623	6.47550862128597

C	-0.06055994881292	1.70750688107400	-2.75727351183080
C	-1.14252242076237	1.84747656396377	-3.62682911638586
C	-1.70638869818414	0.76228440410807	-4.29554310050593
C	-1.16101732478094	-0.51129417665808	-4.07038633587545
C	-0.05179570957054	-0.68782058313911	-3.23574562023512
C	0.44533782519943	0.43050879471617	-2.57990379051074
O	-1.61769493560620	3.13184238163915	-3.81061043722100
O	-1.75316115389548	-1.54740067620910	-4.72998539957467
H	1.29495268987467	0.30109128880295	-1.92629592745325
C	0.61931147314954	-2.04129849627841	-3.08356986723290
C	0.07552030885667	-2.78045736983976	-1.87742077377259
C	-0.96042768994002	-3.70478333393427	-2.01353544670370
C	-1.48247311665216	-4.40486764622896	-0.92701603412940
C	-0.93777316582781	-4.16036873186273	0.34308820048969
C	0.12748338412767	-3.26912704904578	0.51583547844100
C	0.57976130170657	-2.58225902414080	-0.60284174281970
O	-1.43414455443402	-3.90839138263320	-3.29520127740326
H	1.39458970647495	-1.88519823329513	-0.47722761341353
C	0.79682572050692	-3.08103765142594	1.86637525973226
C	0.18292907905776	-1.90795132574215	2.60376924281029
C	-0.85073109055205	-2.09991931549149	3.51944127293072
C	-1.45639193325726	-1.04436706687129	4.19883239644573
C	-0.98551172793373	0.25321166312603	3.95117003247432
C	0.08277757103985	0.48498386289157	3.07627196908034
C	0.61152021154579	-0.60636623864892	2.40159374677862
O	-1.24155536163368	-3.40957039368149	3.73395100183850
O	-1.59639430822367	1.26307356823879	4.63848765672348
H	1.42767070911226	-0.43434071715927	1.71630632218546
C	0.67992144973959	1.87028071707734	2.89758478524438
C	0.03691303969718	2.58449746907083	1.72569371833070
C	-1.03052359350030	3.46045152778333	1.92171471089367
C	-1.64603979550640	4.13515045449131	0.86818939672131
C	-1.16754195882309	3.91032293640366	-0.43181480280020
C	-0.07337557507934	3.06938249398266	-0.66566328476071
C	0.47550259364515	2.40709530051351	0.42393079272304
O	-1.43863197444054	3.64383974430703	3.22900900326198
O	-1.80822458557659	4.57399450753512	-1.43596646687555
H	1.31250964871986	1.74791775551205	0.24873036151770
C	0.52701400068763	2.91288564496608	-2.05159576110998
C	2.15108317720068	-1.92933224446465	-3.12104314761249
C	2.32631513192878	-3.02785480461899	1.74331299154601
H	0.59432050555255	-3.97755198758811	2.45022412546380
C	2.21457381461123	1.83167293279608	2.85246038189699
H	0.44167587422641	2.43780791303731	3.79605375633854
C	2.06248491656848	2.94374577128301	-2.01899850858308
H	0.24319300016313	3.79525002501474	-2.62322400111235
H	2.40138504441190	-1.12555301653725	-3.81435497941112
H	2.54904874161377	-1.61933840611687	-2.15399117500420
C	2.88814197232370	-3.18578014338303	-3.58477546327236
H	2.61037951320757	-3.72585463312520	0.95482278130173
H	2.66078887243692	-2.04714541233718	1.40213803887048
C	3.10291009636057	-3.41186760827675	3.00282788297793
H	2.53870958015516	1.04570684088480	3.53529845066364
H	2.57328503983255	1.53197873222782	1.86690567764946
C	2.91688223651761	3.12428306574295	3.26818607470288
H	2.35704084893941	3.63742844533800	-1.23079056350680
H	2.47197026075097	1.97567980869692	-1.72819575724476
C	2.73505521462338	3.40128255924289	-3.31354238113330
H	0.36203673976267	-2.62493752017886	-3.96625976093243
H	-2.19303423306676	-4.50712531085136	-3.27577864272543
H	-2.04475494371943	-3.43137633034740	4.26867044682770
H	-1.48670154638962	2.11195429744774	4.16569234566555
H	-2.23840880037475	4.18691727232535	3.25184715364800
H	-1.68503246128454	4.10345997299247	-2.28370232581281
H	-2.41148763257632	3.11402079865830	-4.36225157429726
O	-1.48650043138560	-4.85537958734916	1.37972457697189
H	-1.58401326067127	-2.38804373120518	-4.26099419319653
H	-1.33504370004614	-4.38134115736070	2.22161731681118
C	2.67600047967959	-4.39923819117037	-2.69045044062702
C	4.37535179538985	-2.88251229801038	-3.72263931531917
C	4.23531459735590	3.54233957106564	-3.08584014244107
C	2.46330757492220	2.49918437174601	-4.50942227235969
C	4.41919101113605	2.88255750905123	3.35411087107004
C	2.62335808701244	4.31305057248678	2.36352445945580
C	4.58985760501474	-3.49514497668425	2.67833062092165
C	2.87235108685949	-2.48328244074434	4.18689699157280
H	2.50446816480009	-3.43348251123891	-4.58075883794415
H	2.76925631534893	-4.41408096496428	3.29412894404120
H	2.56135763811700	3.37403127144882	4.27404448399713
H	2.33747656568412	4.39510475404365	-3.54736518485762
H	1.63351473051531	-4.70389305535892	-2.65114886610529
H	2.99334563754507	-4.18946916270997	-1.66831799504631
H	3.25852458009461	-5.24629867257798	-3.05408734322150
H	4.80978124436593	-2.63400222891335	-2.75251978310646
H	4.55120938588723	-2.03915034649062	-4.39074583910206
H	4.91803102751244	-3.74173096738504	-4.11648458450248
H	4.68124629078633	2.57518616427299	-2.84634763745827
H	4.45244179372317	4.22105708634646	-2.26080902611170
H	4.73511266352202	3.92454062894809	-3.97574969437431
H	1.40634990928347	2.45227131268378	-4.75811685441270
H	2.79765896235997	1.47997272297440	-4.31168958545578
H	2.99795782297517	2.86128506119689	-5.38811608950034
H	4.82577520354349	2.62724851876342	2.37371393919118
H	4.65409250139524	2.06369753097969	4.03431985596611
H	4.94195894509331	3.77191739835315	3.70573499133503
H	1.56842733694319	4.57395070572915	2.35496627482348
H	2.91466838220758	4.09963845552919	1.33437687945627
H	3.18219299165728	5.18932221468260	2.69358252038995
H	4.97676144501253	-2.51724327595257	2.38553711477210
H	4.78249743784394	-4.18765247180529	1.85869853435051
H	5.16301997038572	-3.83020921968661	3.54270102821640
H	1.83560235556048	-2.47979240622867	4.51312562843133
H	3.13628242108666	-1.45630088558997	3.93180019570932
H	3.48893498957278	-2.78659219892291	5.03353836568069
C	-2.84979837577476	0.90261370828059	-5.25432579271805
C	-2.58637939018836	-5.40940007517240	-1.06436134229726
C	-2.55896645866002	-1.26519061178625	5.19887883415628
C	-2.78174527842412	5.09042232874069	1.07536061798309
N	-2.06731347760196	-1.46528783395133	6.57403204889268
C	-2.46180943250496	-0.37751762833950	7.46579343320611
N	-2.11436987069220	-6.79035689752919	-1.06417251855859
C	-3.22860666996541	-7.72246810466423	-0.94050682246890
N	-2.43086275956996	0.88413750355060	-6.65214967702869
C	-3.57785921444052	0.74861800107003	-7.54174665079333
N	-2.37682444106809	6.49348533397144	1.06073823087224
C	-3.54793400449933	7.36245171965768	1.09944181247779
H	-3.45193700664304	1.80634552590159	-5.03794037835457
H	-3.52924199925362	0.06624537609308	-5.10705724527290
H	-3.20560102425596	-5.20732166119930	-1.95990501650796
H	-3.26283773869447	-5.29690611545616	-0.22002148375278
H	-3.18872825229492	-2.11241335042013	4.90956931601332
H	-3.22908645948438	-0.40767868016817	5.16942632041483
H	-3.33719457720226	4.85791064642901	2.00530412130788
H	-3.50271192222819	4.95240749458675	0.27252055457376
C	-1.44990884376455	6.81715978295083	2.13802188880754
C	-2.43400965365097	-2.76039907960194	7.13430173732696

C	0.11690120236486	-2.21489112854750	-2.14009166080258
C	1.27566548218628	-2.44469252925291	-2.89821383098896
C	1.75234244306569	-1.48638306661900	-3.80040085856424
C	1.07442326043055	-0.26517961100569	-3.91462562588218
C	-0.08403973587109	-0.00678801354606	-3.17223711486376
C	-0.51173641926026	-0.98912813491565	-2.29134953696294
O	1.97572923282743	-3.61581645541363	-2.82071787928119
O	1.60056767446401	0.64059003958255	-4.79010150858781
H	-1.40652437465113	-0.79807674327405	-1.71857560898152
C	-0.86000411145118	1.28378930648560	-3.33542309969535
C	-0.37447040801448	2.34353782410594	-2.36933504954172
C	0.54697973017779	3.30034618411270	-2.79576464391683
C	1.02542831775684	4.30033552075016	-1.93471468360170
C	0.53968516548520	4.32601703138021	-0.62799397221922
C	-0.37492481894383	3.38085170659260	-0.15810434865092
C	-0.79398521237024	2.40057973063592	-1.04750281296928
O	0.99136672277009	3.25473281614929	-4.08508890735639
H	-1.48334901763015	1.64816304530673	-0.69291778018651
C	-0.87089387783351	3.43075323044575	1.27413514874829
C	-0.10574165807171	2.47639527484844	2.17231560740842
C	1.01359024539332	2.90091356378353	2.89923690047158
C	1.68096093484279	2.04214186682789	3.78124371697410
C	1.23492842057485	0.72200244416625	3.90138976160759
C	0.11404660008887	0.26421479977691	3.19495313253844
C	-0.50816547237803	1.15786813897365	2.33532959458195
O	1.50775745796191	4.17117390871662	2.79951516711794
O	1.92636820041580	-0.08471997390449	4.76294970629941
H	-1.38269983322259	0.82304994526913	1.79704234644231
C	-0.42051180717125	-1.14081763428657	3.39745245949905
C	0.16835262167057	-2.11890635924519	2.40034652107376
C	1.24306269450974	-2.94096587554363	2.74862011440939
C	1.79928390051000	-3.85183828942372	1.85118397602711
C	1.24082424666342	-3.93393604445203	0.56493641916736
C	0.16371456019499	-3.13676178120564	0.18228146660907
C	-0.33000888484988	-2.23305638972481	1.11105442108539
O	1.73150927077991	-2.79845804119972	4.03501743778011
O	1.75710255182070	-4.81551889081087	-0.33924735101262
H	-1.15380163045381	-1.59692606574883	0.82052586967777
C	-0.43119778505264	-3.27127805302655	-1.20199308674006
C	-2.37946271173372	1.10040655810324	-3.27958892506527
C	-2.39147221543688	3.26451367868309	1.36377081776952
H	-0.67566195242052	4.43675492351872	1.63339082397471
C	-1.95046332259507	-1.21371516529122	3.43373929771957
H	-0.10219865860562	-1.46912557503428	4.38297157150392
C	-1.96095310337079	-3.32566509607071	-1.18353009552763
H	-0.10980360829654	-4.23390326964554	-1.59128481221762
C	-2.95517533081715	0.10694922500447	-4.28640777476284
H	-2.69577811174978	0.80287089980232	-2.27960450277482
H	-2.83114157073772	2.08093108803079	-3.45268770532471
H	-2.84272229347311	4.00022168597075	0.69286920534377
H	-2.69442059346651	2.29012430433261	0.98096126426264
C	-2.98038972407547	3.43984710911820	2.76117745974277
C	-2.61791092873583	-0.34405637988770	4.49585039773367
H	-2.37062523987123	-0.95472754188352	2.46231035277376
H	-2.22413598042386	-2.25826260159822	3.60524043409680
C	-2.55943776110400	-4.42814523930908	-0.31229742118841
H	-2.37698972241188	-2.37424224964748	-0.85360775232865
H	-2.30080412450619	-3.45907793261111	-2.21387733533593
H	-0.64830311335236	1.66106873096559	-4.33242735815707
C	2.85432710684396	2.54821938330031	4.56235503103029
C	2.99232892274334	-4.70570938300315	2.18608107762224
H	1.41252947233348	4.17706357692891	-4.26487629914532
H	1.23967349960724	4.57308824580532	1.95261804817612
H	1.78241142039159	-1.02260104168315	4.53970698211862
H	2.55715947098031	-3.28535766270382	4.14254347482891
H	2.31458979555288	-5.48327657904096	0.21724251401079
H	1.82495681925676	-4.06607702435804	-1.96512008711494
O	0.94773934594675	5.28771042528400	0.27821759454773
C	2.97934727116630	-1.72903833443774	-4.62480867676312
H	1.31040615402576	1.54939489314912	-4.57393681831204
C	2.07453412067965	5.25348641675261	-2.43735392108752
H	1.66756554428135	5.81562876236454	-0.08753975356681
N	2.68131326219695	-2.06525240451629	-6.02267836913888
N	1.81992968387739	5.66294887073686	-3.82460156937477
N	2.46485114220162	3.35698099749408	5.72459990149967
N	2.88657939747431	-6.04300827927875	1.58756001210299
H	3.49042754044545	1.70792847927326	4.86748958609798
H	3.45295848809744	3.19079087355776	3.91917041748045
H	3.89500787122330	-4.24401799080771	1.78158984380525
H	3.14912559895245	-4.80086668983923	3.26802062024755
H	3.57495607666579	-0.81755967213765	-4.64236150609718
H	3.58997793420140	-2.51155362055099	-4.15788971482477
H	2.17290728421224	6.14489498541313	-1.80435797565385
H	3.04744629650384	4.75854065690960	-2.42400557559645
C	-4.48232829679067	3.19374850566765	2.72234437769411
C	-2.66740180904926	4.80200835310220	3.36436915003695
C	-4.13033774107220	-0.50101157297348	4.41625626131863
C	-2.11252699686500	-0.63750245652462	5.90096041857704
C	-4.07998263634882	-4.36331106500460	-0.37245900367581
C	-2.06052775115105	-5.81586622664134	-0.69103254830452
C	-4.46458299841899	0.00701527732089	-4.10941314758045
C	-2.59730207456909	0.45847620924160	-5.72395278290562
H	-2.52905594349465	-0.87518406912438	-4.06876098928233
H	-2.53283051217583	2.68117647197480	3.40632023947251
H	-2.38037007102101	0.69703985353537	4.26939244042863
H	-2.25795762794911	-4.23576576101795	0.72083786290534
H	-4.98200428945011	3.93822747345308	2.09957830230443
H	-4.71142046828566	2.21027975429664	2.31170049930771
H	-4.91891000918468	3.24883934485092	3.71950296007652
H	-1.60013994502857	4.94496765812632	3.52474924994816
H	-3.01937623942507	5.60615913833591	2.71489763351064
H	-3.15848381076500	4.91803750739264	4.33044595325215
H	-4.50095204100923	-0.26542945187799	3.41842885280628
H	-4.42963843883714	-1.52496475090573	4.64815187142980
H	-4.63307964075342	0.15930871716761	5.12272022959094
H	-2.24722987236890	-1.69150204559363	6.15295102059458
H	-1.05630867331141	-0.39886375944697	6.01447510042011
H	-2.65869994899016	-0.04897943258171	6.63802089952376
H	-4.44753852759021	-3.37890601956461	-0.08108763229134
H	-4.43722624469057	-4.56403419842343	-1.38423474013532
H	-4.53376715596466	-5.09996768877776	0.29057254238275
H	-2.25964569479547	-6.02728110850287	-1.74351251319524
H	-0.99019130918565	-5.92551134846959	-0.52636756283992
H	-2.56202838330524	-6.58173014213813	-0.09917451332550
H	-4.72629917421466	-0.27277987476247	-3.08840504485918
H	-4.94676450435234	0.96212306769268	-4.32626062228058
H	-4.89157315458287	-0.73877900260278	-4.77995596640378
H	-2.93006343014417	1.46844843893427	-5.97199672817936
H	-1.52474364385984	0.40615922489954	-5.90247805976498
H	-3.07683546629485	-0.22896072014382	-6.42074049268139
C	1.96990509585007	-3.33171380439171	-6.14580610156403
C	3.90361383076910	-2.07908203191814	-6.81542262722666
C	1.84821335568377	-6.85445559544084	2.23760665940323
C	4.17465605165406	-6.74362848135700	1.55621906178672
C	3.00058235019508	6.27557878430159	-4.44267253961925
C	0.64368030014355	6.53761736705679	-3.92916704204805

C	-3.64480474185285	-6.91191871239967	-1.98608685674793
C	-4.14304202548631	1.74394885368642	-6.43998227133547
C	-1.16768597850590	7.93510400222613	2.52834520912413
C	-2.45997563055922	8.57263544494959	3.01409246113705
C	-3.52833002861018	7.50968076225096	3.21707765267511
C	-1.40390440478021	-2.72390248417471	7.84872941356156
C	-2.74354630080556	-3.16839610138137	8.41486809134878
C	-3.76823945439450	-3.30441437613462	7.29926523042063
C	-1.11215689180838	-8.16878385228206	-2.59466759623189
C	-2.41108130327723	-8.82205905997550	-3.04014561712047
C	-3.49256804667399	-7.76955949095667	-3.22764580409176
C	-1.77604258755589	2.52961155133265	-7.90393731314404
C	-4.13490505416211	3.02229397494664	-7.25540501653717
H	-1.87798522787864	-0.63008634508838	7.64986303938540
H	-0.61240249703137	-1.18629081113506	6.55743880378516
H	-1.63332201577480	-7.93229326071206	-0.51654287812587
H	-0.41924007854550	-6.78959315744940	-1.08614386463454
H	-2.16890788699608	0.41888181261369	-7.70721430997480
H	-0.88022144486655	1.00934100756191	-6.66074126030244
H	-1.73964454023946	7.71517731787560	0.46128111480871
H	-0.50214117945127	6.57745536660616	0.98857173051292
H	-4.41653499951900	5.85910834926646	2.15210037800345
H	-4.10466165049483	7.27739385716807	1.15126057106621
H	-4.53347973230733	-2.16698263143612	5.63498389043965
H	-4.24858307143203	-1.21199246497893	7.08961540095085
H	-4.36769138299773	-6.11537752526828	-2.15746986965086
H	-4.01944236067181	-7.52089435675911	-1.15069420324014
H	-4.79196257814944	1.84695378983156	-5.57146498854005
H	-4.53456955929374	0.91338320177686	-7.04489427683822
H	-0.42252819502351	8.69493991638533	2.29088834531846
H	-0.74461738023053	7.30827750730088	3.31651343423564
H	-4.48517187094536	7.96341177800864	3.47736458089260
H	-3.24580668024294	6.85888216036506	4.04758355447053
H	-0.98441808443129	-3.51623528619857	7.22475201666774
H	-0.68662577692486	-2.53450700940962	8.64792581727617
H	-4.75500006121749	-3.53468247293478	7.70227893006230
H	-3.48890783790132	-4.13154743655811	6.64285446357876
H	-0.35504867030861	-8.92006662377835	-2.36799786505339
H	-0.71429828604789	-7.54761247606604	-3.40025530008185
H	-4.45192334842086	-8.23332553373369	-3.45941394068948
H	-3.23572384009976	-7.12541221068993	-4.07153902399048
H	-1.08476605242970	2.37245390110898	-8.73250279799290
H	-1.36016121279283	3.33095743383923	-7.28918266347789
H	-5.14459171622865	3.22048287133764	-7.61653741809585
H	-3.85858139459953	3.85422339743233	-6.60379603887208
C	-3.15192419083201	2.93074701061988	-8.41234849090425
H	-3.50416663187547	2.18129573666072	-9.12674299821737
H	-3.10249053485514	3.87892978236145	-8.94869652788341
H	-2.80687482396272	9.29550226236532	2.27032530659971
H	-2.29213484107174	9.12741243564449	3.93787232101505
H	-2.63926169971190	-4.10959455349585	8.95572900890016
H	-3.09345538014390	-2.42584094758209	9.13756858923332
H	-2.26228860845163	-9.38610787979612	-3.96156911876003
H	-2.73309379922473	-9.53884479359828	-2.27954679052825

C	-1.29845313206904	-7.09812263076487	-2.23111323806447
C	-1.62516179340925	2.04505743688384	-7.00574117582470
C	-3.15989306373027	8.82402182187547	0.97866852814991
C	-2.16295385624969	9.21418865159615	2.05838615792703
C	-0.98200865097506	8.25714337835067	2.05044641792869
C	-1.80402653464417	-0.51430720455059	8.82587418349762
C	-2.11798040706652	-1.86393578084977	9.45089592894745
C	-1.77244800988194	-2.98307291699936	8.48185177030438
C	-2.73879244246360	-9.15306177124462	-0.81766633757660
C	-1.85533696310736	-9.52873693244966	-1.99663238211462
C	-0.74188614805927	-8.50755207620635	-2.16229872645443
C	-3.13784704964785	0.60201485916007	-8.98631035965736
C	-1.11773331522843	1.95354154024715	-8.43236798540175
H	-3.55940900050487	-0.35696956632835	7.59561612234623
H	-2.17671893968803	0.56261489445480	7.00167186776241
H	-3.90662810510955	-7.64324190543369	-1.81077265956922
H	-3.80957468933074	-7.44455830059596	-0.06080777707918
H	-4.25107134915028	1.62158635465779	-7.45274497597298
H	-4.14994158717304	-0.12558741935399	-7.23015903358928
H	-4.11164856384508	7.21122444169351	2.03876021869845
H	-4.21099413177310	7.07759292671350	0.28224301598535
H	-0.59907037737405	6.14206422173664	2.07603726946579
H	-1.92591071263321	6.64965112840006	3.12350542137252
H	-2.12035997724091	-3.54610666578839	6.44685312566168
H	-3.53002827604969	-2.85067997436782	7.24634626681213
H	-0.48317931389637	-6.37904215018704	-2.28371352751676
H	-1.89161307983459	-6.98404242012490	-3.15915351811512
H	-0.78579301657091	2.10748951251791	-6.31582868691616
H	-2.21322868068569	2.97542164405075	-6.88573879140475
H	-4.05871722142852	9.43956652525785	1.03861738927501
H	-2.71943038572760	8.99251129702555	-0.00653979763711
H	-0.30647335015146	8.46531389080303	2.88140857252182
H	-0.40919834768607	8.38581222423502	1.12944414153656
H	-0.72338349312124	-0.40530026365318	8.70804035014699
H	-2.14035784244974	0.29979020944702	9.47008340007508
H	-2.08222959629257	-3.95125551666070	8.87849227524208
H	-0.69043843446380	-3.02362960701377	8.33758381512586
H	-3.59729855768079	-9.82249978505538	-0.74498509315205
H	-2.17307921783260	-9.25243500504138	0.11130357079594
H	-0.16014534964093	-8.70703435252711	-3.06342704708181
H	-0.05470999257237	-8.57056970443256	-1.31558452944024
H	-4.01870696038941	0.52002205708904	-9.62491089517485
H	-2.57681752258261	-0.32936066587511	-9.08959156461324
H	-0.54274245416123	2.85081436879305	-8.66600030707569
H	-0.43365826887277	1.10518494358136	-8.50625961783084
C	-2.26642470046319	1.77310205313725	-9.41141763349004
H	-2.87130828502079	2.68457879851593	-9.43021160205476
H	-1.88994507973431	1.62575196030904	-10.42466964855265
H	-2.65377985793093	9.17067893158387	3.03510843875175
H	-1.82794221959392	10.24313529777386	1.92033021046961
H	-1.57985379158960	-1.98987381528979	10.39142086573104
H	-3.18476132920738	-1.91086251088515	9.68875746787835
H	-1.44362233413241	-10.53051329319083	-1.86615487215528
H	-2.46082361193099	-9.55375875592456	-2.90755097723567

C	1.76343688815709	2.57007681632731	6.73253488829440
C	3.63059339691786	4.01503938139377	6.30104273065480
H	1.07516440061154	-3.28515443592207	-5.52821285287085
H	2.58279389800815	-4.16465802243248	-5.75607889549966
H	4.40755615871033	-1.12081338688227	-6.68374457670643
H	4.60023039772286	-2.85890101505883	-6.45307262868089
H	0.92531375690555	-6.27837919267235	2.24572845792253
H	2.12991454030513	-7.03829528221104	3.28401128933237
H	4.89935271314795	-6.09392510683025	1.06774571340684
H	4.52861088721858	-6.92014855070048	2.58194734232515
H	3.26423167116054	7.19867437527560	-3.90663984243592
H	3.83490449930251	5.58460824946601	-4.33205805949190
H	-0.19231918279604	6.03814191956201	-3.44374226573011
H	0.83195016590849	7.47042601340956	-3.37873655258929
H	0.91226829552005	2.08362484646981	6.26013706699114
C	1.29778867460017	3.43987791675052	7.88464795799729
H	4.12819452789240	4.58059056758221	5.51230239494517
C	3.23980404301356	4.93784238028973	7.44041213895352
C	1.59720750099742	-3.61384109477010	-7.58914589060378
C	3.61001382033325	-2.31935078794884	-8.28503435810407
C	1.65762909424990	-8.16915447831538	1.50898944636290
C	4.05235165169039	-8.06013416004613	0.81402101133649
C	2.74841305950241	6.58056364499820	-5.90615654581351
C	0.32382090378302	6.84071889604654	-5.37855239150165
H	2.41419851127155	1.76662805023717	7.12254904521995
H	4.36110211788442	3.26999447723960	6.66889286302361
C	1.52103386626185	7.46131486444926	-6.08107631637676
C	2.82928517901808	-3.60953739255626	-8.47960935068511
C	2.96734386025992	-8.93640085238705	1.42029513178686
C	2.46608304832688	4.18438500616187	8.51078021813093
H	0.79368762595320	2.81572699734852	8.62464182879054
H	0.56110372369277	4.15688578801884	7.51432019693832
H	2.62262622343325	5.74658444566368	7.04225693583788
H	4.13813714493047	5.39509022936586	7.85883627043122
H	0.89477670163030	-2.84901685302113	-7.92892607643491
H	1.08023247677121	-4.57333176874155	-7.64671904125998
H	4.54886309877432	-2.34437608169482	-8.84109834280287
H	3.02981883238489	-1.47776053371149	-8.67056275238058
H	1.27733450281877	-7.96612991483364	0.50565017687762
H	0.89641840605964	-8.75415819944556	2.02572124218239
H	5.01750595064272	-8.56694968620238	0.83139816740657
H	3.81724989672323	-7.85450821072012	-0.23243580356770
H	2.60767887650059	5.64102369558315	-6.44475141835835
H	3.63404952808570	7.05909475813607	-6.32505543671469
H	-0.54087716788329	7.50338923656604	-5.42049303847418
H	0.04111682211323	5.91302387057499	-5.88054094769623
H	1.71979002138832	8.44769363507044	-5.65273869254438
H	1.30831093204026	7.61435043908237	-7.13941548654283
H	3.46655949704219	-4.45818520766159	-8.21352323201886
H	2.55235251940459	-3.73987221994837	-9.52703654915398
H	3.27184976065179	-9.24764319900326	2.42343816786684
H	2.83979605306475	-9.84636944102608	0.83345761488914
H	3.13284787718618	3.46485821978287	8.99530993774781
H	2.12013654929780	4.86612348080030	9.28942499493516

7

C	0.13496646183160	1.37300741178482	-2.91799816717040
C	-0.96405935260351	1.43256975556405	-3.77450613358643
C	-1.54968017641207	0.27681288339359	-4.30203150718798
C	-1.00662066834325	-0.96884002459314	-3.96936087485317
C	0.11964562245527	-1.06115041755201	-3.14036705584417
C	0.63202101555191	0.11628824246719	-2.61071990935337
O	-1.47468266261262	2.66003842761429	-4.10414320005147
O	-1.61960337338996	-2.06535286788373	-4.50767908818732
H	1.48778847583434	0.04539282578414	-1.95547370904133
C	0.77543768346258	-2.39572805926988	-2.83737360548934
C	0.20602239332367	-2.98236905019095	-1.56328624387281
C	-0.86099999970267	-3.87819199461160	-1.62223739194057
C	-1.43084486719577	-4.42198111849762	-0.46615829740242
C	-0.90089269657855	-4.06874398609909	0.77936195459732
C	0.19659325879039	-3.20176282466774	0.87096563059648
C	0.69069288892054	-2.65565194799319	-0.30663200702966
O	-1.35617742883328	-4.23067132234662	-2.84989920058410
H	1.52111902935240	-1.96844947494588	-0.23649834642069
C	0.84502084631900	-2.87984199887807	2.20451260987029
C	0.24678160642699	-1.61835921650368	2.78918426012346
C	-0.82294182492056	-1.70010070803932	3.68008822132743
C	-1.41603735342976	-0.55641606118407	4.22529764510452
C	-0.90928202965265	0.70006198848585	3.87647003348289
C	0.18827094311307	0.81503330332304	3.01232536991298
C	0.70760287180277	-0.35177340221559	2.46656605698231
O	-1.29656001039576	-2.93775848574424	4.02689827480891
O	-1.52652138106193	1.78393220501560	4.43514039519202
H	1.54091334244610	-0.26418424936256	1.78499490886699
C	0.80863553917125	2.16206196171858	2.69034881935155
C	0.19150320853264	2.73727453828740	1.43342093028201
C	-0.89181178155668	3.61086257738201	1.52391798177676
C	-1.50325189613563	4.14589589772068	0.38515291791890
C	-1.00260446506691	3.80315995524897	-0.87542392780666
C	0.10821128140935	2.95757637742476	-0.99945315758461
C	0.64700836346694	2.42157064144456	0.16314021898969
O	-1.35982197749823	3.95064547140020	2.76562649170711
O	-1.63907133709811	4.34903523132347	-1.95472478008668
H	1.49099709475649	1.75409587865399	0.06840537932641
C	0.72401136570306	2.64717704897019	-2.35140913367800
C	2.30774761835642	-2.30883967325836	-2.87058855608633
C	2.37764553876424	-2.87804966707276	2.11582707820742
H	0.59684180179320	-3.69453016286393	2.88420310985678
C	2.34268709363523	2.10441083456134	2.67980781089561
H	0.55157846238459	2.83493367567160	3.50798614751496
C	2.25836305289270	2.67741763613511	-2.30699926943869
H	0.44012098197440	3.45561896721366	-3.02463723671098
H	2.57848048675968	-1.59057193097148	-3.64545161404162
H	2.69866428033619	-1.89780386514552	-1.93863030624183
C	3.03138455835064	-3.61871249177654	-3.18115171980852
H	2.66494658851026	-3.64508815940475	1.39558653187582
H	2.74695909591905	-1.93679528359564	1.70613786377734
C	3.10907317347810	-3.17509124252278	3.42443028976336
H	2.64850908507660	1.39029908941478	3.44546666113871
H	2.71483801526597	1.70192458147215	1.73654224272757
C	3.04954499929255	3.42723420132589	2.97212392703619
H	2.54934679227001	3.45209596035064	-1.59642591716397
H	2.65905883552494	1.74504846994310	-1.90632064120164
C	2.94643951648846	2.98721595029828	-3.63581773267566
H	0.50797569981603	-3.07399374300854	-3.64722784978681
H	-1.91258911781194	-5.07896014145463	-2.68834826500297
H	-1.85930866919570	-2.78918962582372	4.87348313901295
H	-1.40835254797600	2.58188661240688	3.87618022875963
H	-1.93990505333409	4.78641442763228	2.62169049721495
H	-1.52397650185949	3.78518316457852	-2.74980698692987
H	-2.06827412321408	2.49913298159509	-4.92735333006162
O	-1.49535430238584	-4.62567368290276	1.87690917703538
H	-1.46666613292949	-2.86079626825584	-3.95358059504315
H	-1.36535772044919	-4.06156982922289	2.66938396477400
C	2.79270803671164	-4.72031549520619	-2.15811964638471
C	4.52410729445779	-3.35437129693628	-3.33712466231757
C	4.44485258457469	3.15006931762202	-3.41060381226795
C	2.68398522915671	1.96028477502497	-4.72837979323863
C	4.55028043994042	3.19040140961466	3.09105864153551
C	2.76509842467076	4.52343787459838	1.95480776615148
C	4.60477937446192	-3.29636119659449	3.15895619227696
C	2.84720547679411	-2.16030571352815	4.52847024138370
H	2.65327824230656	-3.97415240463910	-4.14640665039034
H	2.75304869381801	-4.14946029590523	3.77770001119766
H	2.68905858044464	3.77640718039975	3.94633490930976
H	2.55543176197944	3.95003325767334	-3.98390751753831
H	1.74371576348504	-4.99728755418885	-2.09114566729277
H	3.10973152216327	-4.40398556286275	-1.16359521657535
H	3.36025029726812	-5.61373410260563	-2.42099571639075
H	4.95459271441658	-3.00494190331683	-2.39679788985942
H	4.71872012066480	-2.59426456695755	-4.09409841266188
H	5.05705828063735	-4.26022956404534	-3.62621261796079
H	4.88814481182833	2.21270531401140	-3.06929204558482
H	4.65579177285979	3.91033816935551	-2.65816085530463
H	4.95316800138090	3.43833507113934	-4.33077230855081
H	1.62784414495050	1.88314233909877	-4.97358294108653
H	3.01988606568950	0.96928818310216	-4.42036790864114
H	3.22224760410818	2.22667494627034	-5.63874828859625
H	4.96407884751541	2.84891762344256	2.14037067996557

C	0.072540000	1.511215000	-2.709361000
C	-1.012290000	1.647561000	-3.575949000
C	-1.611141000	0.539340000	-4.184036000
C	-1.086993000	-0.732469000	-3.932821000
C	0.029139000	-0.900820000	-3.102091000
C	0.542647000	0.229048000	-2.478167000
O	-1.493858000	2.901491000	-3.834909000
O	-1.712094000	-1.781791000	-4.549010000
H	1.383184000	0.102703000	-1.811946000
C	0.665922000	-2.264680000	-2.902854000
C	0.117890000	-2.947488000	-1.667435000
C	-0.929585000	-3.863187000	-1.760807000
C	-1.462799000	-4.499810000	-0.640292000
C	-0.925507000	-4.192813000	0.620153000
C	0.146771000	-3.300635000	0.751670000
C	0.620445000	-2.689122000	-0.401321000
O	-1.413119000	-4.115479000	-3.031212000
H	1.439545000	-1.989524000	-0.310739000
C	0.793254000	-3.025173000	2.097789000
C	0.161479000	-1.810850000	2.746897000
C	-0.923493000	-1.968410000	3.610004000
C	-1.552101000	-0.872810000	4.211322000
C	-1.060461000	0.410471000	3.954790000
C	0.056062000	0.601166000	3.130345000
C	0.607518000	-0.518373000	2.519732000
O	-1.375233000	-3.232123000	3.876511000
O	-1.714802000	1.446111000	4.562416000
H	1.457694000	-0.373625000	1.870083000
C	0.664794000	1.977532000	2.929364000
C	0.074756000	2.646559000	1.704563000
C	-0.980170000	3.551471000	1.817076000
C	-1.544253000	4.179318000	0.706877000
C	-1.029704000	3.875030000	-0.563962000
C	0.051481000	2.998211000	-0.712925000
C	0.550627000	2.389234000	0.429674000
O	-1.430650000	3.809556000	3.098784000
O	-1.619725000	4.498467000	-1.621737000
H	1.377393000	1.703547000	0.318521000
C	0.685907000	2.736611000	-2.066287000
C	2.211128000	-2.228656000	-2.957412000
C	2.324337000	-2.978772000	2.002460000
H	0.569613000	-3.878586000	2.737329000
C	2.199970000	1.936213000	2.949097000
H	0.391221000	2.578651000	3.795423000
C	2.218851000	2.718460000	-1.986848000
H	0.440376000	3.585875000	-2.703294000
H	2.560822000	-1.199021000	-2.963904000
H	2.634783000	-2.686781000	-2.063647000
C	2.786334000	-2.941763000	-4.178821000
H	2.626646000	-3.709472000	1.251084000
H	2.666878000	-2.013365000	1.627136000
C	3.071688000	-3.309972000	3.294211000
H	2.492952000	1.185858000	3.684330000
H	2.596761000	1.584769000	1.995632000
C	2.891420000	3.247046000	3.323348000
H	2.514465000	3.460653000	-1.244247000
H	2.582482000	1.762691000	-1.606649000
C	2.950125000	3.050303000	-3.287430000
H	0.363063000	-2.871217000	-3.751944000
H	-2.197991000	-4.677317000	-2.979367000
H	-1.963494000	-3.143394000	4.717284000
H	-1.547257000	2.283826000	4.088439000
H	-2.227845000	4.355150000	3.063802000
H	-1.516832000	3.964089000	-2.439062000
H	-2.074909000	2.803895000	-4.678946000
O	-1.484027000	-4.831221000	1.685648000
H	-1.549155000	-2.610535000	-4.058392000
H	-1.386033000	-4.292998000	2.501865000
C	2.562417000	-4.446570000	-4.108627000
C	4.267185000	-2.624120000	-4.333308000
C	4.445880000	3.161699000	-3.017026000
C	2.690405000	2.067061000	-4.420318000
C	4.385620000	3.002166000	3.498033000
C	2.655094000	4.381748000	2.336370000
C	4.567951000	-3.385020000	3.014878000
C	2.795110000	-2.347472000	4.440640000
H	2.264431000	-2.557818000	-5.062562000
H	2.741288000	-4.305981000	3.609675000
H	2.489319000	3.558802000	4.293886000
H	2.595988000	4.034659000	-3.613556000
H	1.509026000	-4.702178000	-4.001014000
H	3.093015000	-4.869749000	-3.253774000
H	2.931050000	-4.941165000	-5.007217000
H	4.827774000	-2.962712000	-3.460023000
H	4.433267000	-1.551937000	-4.439369000
H	4.689941000	-3.118083000	-5.208143000
H	4.852344000	2.201267000	-2.694379000
H	4.657954000	3.891493000	-2.235398000
H	4.987134000	3.463212000	-3.913781000
H	1.641528000	2.034070000	-4.703604000
H	2.981972000	1.056009000	-4.133340000
H	3.268578000	2.342869000	-5.302882000
H	4.837764000	2.687215000	2.555696000

C	0.12649849868451	-2.20937956684892	-2.14449742981377
C	1.29165804223071	-2.43306822252493	-2.89450486647116
C	1.77148162863086	-1.47036126394939	-3.78995162214336
C	1.08684492963581	-0.25370194616035	-3.91144046989268
C	-0.07771552637023	-0.00106460664131	-3.17708103103352
C	-0.50599794311413	-0.98597498917627	-2.29918072213009
O	1.99681333455203	-3.60080797282463	-2.81612448708173
O	1.61405008493130	0.65214746465458	-4.78588326233589
H	-1.40521365331487	-0.79932136149077	-1.73217414186759
C	-0.85821751305443	1.28634875464283	-3.34446236547909
C	-0.37571476743278	2.35039433608758	-2.38137423274100
C	0.53607801339773	3.31453298899789	-2.81055622735061
C	1.01257986866319	4.31696335626999	-1.95159194293874
C	0.53092872826646	4.34018511350340	-0.64284203590670
C	-0.37502811016561	3.38790213106423	-0.17037776380489
C	-0.78981839774389	2.40431427464064	-1.05772764979347
O	0.97518799099861	3.26979985517482	-4.10300642514958
H	-1.47182795086251	1.64671380719330	-0.70003230176602
C	-0.86768545013282	3.43404614687904	1.26308866197040
C	-0.10014569278127	2.47677769053112	2.15592574510577
C	1.02760105846864	2.89671755381817	2.87224490378875
C	1.69892515129624	2.03463475408292	3.74789569005437
C	1.24602034711804	0.71760152434702	3.87641717440300
C	0.11749880861744	0.26431407877375	3.17951189591304
C	-0.50708048115968	1.16016017100741	2.32384478786834
O	1.52708984676204	4.16418874585154	2.76932910067287
O	1.93977204257348	-0.08882155650190	4.73554578234924
H	-1.38743956661067	0.82880818682457	1.79312150821100
C	-0.42024758353987	-1.13867968825378	3.38697741480049
C	0.16798910153678	-2.12086461839538	2.39336341615467
C	1.23579838810933	-2.94940265493542	2.74752448219443
C	1.79109055672621	-3.86305780880865	1.85168214250160
C	1.23682428104857	-3.94232065549468	0.56389268336520
C	0.16713208511238	-3.13878557799037	0.17527140022333
C	-0.32463093044478	-2.23160907985799	1.10177529678705
O	1.71776090123272	-2.80850072174633	4.03573988910544
O	1.75119590713070	-4.82669222330823	-0.34070042823688
H	-1.14277488328194	-1.58991385086275	0.80766827073812
C	-0.42389407728935	-3.26905957916004	-1.21123770289838
C	-2.37714204681045	1.09883941639127	-3.28622101461141
C	-2.38820393017519	3.26846467374867	1.35463858804367
H	-0.67124167774612	4.43887334327625	1.62485217682609
C	-1.95020923747800	-1.21021950454883	3.42222487585637
H	-0.10302692318895	-1.46397564812009	4.37384821855112
C	-1.95380970076996	-3.32289978842047	-1.19582615437478
H	-0.10207384642782	-4.23050948223204	-1.60309171486936
C	-2.95206389635645	0.10381726719296	-4.29191509254969
H	-2.69061938676672	0.80102569467019	-2.28547491993726
H	-2.83175468299481	2.07802952662274	-3.45877339168868
H	-2.83967120990875	4.00553368272220	0.68544884745294
H	-2.69227941835828	2.29492185849237	0.97053672829436
C	-2.97523844953208	3.44255096489574	2.75298403390462
C	-2.61785397297370	-0.33841905378450	4.48246236332924
H	-2.36955477722589	-0.95307729257992	2.44993369304134
H	-2.22450780316037	-2.25423503781996	3.59562898445569
C	-2.55487160737539	-4.42563204817551	-0.32675650746222
H	-2.36978537040484	-2.37166701984138	-0.86540475725561
H	-2.29173956071439	-3.45470131585358	-2.22695708673347
H	-0.64863477362358	1.66092063726697	-4.34292384441871
C	2.88250374496791	2.53290934921050	4.51743987012329
C	2.97745877579989	-4.72302705126161	2.19061702325826
H	1.37913670518740	4.18879770193199	-4.29316431821663
H	1.24795170718136	4.57277004184985	1.92936133226205
H	1.78452134722865	-1.02742715499939	4.52322029914617
H	2.54029492508113	-3.29916385970794	4.15005271725997
H	2.29426849251930	-5.50016205746218	0.20599668842164
H	1.83519192766322	-4.05993329572664	-1.96813356054503
O	0.93499401904557	5.30244271071004	0.26317790452921
C	3.00811308624449	-1.70441768081027	-4.60129867540103
H	1.31241053014574	1.55883539794278	-4.57972650641203
C	2.05330003698035	5.27571308307666	-2.45914930784630
H	1.64119582906188	5.84530416828701	-0.10722478975285
N	2.71844958745139	-2.05334781424454	-5.99727888261904
N	1.76986835909109	5.70551525161577	-3.83399454103321
N	2.50323235494505	3.33248548383971	5.68924363397287
N	2.85075468943095	-6.06923861647883	1.61820479949340
H	3.51962477290575	1.69027473652280	4.81133341686314
H	3.47569192339918	3.18201929979908	3.87575871037952
H	3.88254984886611	-4.28205241693499	1.76833954876054
H	3.14422035377621	-4.80127008392272	3.27191104175963
H	3.59398022015842	-0.78687251882974	-4.62507963643635
H	3.62400272537966	-2.47736289655927	-4.12696793609420
H	2.16557753571489	6.15759122846955	-1.81611829507249
H	3.02662610318885	4.78140172770625	-2.47584970145249
C	-4.47745362988934	3.19797252717828	2.71545338257908
C	-2.66019111981576	4.80365378647820	3.35746137282027
C	-4.13017206064353	-0.49667461412618	4.40342175582532
C	-2.11187580216900	-0.62838638594603	5.88808110232327
C	-4.07519454126632	-4.36028132859079	-0.39141529766745
C	-2.05497960175207	-5.81325250932962	-0.70437774174852
C	-4.45977031594743	-0.00616025187013	-4.10624258724588
C	-2.60460243005725	0.46143731221721	-5.73040254726638
H	-2.51923043037311	-0.87636395005807	-4.07875914498753

H	4.77734410617415	2.43385537435309	3.84256442718311
H	5.07347665274659	4.10581018146620	3.36760060620544
H	1.71027747421563	4.78335308614750	1.91653382572254
H	3.05927980457723	4.21053827856817	0.95218182539782
H	3.32515598073821	5.42635009967008	2.20072808251974
H	5.01373082594683	-2.34559785306055	2.81192041841230
H	4.81605471403233	-4.04651014833824	2.39651633320422
H	5.14464723605859	-3.57599022638300	4.06368499010921
H	1.79759636204443	-2.12147254487338	4.80867449223189
H	3.13664320554411	-1.15723109486853	4.21271608587365
H	3.42406339888074	-2.40950746201049	5.41982431232920
C	-2.77523413566943	0.38573193730241	-5.16346033302988
C	-2.62344753203544	-5.32804584138524	-0.57778413078502
C	-2.60775388336212	-0.69240635840364	5.12930911524640
C	-2.70821460850024	5.03066234526450	0.53020560253101
N	-2.46618822969340	-1.84659192235853	6.03460144328061
C	-1.43870449233126	-1.62163858545499	7.05576969462680
N	-2.50000037400393	-6.23853889447811	-1.72998567366985
C	-1.46802961959594	-7.25780887759121	-1.51681957327158
N	-2.69807895896296	1.54865757410090	-6.06473533272949
C	-1.70544994375775	1.35774032813744	-7.12687479040535
N	-2.57316298640824	5.93634895749064	1.68469253580610
C	-1.56451922761977	6.97511637751138	1.45475287553674
H	-3.65529709080363	0.53607502682723	-4.53366068558639
H	-2.93983332645930	-0.53391249854927	-5.72645347220256
H	-3.52395247345851	-4.73247099379941	-0.74505004100189
H	-2.77759799124946	-5.88666497476325	0.34627852585134
H	-3.50465002298196	-0.87165160111026	4.53179166717171
H	-2.77784180102178	0.22625276772273	5.69217298176698
H	-3.59447195501572	4.41897978257863	0.71456965941123
H	-2.89308025727293	5.59200064827203	-0.38657593132430
C	-3.84491936514050	6.54898363074354	2.06776090751059
C	-3.73053408989140	-2.21816250058793	6.66942028818911
C	-3.76967323370900	-6.87607473668135	-2.07782244922439
C	-3.99480174039047	1.89319108599308	-6.64716910489204
C	-1.42350983699011	7.83394003991213	2.68897163027708
O	-2.66493522612134	8.43045219193856	3.05703767980965
C	-3.64624084371665	7.42324834959212	3.28535262925309
C	-1.29318715726798	-2.85390109926774	7.91663672344866
O	-2.52747268734240	-3.21046056361774	8.53452331988166
C	-3.52679275160828	-3.43447175312490	7.54422198620696
C	-1.34210372859924	-8.12522879908510	-2.74656585947222
O	-2.58157158807122	-8.74567428309726	-3.07974230579018
C	-3.58504188013557	-7.75717481886944	-3.29263610820782
C	-1.62357206897795	2.60070615761716	-7.98053256547759
C	-3.85521474745637	3.12135846983027	-7.51803734379141
H	-1.70506683788857	-0.75812960921954	7.67848056690160
H	-0.49349411062290	-1.40163438492339	6.56251123076336
H	-1.71944714768444	-7.87585187431415	-0.64552393682226
H	-0.51967059303920	-6.76268798584834	-1.31540792132243
H	-1.97440568688510	0.49329206735324	-7.74710929026318
H	-0.73675002836318	1.15707742887639	-6.67240271934476
H	-1.84790215260626	7.59635563234369	0.59560623126538
H	-0.61309207071981	6.49834132053153	1.22551563703151
H	-4.56127029965451	5.76085833559160	2.29709997270645
H	-4.24961140401150	7.14635939977044	1.24038142569300
H	-4.46143473460753	-2.44445911776166	5.89386570113077
H	-4.11915146032146	-1.38584539162869	7.27060817790478
H	-4.50458877530615	-6.10214021753014	-2.29660764052027
H	-4.14464448915285	-7.47297660957581	-1.23621564174731
H	-4.70058705809684	2.09494089306995	-5.84214200095610
H	-4.38568337455478	1.05624820092885	-7.24038696662907
H	-0.72652406861682	8.64827237624855	2.50558968668138
H	-1.04256933862646	7.22807921482344	3.51971991097923
H	-4.57133185452530	7.93679527307415	3.53660626140252
H	-3.34744835734431	6.80223967474620	4.13824593255334
H	-0.92818371958054	-3.68976385620386	7.30799103700175
H	-0.58179536226850	-2.67274841897464	8.71892608878022
H	-4.44524974622229	-3.67704344076316	8.07361418929855
H	-3.24474958292479	-4.29161129015303	6.92122207249227
H	-0.62741720576955	-8.92618408781015	-2.57255357900328
H	-0.99086768936994	-7.52100243244132	-3.59149383498486
H	-4.50698472257925	-8.28841281930243	-3.51731471226043
H	-3.31762409909283	-7.13859070721425	-4.15764198354663
H	-0.93902082984559	2.44470302842468	-8.81097360630345
H	-1.25640316111179	3.43991529700467	-7.37783874095298
H	-4.79901181532103	3.34462184101968	-8.01003325018849
H	-3.57143591521904	3.98035220428413	-6.89841061940556
O	-2.88906581759063	2.93121378427267	-8.54759414607048

H	4.579816000	2.223665000	4.236071000
H	4.898120000	3.907814000	3.822227000
H	1.604279000	4.646419000	2.255926000
H	2.999240000	4.106947000	1.338361000
H	3.202600000	5.273354000	2.643703000
H	4.951624000	-2.412621000	2.700415000
H	4.791860000	-4.101676000	2.224457000
H	5.120436000	-3.684232000	3.905539000
H	1.747117000	-2.343513000	4.729376000
H	3.060297000	-1.326315000	4.164451000
H	3.384144000	-2.620498000	5.316841000
C	-2.825337000	0.728543000	-5.047704000
C	-2.575374000	-5.499160000	-0.731215000
C	-2.762672000	-1.086984000	5.074261000
C	-2.661543000	5.171268000	0.817464000
N	-2.620254000	-2.294161000	5.907133000
C	-1.630281000	-2.113257000	6.973841000
N	-2.109774000	-6.881496000	-0.673949000
C	-3.221732000	-7.811396000	-0.538948000
N	-2.709314000	1.941545000	-5.875960000
C	-1.717087000	1.784520000	-6.944455000
N	-2.204452000	6.555428000	0.737531000
C	-3.322643000	7.479075000	0.611785000
H	-3.706483000	0.863602000	-4.416053000
H	-3.008331000	-0.150724000	-5.666606000
H	-3.195187000	-5.334632000	-1.632929000
H	-3.247134000	-5.349689000	0.110933000
H	-3.640446000	-1.243836000	4.443015000
H	-2.965600000	-0.210076000	5.690356000
H	-3.257848000	5.011046000	1.735430000
H	-3.352776000	5.011478000	-0.006891000
C	-1.369615000	6.941068000	1.864930000
C	-3.894114000	-2.737069000	6.474122000
C	-1.293151000	-7.251897000	-1.819777000
C	-3.992580000	2.359369000	-6.440862000
C	-2.813723000	8.895678000	0.470183000
O	-1.995317000	9.272495000	1.575454000
C	-0.900257000	8.368429000	1.703693000
C	-1.480660000	-3.395356000	7.757495000
O	-2.724412000	-3.819502000	8.309935000
C	-3.686184000	-4.002457000	7.275097000
C	-2.704815000	-9.227135000	-0.420117000
O	-1.903287000	-9.589136000	-1.542578000
C	-0.814718000	-8.678632000	-1.680321000
C	-1.594413000	3.071759000	-7.724216000
C	-3.811467000	3.631707000	-7.237423000
H	-1.937703000	-1.298695000	7.641693000
H	-0.676251000	-1.839001000	6.526765000
H	-3.909326000	-7.747887000	-1.399915000
H	-3.791169000	-7.559146000	0.356123000
H	-2.009472000	0.966265000	-7.614517000
H	-0.757121000	1.527795000	-6.499648000
H	-3.996175000	7.421363000	1.484181000
H	-3.905271000	7.215535000	-0.271480000
H	-0.502677000	6.284201000	1.915043000
H	-1.917821000	6.846193000	2.819185000
H	-4.593136000	-2.930374000	5.661229000
H	-4.323989000	-1.955425000	7.113812000
H	-0.430360000	-6.590085000	-1.878719000
H	-1.858562000	-7.150470000	-2.763293000
H	-4.695053000	2.535171000	-5.626964000
H	-4.406332000	1.571161000	-7.083132000
H	-3.643872000	9.598345000	0.442388000
H	-2.242459000	8.989100000	-0.460883000
H	-0.336103000	8.683492000	2.578886000
H	-0.252436000	8.443944000	0.822349000
H	-1.074679000	-4.181082000	7.109272000
H	-0.799901000	-3.249804000	8.592908000
H	-4.614147000	-4.300498000	7.757419000
H	-3.363292000	-4.810661000	6.608135000
H	-3.531550000	-9.933549000	-0.385932000
H	-2.117929000	-9.327727000	0.500430000
H	-0.263643000	-8.982704000	-2.567632000
H	-0.152350000	-8.759128000	-0.810326000
H	-0.911910000	2.942484000	-8.560882000
H	-1.203537000	3.863525000	-7.074058000
H	-4.745799000	3.912575000	-7.717737000
H	-3.504186000	4.443767000	-6.567808000
O	-2.847197000	3.472260000	-8.273880000

H	-2.52782406117913	2.68258169247151	3.39669760272317
H	-2.38125543068448	0.70238651089206	4.25367361437390
H	-2.25663981190775	-4.23338803009914	0.70738250857670
H	-4.97701631706214	3.94364639877040	2.09408575470241
H	-4.70798781403227	2.21518406285580	2.30403029470293
H	-4.91283142732557	3.25243454268001	3.71312685164887
H	-1.59253985742108	4.94575392298631	3.51623963408050
H	-3.01277953442222	5.60896273545326	2.70981778961624
H	-3.14948197849166	4.91863704349644	4.32452731909177
H	-4.50110573399929	-0.26358090100663	3.40513755953698
H	-4.42865531068112	-1.52028388981639	4.63770336667262
H	-4.63322306145396	0.16486464730511	5.10845982095743
H	-2.24537005275953	-1.68193774847083	6.14238832582545
H	-1.05583927188712	-0.38839033876977	6.00096690288319
H	-2.65856242851077	-0.03892919098538	6.62395673734717
H	-4.44314876483144	-3.37583954297331	-0.10071330962983
H	-4.42964845803568	-4.56069743256413	-1.40418518026744
H	-4.53102553368659	-5.09688864457450	0.27019575454632
H	-2.25073235796238	-6.02422502491513	-1.75753698896109
H	-0.98516869896078	-5.92301413861049	-0.53620009417860
H	-2.55863498985190	-6.57924975752841	-0.11461083727535
H	-4.71299177053283	-0.29345498894454	-3.08518252111739
H	-4.94958617791214	0.94693329881137	-4.31449263175412
H	-4.88624464000309	-0.75099787044317	-4.77810854739526
H	-2.94697800102871	1.46922965532430	-5.97396813265325
H	-1.53260186405902	0.41832557998534	-5.91504306604963
H	-3.08222584620402	-0.22809551075389	-6.42635327776410
C	2.03690572783650	-3.33355350987679	-6.12636860675018
C	3.93526258899595	-2.06975642023742	-6.79482382947626
C	1.79895587510962	-6.85610877228675	2.27061378772819
C	4.11610274746570	-6.80545836686440	1.63186532311431
C	2.91934963164004	6.35872426519514	-4.46226570058873
C	0.58934979076377	6.57219150487286	-3.91423009515786
C	1.80185904445463	2.55094071286223	6.69902310588473
C	3.67115817220403	3.96090164118139	6.28909462672851
H	1.10240619940041	-3.30223285055967	-5.56820723585806
H	2.64238309962843	-4.15585771405756	-5.71080468760007
H	4.41341910050735	-1.09103651697724	-6.73712590923003
H	4.65764976615055	-2.81481583956784	-6.41799215437941
H	0.85587497054363	-6.31813199053442	2.19421065958091
H	2.03058845021746	-6.98895532347193	3.33499817780240
H	4.86255684644824	-6.23202586667467	1.08379828148372
H	4.47377700798874	-6.93645925982156	2.66128426795705
H	3.20297996170167	7.25956161990546	-3.90277720737863
H	3.76459048145278	5.67196760481435	-4.45072877826445
H	-0.26597524587791	6.04504528229840	-3.49547313422077
H	0.74875450066596	7.48359047546727	-3.32393749581871
H	0.90126065120919	2.12215027194426	6.26220614462975
C	1.41808019548983	3.43306526822156	7.86365124345806
H	4.16250697462692	4.58761302167868	5.54353338657171
C	3.25890660613475	4.81772308683140	7.46434783921732
C	1.73929227948242	-3.61676267046743	-7.58011031040852
C	3.61015149250059	-2.37940578066417	-8.23876645104790
C	1.67591073467232	-8.20234850929941	1.59676610771796
C	3.92844214083717	-8.15498395804528	0.97663368720762
C	2.58189554518538	6.72637737945877	-5.88951927816472
C	0.31565576581870	6.92949932736523	-5.35596299208156
H	2.42293517841953	1.71662387672564	7.06381966641797
H	4.40600673680532	3.20933204339084	6.62595486405972
O	1.43679105400224	7.57079308805969	-5.95797392574370
O	2.92739807605914	-3.62409474925392	-8.37096361793443
O	2.90994617882886	-8.91446646433780	1.62056791770460
O	2.55799060614322	4.06114802075814	8.44899756992835
H	0.95123398525064	2.84321757048765	8.65000263562720
H	0.70633812406279	4.19803357925443	7.53002358290074
H	1.04360120715896	-2.86253139707082	-7.96754203255666
H	1.28603742677922	-4.59965495955388	-7.69236147395121
H	4.52254681392110	-2.46053330773719	-8.82697491836172
H	2.99386765585317	-1.57426697513867	-8.65649229074973
H	1.34414730927684	-8.06914733583861	0.56043422757852
H	0.94702943038011	-8.81846608367421	2.11761645924409
H	2.40710660576886	5.81562895257669	-6.47413139644050
H	3.40521070519234	7.27511664680119	-6.34051966444798
H	-0.51690668698427	7.62577650866251	-5.42024577612532
H	0.05918082895454	6.02359867451189	-5.91742226140578
H	2.62601922587687	5.64131351155223	7.11223372371190
H	4.13366950079767	5.23682507469016	7.95767064176628
H	3.67663664772566	-8.01858963917886	-0.08154400457449
H	4.84511301542534	-8.73584501224629	1.04377388954984

8

C	0.18619282807960	1.40660311948242	-2.88086530570537
C	-0.93204798263666	1.42816162979220	-3.71488045274799
C	-1.51304736579500	0.24957022505854	-4.19575778292106
C	-0.95284144905648	-0.97906431516080	-3.82950005388249
C	0.18783425244428	-1.03395515401490	-3.01701256032307
C	0.70058537722693	0.16583096748232	-2.53990238534281
O	-1.46565132525883	2.63835482380729	-4.06800963221063
O	-1.56744939484759	-2.09766997487496	-4.31847244054766
H	1.56862518744923	0.12568125491927	-1.89840346257299
C	0.85157289490071	-2.35414513801140	-2.67079124287726
C	0.28409473940273	-2.90142170662880	-1.37831304791602
C	-0.76961787194357	-3.81432741349417	-1.40873787884669
C	-1.34014401317053	-4.31950908052595	-0.23553312123783
C	-0.82343799338184	-3.90977032074748	0.99786961363575
C	0.26394031149468	-3.02752789468151	1.06197567266101
C	0.75815393193844	-2.52028953166280	-0.13300010110611
O	-1.25094500147631	-4.22275483925824	-2.62404987207661
H	1.57823148718472	-1.81841542811435	-0.08682061524732
C	0.90095770022108	-2.65028069721839	2.38642774477164
C	0.30024899390821	-1.36575086107923	2.91488119248962
C	-0.78250613632994	-1.41123658859135	3.79275194366822
C	-1.37648319124919	-0.24571233422218	4.28907475175860
C	-0.86038284991562	0.99510232470660	3.90064500482250
C	0.24907004675768	1.07424207266635	3.04759658640625
C	0.77117132872487	-0.11383853046271	2.55243192589888
O	-1.26723657386765	-2.63239814971614	4.17836458884557
O	-1.48095798754673	2.10086890406797	4.41096312983946
H	1.61292985582249	-0.05524849255137	1.87818461499395
C	0.87448322306678	2.40745371525129	2.68229588570611
C	0.25931846151174	2.94417907333458	1.40778224292634
C	-0.80877357054055	3.83865887993354	1.47041529773468
C	-1.41603090128521	4.34106008817009	0.31461686875154
C	-0.92747918378889	3.94204089794445	-0.93378546171025
C	0.16870264195982	3.07390796913990	-1.03079063345156
C	0.70416940699928	2.57279394298117	0.14889760684187
O	-1.26549081110113	4.23222832172866	2.69992913014102
O	-1.55856302097155	4.45611877682675	-2.03220222074356
H	1.53560872957873	1.88650581860651	0.07784464719617
C	0.77438035556439	2.70543282454061	-2.37276528290301
C	2.38347574979037	-2.26341730428378	-2.70404193614359
C	2.43456582607846	-2.65552207680496	2.31190054423681
H	0.64443869546905	-3.43620363870467	3.09618670837270
C	2.40840794585225	2.34743459518409	2.67140744551277
H	0.61903842282389	3.10666036600849	3.47803071223286
C	2.30910352098316	2.74381956997776	-2.34076383918261
H	0.48158914457927	3.48306350441386	-3.07767951989346
H	2.65507903258028	-1.56235436048876	-3.49416950253099
H	2.77145526736275	-1.83207405527430	-1.78005815093521
C	3.10922675055639	-3.57906872555537	-2.98375239592442
H	2.72693476586249	-3.44387833688014	1.61717870517537
H	2.81094716781804	-1.72742510166859	1.87950480450994
C	3.15016323969255	-2.91704334585880	3.63659801258344
H	2.71608391279389	1.65102481204280	3.45246384300474
H	2.77850041169342	1.92487742333210	1.73617962047774
C	3.11466473205927	3.67710353134234	2.93235697225159
H	2.60213358308284	3.54491967136231	-1.66096550510379
H	2.71705576773504	1.82828625015035	-1.90987491533246
C	2.98447853514999	3.00871305278975	-3.68557534498844
H	0.58660665270045	-3.05932836300220	-3.45823089930349
H	-1.79815994769032	-5.07432275402190	-2.42814528760138
H	-1.83831052021264	-2.44730953171013	5.01679349888770
H	-1.34276641766306	2.88262670098372	3.83373405258490
H	-1.82988821945154	5.07685883221945	2.52344654329430
H	-1.47210986037854	3.84487829308720	-2.79625957103618
H	-2.09240367795781	2.44054364267156	-4.86420612477438
O	-1.41942153181745	-4.42706696427308	2.11429894690527
H	-1.39104760045069	-2.87712680867708	-3.74826288559203
H	-1.30874800243484	-3.82209088745581	2.87952882837671

C	0.07030075527895	1.61966800724322	-2.69960633948129
C	-1.06881871390787	1.78230975148051	-3.48696694498362
C	-1.68062308527978	0.69676805299810	-4.12456704498191
C	-1.12215461629817	-0.57623671047279	-3.97447534385669
C	0.04205149528815	-0.76848273190067	-3.21711349808028
C	0.58403147884968	0.33823454629742	-2.57696647704183
O	-1.59655949342119	3.03460944332716	-3.63499148997562
O	-1.76495718760843	-1.59855357132089	-4.61554319509706
H	1.47438252362254	0.19977340983332	-1.98168930030218
C	0.69889609224747	-2.13184071588545	-3.10162191533460
C	0.11734244624088	-2.89835234511609	-1.92939403239693
C	-0.87397364498146	-3.86061010062705	-2.12059554159122
C	-1.43064572537730	-4.57702688736712	-1.06218428536646
C	-0.96534036587710	-4.31151051549165	0.23461238472527
C	0.05608535205756	-3.38280118621399	0.46379025790648
C	0.54291455455969	-2.67874252471361	-0.62937124118389
O	-1.27096029021270	-4.07721233294702	-3.42669952588232
H	1.31822509059660	-1.94556651110788	-0.45990375487630
C	0.65896779215861	-3.18563323924144	1.84484310650619
C	0.04196865521954	-1.98743470188026	2.53824844210691
C	-1.08211739478808	-2.13588444426343	3.35014500707671
C	-1.68968079753885	-1.05897456154549	3.99286664238354
C	-1.13168065425162	0.21523169773577	3.81545192254730
C	0.01911476375484	0.40302185335245	3.04097973176472
C	0.55211030662064	-0.70662786658134	2.39949981376881
O	-1.56055598315841	-3.42459752568900	3.50974496044726
O	-1.74745663338511	1.24213415594181	4.47227776764011
H	1.43377993559774	-0.56929949291298	1.79292159159578
C	0.68223121651149	1.76407835354170	2.93035466449311
C	0.11842202565658	2.53644387346134	1.75434021484625
C	-0.87737804169870	3.49592334298194	1.93165434497232
C	-1.41543677169646	4.21613164186186	0.86574342526365
C	-0.92826210811481	3.95614998176585	-0.42486672812495
C	0.10043183583247	3.02933110580641	-0.63757109388421
C	0.56712344005743	2.32261191785706	0.46160425340049
O	-1.30184548637830	3.70256045388798	3.23125014289151
O	-1.49497034924067	4.65993020249727	-1.44498083182028
H	1.33889468812891	1.58338715655567	0.30629213023641
C	0.69511148636625	2.81348114237122	-2.01645710673011
C	2.23161593990155	-2.03944142584395	-3.09144685650206
C	2.19429517565695	-3.18049100149938	1.79179812938120
H	0.40493186673945	-4.06689263809797	2.43220253558389
C	2.21469753413320	1.66542609343218	2.94101023204547
H	0.43182502406389	2.31472856893240	3.83610730976381
C	2.22552037320251	2.77298686778050	-1.98926334807803
H	0.43518930631465	3.68770150246983	-2.60791920355661
H	2.51242958311247	-1.23648725357224	-3.77439481333040
H	2.59962512176618	-1.73598204974575	-2.11031703878069
C	2.97445574343793	-3.30138505760069	-3.53019262330361
H	2.49141199530076	-3.88008429067265	1.00981238164970
H	2.57746512419731	-2.20932612303167	1.47613902496690
C	2.89815912513661	-3.60405616174973	3.08112075511094
H	2.48261396426032	0.85759929392674	3.62298393179872
H	2.59700843263021	1.36807233883515	1.96350543041223
C	2.95295306148689	2.92271862380873	3.40057326243656
H	2.57127152179732	3.69458567169211	-1.51445237732552
H	2.57720466623956	1.96199744709434	-1.35162283752285
C	2.89358355411615	2.63329886826609	-3.35581156956852
H	0.45675209172769	-2.68436798785515	-4.00870057708466
H	-2.01949756083529	-4.68876485243126	-3.44680599239656
H	-2.43235948589488	-3.40393004256465	3.92361951118224
H	-1.52897554876418	2.10132612280546	4.06106276210605
H	-2.05092682838259	4.31369880706004	3.24033447097153
H	-1.47140084807315	4.12694525468870	-2.26993678112933
H	-2.22317216296667	2.97152519635692	-4.45462666584883
O	-1.54456622787571	-5.02043879941835	1.24576798670690
H	-1.51085815964767	-2.46360621670040	-4.24013249086366
H	-1.48656696504175	-4.50704881271679	2.07619772319124

C	0.06465311905230	-2.20665895816944	-2.17334284260809
C	1.21622272650998	-2.44079309235841	-2.94079253028556
C	1.69643485909489	-1.47872652414597	-3.83683040051172
C	1.02983605016375	-0.25021242089225	-3.93648210081256
C	-0.12137044893134	0.01253941147221	-3.18469064413465
C	-0.55259369858071	-0.97333415498749	-2.30939842225055
O	1.90504548873528	-3.61945115452554	-2.87989537528805
O	1.55853013513105	0.65697023875456	-4.80881583773396
H	-1.44227806096203	-0.77902508251856	-1.72987016342450
C	-0.88926954881895	1.30952362366941	-3.33527012152410
C	-0.39490028834159	2.35859184025675	-2.36208839617528
C	0.53283808557471	3.31194460189482	-2.78198586423455
C	1.01909028792456	4.30243056749026	-1.91466255855121
C	0.53428628502589	4.32262657855265	-0.60721816502345
C	-0.38514949183069	3.37925225947627	-0.14316737631023
C	-0.81193030961436	2.40853827038980	-1.03922141592042
O	0.97619335323838	3.27023398557224	-4.07241111102130
H	-1.50568548175099	1.65807856397157	-0.68926995363163
C	-0.87680389959531	3.41972847132062	1.29088650264952
C	-0.11486414275823	2.45111279031393	2.17654822844397
C	1.01352056897317	2.85941663100601	2.89897328319213
C	1.68085489623038	1.98575288070803	3.76600279652740
C	1.22232106530169	0.66928218435732	3.88010448178032
C	0.09216089225499	0.22788087876511	3.17832269639042
C	-0.52751264358285	1.13462447833733	2.33053708138433
O	1.51757797532728	4.12628425188479	2.80873804946535
O	1.91274640402554	-0.14972603446393	4.73000889230557
H	-1.40841265746394	0.81196050774575	1.79518758836496
C	-0.45299267892951	-1.17423731057332	3.37281131596529
C	0.12510617641562	-2.14984481164257	2.36686011066242
C	1.19449436611318	-2.98253887133378	2.70609710090770
C	1.74304003946524	-3.88882061105065	1.79908302858322
C	1.17976494817623	-3.95738745256965	0.51446611216775
C	0.10764501296915	-3.14969819071720	0.14067223881753
C	-0.37704497652056	-2.24979244174895	1.07792032826336
O	1.68584303625500	-2.85420152379057	3.99245528634143
O	1.68725497805311	-4.83509494118660	-0.39954573657957
H	-1.19648195202581	-1.60505459621439	0.79440185208371
C	-0.49011614370068	-3.26716675030582	-1.24385106230167
C	-2.40968747205335	1.13438575728309	-3.27718529160046
C	-2.39829541219224	3.26380863808436	1.38352121418210
H	-0.67299978823634	4.42078188121507	1.65902287002022
C	-1.98321035332431	-1.23592619016232	3.41441415873927
H	-0.13356816705540	-1.51147951008886	4.35495648745382
C	-2.02026260091359	-3.30969947843768	-1.22472978356014
H	-0.17639184465689	-4.22856147932999	-1.64204172162784
C	-2.99298631153189	0.15062103030016	-4.28916123462515
H	-2.72549773246119	0.83170791671458	-2.27862297034418
H	-2.85624693099982	2.11851549679971	-3.44281927281608
H	-2.84588903286660	4.00865470502695	0.72032372886937
H	-2.70977490406333	2.29509922746578	0.99324888322331
C	-2.98199866329338	3.43135083791962	2.78406980950947
C	-2.64000145664356	-0.36950949664410	4.48581259058404
H	-2.40550009413493	-0.96669284943206	2.44670378058665
H	-2.26394363817079	-2.27966164194963	3.57923107699942
C	-2.62664300510409	-4.41568783475661	-0.36352865488828
H	-2.42870830857742	-2.35813524757341	-0.88583740208756
H	-2.36159533753560	-3.43058945118766	-2.25611480120374
H	-0.67806201846600	1.69309283515993	-4.32996093633230
C	2.86996291109853	2.46860249270494	4.53730702720022
C	2.93316612692819	-4.75063527700653	2.12145544769577
H	1.39906542252155	4.18685680566861	-4.25166993068161
H	1.24554098721704	4.54016113293856	1.96893254705329
H	1.75669334728380	-1.08505630619319	4.50393967720824
H	2.50835543237472	-3.34736937487399	4.09546792502438
H	2.23660297829112	-5.51267936622087	0.14291353015221
H	1.75578761485305	-4.07577845873504	-2.02774348795610
O	0.94813803502752	5.27488970254124	0.30557857626096

C	2.86245188305969	-4.65963870940859	-1.94032044266777
C	4.60296847922954	-3.31815044231416	-3.13511876635066
C	4.48498491514403	3.17965507668980	-3.48115406461342
C	2.71118518216001	1.94551800015649	-4.74016652519760
C	4.61649253920355	3.44583138078435	3.04800073242630
C	2.82152912275243	4.75081496753885	1.89365489516265
C	4.64909823070714	-3.04494083403932	3.39378203439347
C	2.87320929742864	-1.87306739179835	4.70933596226644
H	2.73709356584277	-3.95356564074822	-3.94407560933252
H	2.78940098436447	-3.88166788903474	4.01115208731549
H	2.75847468238611	4.04573234515643	3.90093424270404
H	2.58934352696305	3.95917733552394	-4.06186799886976
H	1.81358900921093	-4.93859443066573	-1.88022691073878
H	3.16617896680040	-4.32100543089717	-0.94901555982621
H	3.43567185563102	-5.55667630025271	-2.17737148556461
H	5.02763443908911	-2.95146684208292	-2.19872773840884
H	4.80256758778016	-2.57207114965459	-3.90470213882200
H	5.13745427798221	-4.22928057424746	-3.40426052100130
H	4.93292926240854	2.25358937266004	-3.11591297031155
H	4.70278676288773	3.96277151271783	-2.75450933432023
H	4.98342803084840	3.44016979447832	-4.41494662473259
H	1.65232566794977	1.85987046396408	-4.97066296149714
H	3.05065415170962	0.96558626210141	-4.40220814008330
H	3.23932338357718	2.18108312417505	-5.66487926545308
H	5.02615758568811	3.08724422726001	2.10183311805862
H	4.84901558623587	2.70399754408964	3.81243584693862
H	5.13912045750813	4.36737059175324	3.30467905994153
H	1.76652080335732	5.01054521893884	1.85991970100391
H	3.10554852830431	4.41512183711717	0.89548288558006
H	3.38425089251410	5.65867525218329	2.11402357997132
H	5.06284336898709	-2.10319053958318	3.02835032421479
H	4.86986836041118	-3.81387335673983	2.65300875246100
H	5.17721468272175	-3.30209967335694	4.31206323858181
H	1.81956030964399	-1.82683688760899	4.97284702059015
H	3.16737079439897	-0.87884880045891	4.37080456386409
H	3.43692824259720	-2.09818969760475	5.61541519453223
C	-2.75863172055470	0.31140713598906	-5.03408037256740
C	-2.51916580896208	-5.24641174340258	-0.32069175807717
C	-2.57810274552246	-0.34552426253519	5.18516488402919
C	-2.60236607386721	5.25495631235190	0.43320174901248
N	-2.45156822321094	-1.47214297568703	6.12621279650486
N	-2.37553549542601	-6.19309223532503	-1.44054830864502
C	-1.33320560650649	-7.19025670133763	-1.18475916541252
N	-2.74298794572915	1.46914967482047	-5.94371561198865
C	-1.78240070914269	1.29929733118833	-7.03735181795498
N	-2.44275857025343	6.19509798240319	1.55624436793033
C	-1.41364645790889	7.20243118752731	1.28731655220891
H	-3.62914875741278	0.43181179690668	-4.38486289124165
H	-2.90322144308304	-0.61766571358729	-5.58738015525018
H	-3.42644248459233	-4.66855796179946	-0.51129522089551
H	-2.66993291936630	-5.77485119429248	0.62163092539913
H	-3.46971511912125	-0.53688774011060	4.58374565917342
H	-2.74760610869534	0.59149112909877	5.71733834413854
H	-3.49938771826550	4.66676633052916	0.63999872162437
H	-2.77939863277429	5.78834469967461	-0.50173541834237
C	-3.69862407395029	6.84241691112682	1.93060808019540
C	-3.63410252768929	-6.85244201971240	-1.78360026740817
C	-4.06716243039451	1.78338094649724	-6.47710117778662
C	-1.22017670049228	8.08791228594757	2.49482125716895
N	-2.46891923908325	8.73887347131043	2.86448802765217
C	-3.48864048763008	7.73527840573023	3.13226179736670
C	-1.15911453213062	-8.08690551953288	-2.38689480449153
N	-2.41078516396340	-8.74984844549860	-2.72360518648983
C	-3.44242211646066	-7.75570050935574	-2.98044150847819
C	-1.73395721596524	2.54573770400003	-7.88814880332648
C	-4.00248931856337	3.02484699167836	-7.33663152388038
H	-1.59430534716778	-7.79084188572427	-0.30426089105088
H	-0.40152754364911	-6.67066176905542	-0.97133897739688
H	-2.06142000573680	0.43526304595151	-7.65330840910721
H	-0.80134054307626	1.10304110494458	-6.60962346471460
H	-1.69854292374859	7.80997268389337	0.41905944127213
H	-0.48307484065600	6.69216302056969	1.04817286201817
H	-4.42928907771034	6.07136011727013	2.16944351956680
H	-4.08972191268265	7.43225014228547	1.09152605098894
H	-4.37443628299408	-6.08844566282352	-2.01531774556744
H	-4.00475702594530	-7.43635727696033	-0.93120858082269
H	-4.74629528039021	1.94929686675906	-5.64252916056767
H	-4.45272420035033	0.94087581255676	-7.06558070386431
H	-0.47738844989736	8.85097299981819	2.26482972483116
H	-0.82396528188953	7.48212929524704	3.32753398446754
H	-4.42487832732659	8.23750270448917	3.37363231485822
H	-3.21606223742661	7.11058432471133	4.00016808967985
H	-0.40672516797707	-8.84273089973514	-2.16426302930427
H	-0.78392414798538	-7.48777244988148	-3.23404109324115
H	-4.38000585188906	-8.26645388700810	-3.19750673864789
H	-3.19206014889573	-7.13818342593738	-3.86011174243080
H	-1.04246339085035	2.38966360160309	-8.71535164986149
H	-1.34097727638902	3.38113620316042	-7.28395814297741
H	-4.98848655631509	3.22249703735066	-7.75595281140362
H	-3.73507196203854	3.88697840232503	-6.70191221847373
N	-3.05088685734070	2.85728080504286	-8.42549576619362
C	-1.43534714651376	-1.22078891930298	7.15091793152319
N	-2.53996131152310	-2.78428502062803	8.66659312381732
C	-3.54680339113894	-3.03458376468644	7.64564235036423
C	-3.72364592191770	-1.82838270023622	6.75171161238015
C	-1.27510952183683	-2.43263083652149	8.03715080564513
C	-2.39169836697195	-3.92309472483940	9.55478732557020
H	-1.71687137461063	-0.34933070334188	7.75544319284054
H	-0.49289204915920	-0.99518196465517	6.65657489412135
H	-4.49480950583816	-3.26260290220670	8.13179624812542
H	-3.27706848533491	-3.90538208905396	7.02374084844725
H	-4.11202802638161	-0.98440879061679	7.33631247003064
H	-4.44476854732580	-2.05517192090367	5.96810534561822
H	-0.88172597718019	-3.27039513010040	7.43650225911978
H	-0.54163265648253	-2.21526395360650	8.81283656928239
H	-2.05950146789659	-4.83238710059322	9.02910535938467
H	-3.34288806904857	-4.14039726098201	10.03749735400077
H	-1.66045615212273	-3.69428651720143	10.32804366911523
C	-2.24805247272159	-9.64884269651069	-3.85190682267758
C	-3.01116142592355	4.03138118778872	-9.27864269074759
C	-2.28857271302157	9.62725509600315	3.99849419779925
H	-1.49804681430553	-10.40202484682798	-3.61707757779993
H	-1.93267546066516	-9.12751843427121	-4.76966569138607
H	-3.18901137293638	-10.15594362485796	-4.05788505880259
H	-3.32002156425058	3.86451973015196	-10.10297109446321
H	-2.68885356845136	4.93729045907313	-8.74111487608109
H	-3.99960191421194	4.21890815462128	-9.69430146985528
H	-1.54802350169989	10.38741315286135	3.75626704074961
H	-1.95145764922271	9.09840596894900	4.90411735140509
H	-3.22812947842467	10.12644908357735	4.22875694119545
C	2.76558011063833	-4.49836719550846	-2.61288890281965
C	4.46145525402664	-2.99408916638855	-3.66499899630550
C	2.49312114301766	3.74145961116739	-4.31965246744050
C	4.40631846558975	2.57512297244805	-3.18917182517020
C	4.43686054388504	2.61158549154737	3.55810482516047
C	2.76106819405094	4.12635919547523	2.48823328292772
C	4.39682646905783	-3.71869553038707	2.82924850351025
C	2.62920754112111	-2.69170902530400	4.26952716035159
H	2.59806555283680	-3.56985598456386	-4.52358245988237
H	2.52546386494154	-4.60246285708023	3.33583797708060
H	2.56046318599701	3.18441633026808	4.38948581609001
H	2.56965629225799	1.68318273501521	-3.78705936060995
H	1.72593339311214	-4.81125183664424	-2.57330929361412
H	3.07375077476869	-4.26383434066207	-1.59318710565007
H	3.35815642911700	-5.34781849623036	-2.95395925068269
H	4.88806854338208	-2.72103230590987	-2.69796934154272
H	4.63840637690624	-2.16575159711382	-4.35133693528446
H	5.01083795161424	-3.85985933098233	-4.03449818895537
H	2.72429845370616	4.72323414937574	-3.90158074107380
H	1.42896444182114	3.71901615467691	-4.54909963171849
H	3.03224958946834	3.64766312422072	-5.26234464996658
H	4.70104821281853	1.75791550749676	-2.53000612816887
H	4.78698957300293	3.50285553954183	-2.75800837023020
H	4.90346878164772	2.42692441162762	-4.14779102620242
H	4.87903818883605	2.34406573862607	2.59656149612694
H	4.60058672178378	1.77846785405440	4.24190976435551
H	4.98151262268166	3.47381882531720	3.94254937944954
H	1.72269472298036	4.44141742696182	2.43422759939781
H	3.08554020572590	3.89828530968131	1.47207682549646
H	3.34949338202556	4.97228607297087	2.84495803738024
H	4.82003806394060	-2.74623041242507	2.57063474293797
H	4.61318581191776	-4.40349258819918	2.00902084736183
H	4.91895672432069	-4.07988138505293	3.71513293809939
H	1.57518957461858	-2.65740399934297	4.53325792852381
H	2.94570987063643	-1.67034280971633	4.05488435543401
H	3.18207181308991	-3.03497987907076	5.14450891752659
C	-2.94753489179673	0.90910101785646	-4.90363980193836
C	-2.48805195943096	-5.62092506740094	-1.25549067788777
C	-2.88412922086307	-1.23269669452122	4.89044426234144
C	-2.48477291125897	5.25031173373107	1.04379952459846
N	-2.52297670407878	-1.48554763860912	6.29648756931646
N	-1.95833942309750	-6.98045175910068	-1.23879465987279
C	-3.02703139060532	-7.96577956156742	-1.17711015427598
N	-2.91131561414763	2.17906338350532	-5.64931091415811
C	-1.98903854297920	2.12080746164518	-6.78653360552755
N	-1.96714682961915	6.61451447675085	1.04304633403452
C	-3.04274225712988	7.59076386242670	0.96310506929406
H	-3.79194350363901	0.97217475680370	-4.21347133353261
H	-3.14509692367051	0.06952634589614	-5.57161371530234
H	-3.07218977543963	-5.43969651097705	-2.17777011151735
H	-3.20535532614288	-5.54274991156154	-0.44176122421875
H	-3.53648573264065	-2.03448241864947	4.52973543304799
H	-3.49623607146633	-0.33528147404166	4.82694749596954
H	-3.08500062740711	5.06003291600687	1.95400259242032
H	-3.18513444198270	5.16938500531997	0.21568348499028
C	-1.12010607599948	6.89631477157461	2.19058948162435
C	-1.08544726577841	-7.25844959960131	-2.36798124255978
C	-4.23682261138231	2.60968422659634	-6.09095570243639
C	-2.48244829066841	8.99255390669032	0.88133213706192
N	-1.63737703777192	9.28024192953799	2.03174603074403
C	-0.56452727912897	8.29968424871631	2.11284066222683
C	-2.45668945879078	-9.36250606581283	-1.08019814722385
N	-1.58629868934022	-9.64601139420018	-2.21259486354348
C	-0.52011059879455	-8.65691807460816	-2.27493609464453
C	-1.91745330768565	3.46317167160242	-7.47376674777747
C	-4.14653712226784	3.94834862255738	-6.78707510933589
H	-3.68375000173232	-7.90344894488831	-2.06180185447998
H	-3.63956757798648	-7.76236572072580	-0.29909233683218
H	-2.31802136323056	1.35255686175539	-7.49748266937977
H	-1.00439192748648	1.83741564727664	-6.42024611104697
H	-3.71589193160433	7.52072132366842	1.83485903200870
H	-3.63652951344915	7.38492283735352	0.07285538792415
H	-0.29617939658620	6.18576287690850	2.20884706095466
H	-1.68176058692721	6.78170140782972	3.13507351304900
H	-0.26730093613647	-6.54118748976262	-2.37102169576555
H	-1.62828026438099	-7.15111947181286	-3.32416223266279
H	-4.88250057380874	2.69181946800826	-5.21820616429532
H	-4.67528403800934	1.86593146853278	-6.76864551358867
H	-3.30232623960497	9.71045892471111	0.85790055703369
H	-1.92008158910856	9.09977474485353	-0.06165387097518
H	0.03152007924146	8.50230893088678	3.00258675511751
H	0.10807785524698	8.36946388052655	1.24091917407029
H	-3.27079508240396	-10.08721089809959	-1.07144732883804
H	-1.91271591311160	-9.46256627364133	-0.12568269900185
H	0.09494491261351	-8.85707372596906	-3.15220536980580
H	0.13584656508360	-8.71941146942884	-1.38985931600852
H	-1.25760290120605	3.38883018573152	-8.33736745902991
H	-1.47333156799381	4.20030751702155	-6.78314921438574
H	-5.13612562745246	4.23418986609509	-7.14206355545222
H	-3.82444756617021	4.71238349762401	-6.05897294143387
N	-3.23699415632505	3.88490213321720	-7.92159876450620
C	-2.96221971882954	-0.42104911162370	7.18918015010663
N	-2.92249448069960	-1.95220296720709	9.09395577328461
C	-2.48182068694081	-3.01257408775112	8.20004395977795
C	-2.97882023930295	-2.77518867590314	6.79117711534786
C	-2.46093702001948	-0.66478180886442	8.59391531930408
C	-2.47460761031256	-2.18571892839408	10.45417321817348
H	-4.06213361084067	-0.34172565856536	7.20923263509530
H	-2.56982942359744	0.52435866738910	6.82402446150988
H	-2.86899130043108	-3.96440875998259	8.56410175134855
H	-1.38095494356378	-3.08723728855287	8.18285874759051
H	-4.07919541947515	-2.85023062287792	6.77789773150212
H	-2.58685158725568	-3.56738901781514	6.15408358885633
H	-1.35884918646806	-0.60879685541988	8.59766415614016
H	-2.83141231527651	0.12137878258378	9.25198788922709
H	-1.37642367110423	-2.20915903154259	10.54298766628131
H	-2.86171809684320	-3.13817473687044	10.81276199343989
H	-2.84821458816300	-1.39794732681004	11.10630078183874
C	-1.05663917384884	-10.99538654406082	-2.15115468018383
C	-3.17932738525435	5.15175969787684	-8.62825955948676
C	-1.11800069239609	10.63411617689714	1.98425281554029
H	-1.87474009936348	-11.71378274979256	-2.14517791339311
H	-0.44176248978507	-11.17112501818050	-1.25381830285435
H	-0.43821979799381	-11.18918913165134	-3.02608896054198
H	-2.52262083780932	5.06171403865779	-9.49163287440133
H	-2.80477592596604	5.97421548777835	-7.99857478909884
H	-4.17282122523087	5.42222208046089	-8.98137137435221
H	-1.94189066973257	11.34567011781632	1.96498894187610
H	-0.48791599310493	10.81791065777779	1.09912682059684
H	-0.51772821623990	10.83036080810034	2.87119062898164
C	2.91078078571971	-1.72970459591077	-4.67665681301802
H	1.27950616102099	1.56654790359640	-4.58248919062466
C	2.07467386272193	5.25113514349694	-2.41103087396428
H	1.66559480908868	5.80714273375064	-0.05855660899925
N	2.58424436244912	-2.05820434633148	-6.06930119085515
N	1.81375542342342	5.68153512149861	-3.78980780790010
N	2.50441324845379	3.25178814552046	5.72394592206444
N	2.80778611096820	-6.08819750231382	1.52987830950076
H	3.50566129975091	1.61814096198868	4.81260747313201
H	3.46137284502087	3.12378993216282	3.90023795884901
H	3.83446883687727	-4.29940202785137	1.70215440354876
H	3.10464365958110	-4.84332877239823	3.20111429259883
H	3.51513697204768	-0.82427028817882	-4.70287549878206
H	3.51994795708108	-2.51996423466629	-4.22210589838108
H	2.18612580928489	6.13303003182622	-1.76759533849403
H	3.04313162402705	4.74723668537213	-2.41271569491202
C	-4.48572677225966	3.19599458622198	2.74749492193442
C	-2.65794859637397	4.78598710058466	3.39813931195783
C	-4.15373535599465	-0.51628233071061	4.41219092683963
C	-2.12987552804678	-0.67614285936650	5.88642539760368
C	-4.14665586421192	-4.33737755205705	-0.42017279721875
C	-2.14017400741255	-5.80362038954123	-0.75793804376723
C	-4.50309924067398	0.06126499931537	-4.11217812922114
C	-2.63250257664112	0.50671874373091	-5.72489540873008
H	-2.57470878808088	-0.83588910030402	-4.07622648503134
H	-2.53793924962810	2.66394382350440	3.42123074506341
H	-2.39675116985199	0.67162823471226	4.26581463125562
H	-2.32120828546208	-4.23643668048931	0.67083832590574
H	-4.98189976281189	3.94891544432849	2.13214602234785
H	-4.72287043939828	2.21748617103371	2.32966481911850
H	-4.91904148590089	3.24615224752894	3.74631406647873
H	-1.58910328639154	4.92130533330312	3.55449771191838
H	-3.00860331801817	5.59816660006680	2.75802001324131
H	-3.14352605815664	4.89581942805783	4.36769726729305
H	-4.52744730659340	-0.27223927493574	3.41754774813458
H	-4.45843197308451	-1.53973470960996	4.63917195522249
H	-4.64900669997484	0.14278682473561	5.12505056884802
H	-2.26891118062351	-1.73121737390242	6.13135842524680
H	-1.07190433542031	-0.44386156499127	5.99694225071322
H	-2.66966914548192	-0.09022594869212	6.63021502857896
H	-4.50536494646234	-3.35278087222542	-0.11866352651617
H	-4.50703787872138	-4.52566115141424	-1.43319763973619
H	-4.60541462696660	-5.07654401172813	0.23658402817140
H	-2.34291644759218	-6.00137410301229	-1.81232834111069
H	-1.07056526710057	-5.92383991653136	-0.59621556825624
H	-2.64727971114503	-6.57159464002454	-0.17369844636095
H	-4.76636915705915	-0.22191712382626	-3.09249912091101
H	-4.97839035220296	1.02095927679145	-4.32380295808856
H	-4.93581000980291	-0.67798494828237	-4.78627035116228
H	-2.95584022572046	1.52116671943168	-5.96699479110393
H	-1.56060838380621	0.44512123474130	-5.90451567612041
H	-3.11890461286637	-0.17222213180178	-6.42522268419784
C	1.85963652573231	-3.31341740090107	-6.19530220762479
C	3.78384391715344	-2.09924219620315	-6.88952015691190
C	1.76766665301573	-6.88943309007093	2.18166210734361
C	4.07747824803321	-6.81499396976403	1.51228752030030
C	2.98202698296413	6.31136379816225	-4.40564837427003
C	0.64742996536201	6.56468869873993	-3.88409917347379
C	1.80348112189392	2.45927194963158	6.72397877352288
C	3.68206567892926	3.86319197539609	6.31999657464644
H	0.94866596703244	-3.25776051379255	-5.60285898104563
H	2.45246427588201	-4.15891324891482	-5.80909310283952
H	4.29511962973424	-1.13946312761680	-6.81278872204441
H	4.48576063620104	-2.87627593849525	-6.53934758685290
H	0.82891969759090	-6.34174405141755	2.13873453214144
H	2.02154135789054	-7.04643903215840	3.23751474023615
H	4.81209920638437	-6.21534325290178	0.97768796833499
H	4.44321033581918	-6.96915097220172	2.53549656168416
H	3.26697404834853	7.21293083301605	-3.84827311029223
H	3.81461408189082	5.61140772545765	-4.36474606437155
H	-0.21233480836122	6.05226975681312	-3.45789862489081
H	0.82046414839700	7.47758328289505	-3.30017443860020
H	0.90605902327842	2.03662837814400	6.27674992496999
C	1.41702879368279	3.31711234743997	7.90645834800540
H	4.17319746466372	4.48455511694784	5.57069439583921
C	3.30040636415901	4.71885232335152	7.50657520730325
C	1.50190480227487	-3.57359025851542	-7.64038732289130
C	3.43372601792181	-2.36468459946856	-8.33663227051223
C	1.61902992184117	-8.21987458373511	1.48333915055652
C	3.90532775281150	-8.14836952008387	0.82211301731672
C	2.68598337049192	6.67141104179357	-5.84300950066642
C	0.37446285022358	6.91728140193103	-5.32655576954290
H	2.42463895201915	1.61900242117182	7.07454456627642
H	4.41041336957687	3.09918970571420	6.64284155771417
N	1.53428845646245	7.55741380337503	-5.93034536403445
N	2.69656913462944	-3.61295735925775	-8.47297925616678
N	2.88073468434967	-8.94583540954393	1.47961622573865
N	2.59221047915267	3.93312302887307	8.50705830963641
H	0.92313360061180	2.69845948115474	8.65626632018956
H	0.69168859705271	4.08201112400897	7.57954268353644
H	0.80300262062336	-2.79177572505499	-7.98469295578463
H	0.98733093717609	-4.53138380530404	-7.72096564004658
H	4.34819743023362	-2.42948437073296	-8.92702039256883
H	2.84785105470987	-1.51412123903288	-8.72582096034860
H	1.25139395198576	-8.05099282075231	0.45704698453702
H	0.86927815973231	-8.81464095837052	2.00355491693784
H	2.52344194404904	5.74638427878568	-6.42162655045530
H	3.55305741698228	7.17410625334874	-6.26989257944268
H	-0.47228159961810	7.60071064774575	-5.37381268295842
H	0.09115881146962	6.00247785804391	-5.87393328769820
H	2.68375507125745	5.56435408404598	7.15602270476119
H	4.20066271430700	5.13580000235461	7.95876955184956
H	3.65730659653077	-7.97475073764940	-0.23873128420142
H	4.85002791434133	-8.68974661836023	0.85214701473570
C	2.24083552069403	4.73554713498650	9.66281518404175
H	1.74651979946204	4.11379007453919	10.40775896438556
H	1.56485846597187	5.57004728468781	9.41407017794378
H	3.14099455570687	5.15409956344134	10.11035447865550
C	1.26987927993336	7.94906084183481	-7.30362861877585
H	2.13870462481196	8.45992116569713	-7.71460329410332
H	0.42453729481188	8.63403859485673	-7.33439896890728
H	1.03876978349112	7.09056881259374	-7.95330512852741
C	2.36922447716625	-3.88683488429178	-9.85895003714469
H	3.28172460003959	-3.95540527757736	-10.44945660596683
H	1.73077866551199	-3.10841978553465	-10.30803081068683
H	1.84247232964711	-4.83701380072172	-9.93272199628171
C	2.74120428244600	-10.24967707502019	0.85557113769445
H	1.99466625619837	-10.83480079684265	1.38946104845387
H	3.68926284053531	-10.78244076227372	0.90037426489850
H	2.43422829302615	-10.18509717459632	-0.20003147437358
